# Genomic Architecture of Phenotypic Plasticity in Response to Water Stress in Tetraploid Wheat

**DOI:** 10.3390/ijms22041723

**Published:** 2021-02-09

**Authors:** Andrii Fatiukha, Mathieu Deblieck, Valentyna Klymiuk, Lianne Merchuk-Ovnat, Zvi Peleg, Frank Ordon, Tzion Fahima, Abraham Korol, Yehoshua Saranga, Tamar Krugman

**Affiliations:** 1 Institute of Evolution, University of Haifa, Haifa 3498838, Israel; avfatyha@gmail.com (A.F.); valentyna.klymiuk@usask.ca (V.K.); tfahima@univ.haifa.ac.il (T.F.); korol@evo.haifa.ac.il (A.K.); 2 Department of Evolutionary and Environmental Biology, University of Haifa, Haifa 3498838, Israel; 3 Julius Kühn-Institut (JKI) Federal Research Centre for Cultivated Plants, Institute for Resistance Research and Stress Tolerance, 06484 Quedlinburg, Germany; mathieu.deblieck@julius-kuehn.de (M.D.); frank.ordon@julius-kuehn.de (F.O.); 4 R. H. Smith Institute of Plant Science & Genetics in Agriculture, The Hebrew University of Jerusalem, Rehovot 7610001, Israel; lianne.ovnat@mail.huji.ac.il (L.M.-O.); zvi.peleg@mail.huji.ac.il (Z.P.); shuki.saranga@mail.huji.ac.il (Y.S.)

**Keywords:** drought resistance strategies, flowering phenology, genomic architecture, linear regression, phenotypic plasticity, QTL analysis, wild emmer wheat

## Abstract

Phenotypic plasticity is one of the main mechanisms of adaptation to abiotic stresses via changes in critical developmental stages. Altering flowering phenology is a key evolutionary strategy of plant adaptation to abiotic stresses, to achieve the maximum possible reproduction. The current study is the first to apply the linear regression residuals as drought plasticity scores while considering the variation in flowering phenology and traits under non-stress conditions. We characterized the genomic architecture of 17 complex traits and their drought plasticity scores for quantitative trait loci (QTL) mapping, using a mapping population derived from a cross between durum wheat (*Triticum turgidum* ssp. *durum*) and wild emmer wheat (*T. turgidum* ssp. *dicoccoides*). We identified 79 QTLs affected observed traits and their plasticity scores, of which 33 reflected plasticity in response to water stress and exhibited epistatic interactions and/or pleiotropy between the observed and plasticity traits. *Vrn-B3* (*TaTF1*) residing within an interval of a major drought-escape QTL was proposed as a candidate gene. The favorable alleles for most of the plasticity QTLs were contributed by wild emmer wheat, demonstrating its high potential for wheat improvement. Our study presents a new approach for the quantification of plant adaptation to various stresses and provides new insights into the genetic basis of wheat complex traits under water-deficit stress.

## 1. Introduction

Water stress is one of the main abiotic factors affecting plant growth and limiting crop production. Global climate changes increase the frequency of extreme drought events in many regions, thus becoming a severe threat to food security [1,2,3]. Wheat is one of the most important crops worldwide, providing about 20% of calories and proteins in the human diet [4]. Drought affects more than 42% of the worldwide wheat production area [5]; hence, the improvement of drought resistance in wheat cultivars is among the main targets for wheat breeders.

Crop wild relatives developed adaptation mechanisms to cope with water-limited conditions that can be used for crop improvement [6]. Wild emmer wheat (WEW) (*Triticum turgidum*, spp. *dicoccoides*), the ancestor of cultivated wheat, offers an important reservoir of genetic variation for useful traits. It may be used to increase the genetic diversity available to breeders for wheat improvement, including resistance to abiotic and biotic stresses [7,8,9,10,11]. Previously, we evaluated WEW populations, representing an aridity gradient across Israel and vicinity, and revealed high diversity for drought stress tolerance with some genotypes displaying better performance under drought than durum wheat (*Triticum turgidum* ssp. *durum*) cultivars [9]. Then, we developed a recombinant inbred lines (RIL) mapping population, derived from a cross between durum wheat and WEW, and genetically dissected drought-adaptive loci [10]. Subsequently, several WEW chromosomal regions conferring increased yield and drought-adaptive traits were introgressed into wheat cultivars using a marker-assisted selection (MAS) approach [12,13,14]. We also conducted whole transcriptome analyses of drought-tolerant versus drought susceptible accessions of WEW in response to water stress and identified potential candidate genes that may be associated with drought tolerance [15,16].

The complex responses of plants to water stress encompass multiple physiological, cellular, and biochemical processes, coordinated by a large number of genes [17]. Due to the complex quantitative mode of inheritance of traits involved in the response to drought stress and their effect on productivity traits, unraveling the genomic architecture of these traits is crucial for further progress in this field. Currently, the most suitable approach for genetic dissection of complex traits, such as drought resistance, is quantitative trait loci (QTL) analysis [17,18,19,20]. The QTL analysis of physiological drought adaptive traits (DAT) associated with response to drought can be used for genetic dissection of drought resistance strategies such as escape, avoidance, or tolerance, as demonstrated in recent publications [21,22,23,24]. However, only a few studies have focused on the effect of physiological traits on productivity, considering the interaction between yield-related traits and DAT [25,26,27,28].

Phenotypic plasticity is the ability to respond to environmental changes by altering the phenotype [29,30]. The identification of genomic regions that confer phenotypic plasticity in response to abiotic stress can be dissected by: (a) testing of QTL-by-environment interactions [31]; (b) mapping of QTLs for plasticity response [22,32]; (c) using a multi-environmental approach (MEA) in QTL analysis [33]; or (d) QTL mapping of a susceptibility index calculated for each trait [10]. Changes in flowering phenology play an important and decisive role in plant development and plasticity in response to water stress [34,35,36]. Therefore, to reduce various biases in QTL analysis, the influence of flowering time should be considered when analyzing other traits. The simplest way to solve this problem is to use mapping populations with a narrow distribution of flowering time. Alternatively, the mapping population can be divided into smaller subsets of individuals by their range of flowering [25]. Another approach is to include various quantitative adjustments of variation in flowering time for QTL mapping of other traits [37,38,39,40]. Previously, deviations from the regression line (i.e., residuals) were defined as drought-resistant indexes for a set of pearl millet cultivars, independently of the effect of heading time and yield potential under control conditions [41]. However, despite its simplicity, this approach has not been used in QTL mapping.

In the current study, we applied QTL mapping of phenotypic plasticity of complex traits under water-limited conditions using a recombinant inbred line (RIL) population derived from a cross between durum wheat cv. Langdon (LDN) and drought resistant WEW (accession G18-16). For QTL analysis, we targeted groups of traits related to (a) yield; (b) phenology; (c) morphology; (d) biomass; and (e) DAT. We employed residuals of the linear regression between values of traits in control and stress conditions as drought plasticity traits. The wide distribution of heading time in the population was considered as a source of various biases in QTL analysis of the traits and was adjusted using the same regression approach. We further used the whole-genome assembly of WEW [42] to localize candidate genes (CGs) associated with the studied traits, residing within the QTL intervals. We particularly gave attention to those CGs that are involved in the regulation of flowering and plant development.

## 2. Results

### 2.1. High-Density Genetic Map

Genotyping of the RIL population, derived from a cross between durum wheat LDN and WEW accession G18-16 (hereafter referred to as G×L population), followed by quality control, resulted in 4347 polymorphic single nucleotide polymorphism (SNP) markers. Out of these, 4015 SNPs representing 1369 unique loci (skeleton markers) were clustered into 14 linkage groups (LGs) (Figure S1). The genetic map covered 1835.7 centimorgan (cM) (953.1 cM for the A genome and 882.6 cM for the B genome) (Tables S1 and S2). The number of skeletal markers (and length of individual chromosome maps) ranged from 51 (84.6 cM) for chromosome (Chr.) 4B to 146 (165.3 cM) for Chr. 5B. A relatively high proportion (6.3%) of non-recombinant chromosomes was observed among 150 × 14 = 2100 RIL × chromosome combinations (Table S3). A total of 311 (22.7%) skeletal loci showed significant (*p* ≤ 0.05) segregation distortion (Figure S2), more frequently in favor of WEW rather than domesticated parent allele (203 vs. 108, respectively). The order of markers on the current genetic map showed highly similar positions on the WEW pseudomolecules (average rank correlation coefficient 0.999) (Table S4).

### 2.2. Relationships between Phenotypic Traits

Four sets of phenotypic traits were used in the present QTL analysis. One set of observed traits (e.g., measured in the field or the lab), and three sets of derivative traits: traits that were adjusted for phenology, or drought plasticity traits I and II (with and without adjustment for phenology), as described in M&M. The observed set included 17 traits, of which 13 were previously measured in the population under water-limited (WL) and well-watered (WW) conditions [10,43]. The normal distribution of most of the observed (excluding leaf rolling (LR)) and derivative quantitative traits of the RIL population was observed in each of the two irrigation regimes (Figures S3–S5, Table S5). Most of the observed and phenology derivative traits (dftraits) showed a wider distribution under WW than under WL conditions (Table S6). A similar range of variation was observed in the two irrigation regimes for the observed and traits adjusted for the effect of heading (dftraits) of harvest index (HI), flag leaf width (FLW), LR, and osmotic potential (OP). Both phenological traits (DP-H and DH-M) and chlorophyll content (Chl) exhibited a wider range under WL (Table S6). The analysis of variance (ANOVA) showed highly significant effects (*p* ≤ 0.001) of irrigation regimes for most of the traits (Table S7), except spike length (SpL) and flag leaf length (FLL) (0.01 ≤ *p* ≤ 0.05). The genotype effect was highly significant (*p* ≤ 0.001) for most of the traits, except for vegetative dry matter (VegDM) and total dry matter (TotDM) (0.001 < *p* < 0.01) (Table S7). The irrigation × genotype interaction was found to be significant only for DH-M and kernel number per spike (KNSP).

**Figure 1:**
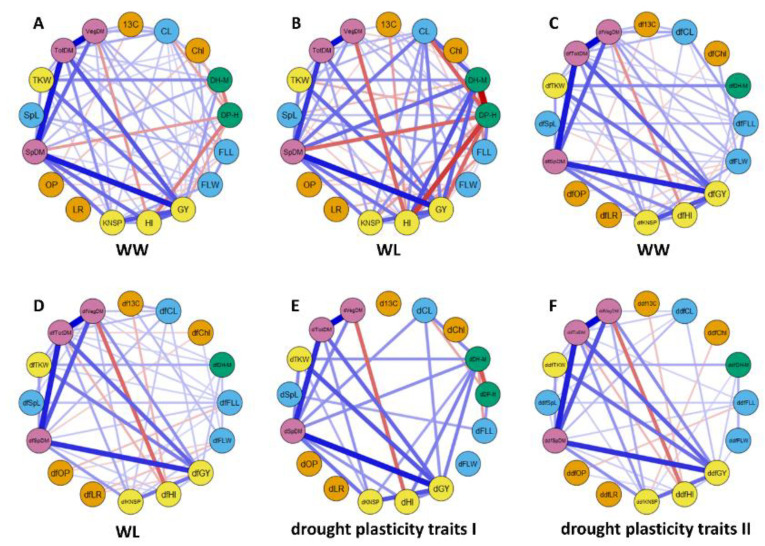
Phenotypic relationships between the analyzed traits based on correlation network analysis in 150 RILs of the G×L population. The correlations between traits are shown separately for WW and WL treatments: (**A**,**B**) for observed traits; (**C**,**D**) for traits adjusted for the effect of heading (prefix df to the name of the trait). Correlations within the group of plasticity traits without (drought plasticity traits I prefix d to name of the trait) and with adjustment for the effect of heading (drought plasticity traits I prefix ddf to the name of the trait) are presented in (**E**,**F**), respectively. Green hexagons represent a group of yield-related traits: grain yield (GY), thousand kernel weight (TKW), kernel number per spike (KNSP), and harvest index (HI); blue squares–a group of biomass related traits: spike dry matter (SpDM), vegetative dry matter (VegDM) and total dry matter (TotDM); pink rhombuses–group of morphology-related traits: culm length (CL), spike length (SpL), flag leaf length (FLL) and flag leaf width (FLW); yellow circles–drought adaptive traits: carbon isotope ratio (δ^13^C), osmotic potential (OP), chlorophyll content (Chl) and flag leaf rolling (LR) and red octagons–phenology related traits: days from planting to heading (DP-H) and days from heading to maturity (DH-M). The width of lines represents the strength of correlation (minimum level of correlation is 0.16), red and blue colors correspond to the positive and negative association, respectively.

Correlation analyses were performed for the four groups of data: observed traits, traits adjusted for phenology, and drought plasticity traits I and II (with and without adjustment for phenology, respectively) (Tables S8–S10, Figure 1). The strongest negative correlation was observed between two observed phenological traits, DP-H and DH-M: −0.93 in WL and −0.46 in WW. Positive correlations were observed between the observed yield-related traits, biomass related traits, DH-M, and culm length (CL) in both treatments. These traits showed a negative correlation with DP-H under both conditions, with a stronger correlation in the WL. This trade-off between developmental periods from planting to heading (DP-H) and from heading to maturity (DH-M) indicates the strong interactions of these two phenology-related traits with most of the other traits. The relationships between morphological traits and yield/biomass related traits showed different patterns in WW and WL conditions. For example, CL and FLW showed a positive correlation with VegDM in the WW (0.29 and 0.28, respectively), but no correlation in the WL. FLL and CL showed opposite directions of correlation under WW and WL (0.29 and −0.20, respectively). Most of the physiological traits were poorly correlated with the traits of the other groups. All observed traits under WW were positively correlated with corresponding traits under WL (Table S11) with the lowest association for OP (0.18) and the strongest association for DP-H (0.85). Interestingly, variations in the traits between treatments had a strong negative association with the values of traits under WW (Table S11).

The correlations of the derivative traits showed common patterns with those of the observed traits, with few exceptions (Tables S8–S10, Figure 1). Notably, no significant correlations of drought plasticity traits I of DP-H (dDP-H) with most plasticity traits were found. However, the drought plasticity traits I of DH-M (dDH-M) was positively associated with productivity and yield-related traits, confirming the importance of grain filling stage duration mainly in water-limited conditions. Rank correlations (Kendall’s tau) between the observed and adjusted for heading traits (dftraits) (Table S12) were stronger for traits obtained under WW conditions compared to those obtained under WL conditions, suggesting that the influence of heading date on other traits was stronger under WL conditions. The relationships between the observed traits and dftraits showed common patterns, for both WW and WL conditions. The ranks of genotypes for the dftraits were slightly different from those of the observed traits when the observed traits were uncorrelated to heading date, whereas, for traits highly correlated with heading date, the ranks of genotypes for dftraits are considerably changed. For example, the rank correlation between DH-M and adjusted for phenology DH-M (dfDH-M) in WL conditions was only 0.18, since DH-M is highly correlated with the heading date (−0.93).

### 2.3. Genomic Dissection of Initial and Derivative Traits

**Figure 2:**
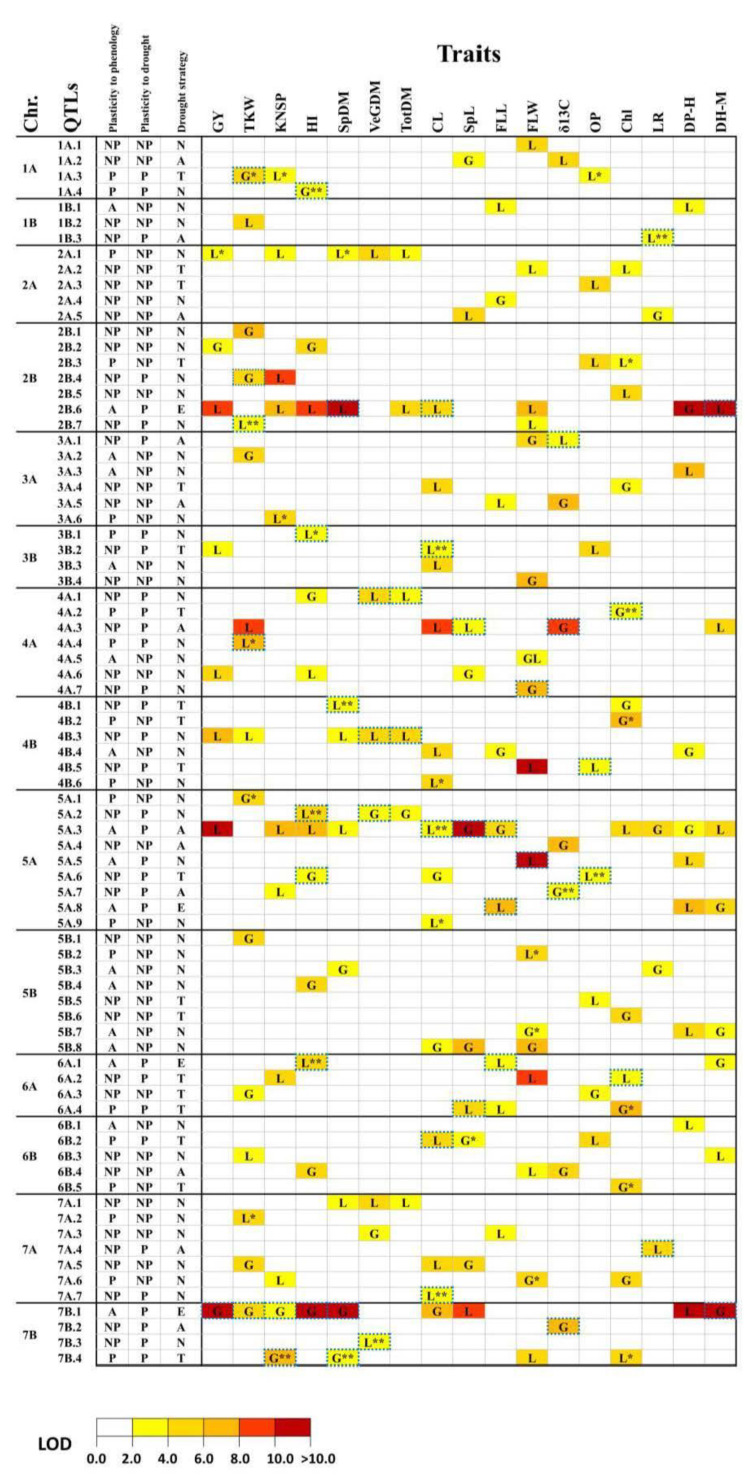
Genetic architecture of 17 traits and their relationships with phenology and plasticity to drought stress. According to our classification, QTLs were marked as follows: non-plastic (NP); plastic (P) and associated with heading (A). Concerning drought resistance strategies the QTLs were marked as escape (E); avoidance (A); tolerance (T); and ‘no associated strategy’ (N). The origin of increase trait value (ITV) allele is indicated as G for WEW (accession G18-16) and L for LDN. QTL effects only on adjusted for phenology were marked by one asterisk (*), only on plasticity traits to drought with two asterisks (**). QTLs with effects on drought plasticity traits were marked by a blue dash border. LOD scores were coded according to higher LOD scores in cases of co-localization QTL effects of corresponding observed and derivative traits.

QTL analysis was performed for the 17 observed traits and 49 derived traits. Among the derived traits, 16 resulted from adjustment for the effect of phenology, and 33 are considered here as drought plasticity traits. In total, we detected 291 significant QTL effects distributed among 79 putative QT loci (Tables S13–S14, Figures S6–S19), out of which 44 revealed a pleiotropic effect on two or more traits and 35 affected only one trait (Table 1 and Table S14, Figure 2). About one-third of the 79 mapped loci had QTL effects only on the observed (13) or derivative (15) traits, while most loci (51 out of 79) included QTL effects on both observed and derivative traits (Tables S13–S14 and Figures S6–S19).

### 2.4. Genomic Dissection of Traits Adjusted for Heading Time

**Figure 3:**
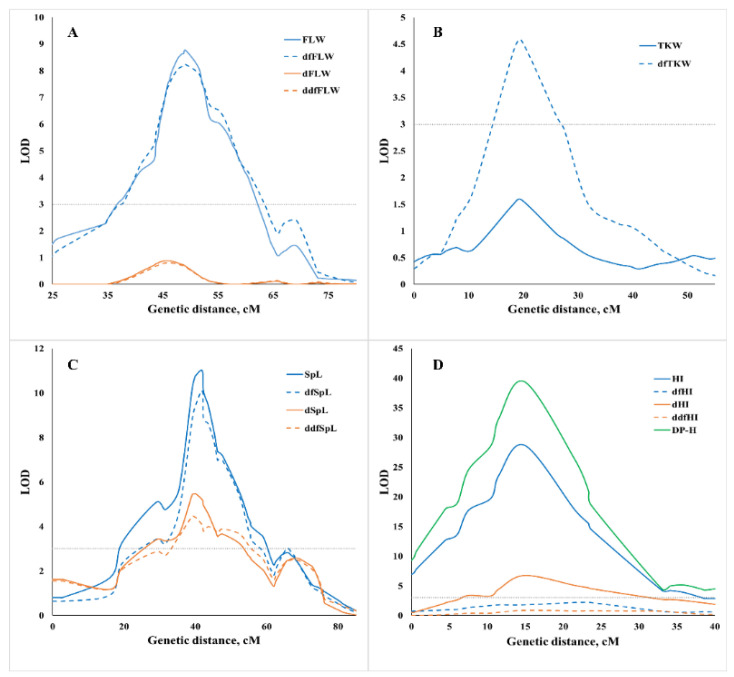
Examples of classification of the detected QTLs in relation to phenology and drought plasticity: (**A**) non-plastic to phenology and drought QTL 6A.2; (**B**) plastic to phenology QTL 7A.2; (**C**) non-plastic to phenology and plastic to drought QTL 5A.2; (**D**) associated to phenology and related to drought escape strategy QTL 7B.1.

The presence/absence of QTL effects for derivative traits adjusted for the effect of phenology (dfQTLs) was used for the classification of QTLs as ‘plastic’ or ‘non-plastic’ concerning variation in phenology (Table 1, Tables S13 and S14, Figure 2). Most of the QTLs (43 out of 79) showed significant effects on both observed and derivative traits. For 28 of these 43 QTLs, both effects were rather similar (Tables S13 and S14). Therefore, we defined them as ‘non-plastic’ concerning the variation of heading date (Figure 3). The group of QTLs classified as ‘plastic’ comprises the following categories: (i) 13 dfQTLs that affected only the derivative traits (Figure 3); and (ii) 6 QTLs that displayed pleiotropic effects on additional traits only after adjustment for heading time (Table S13). Most of these 13 dfQTLs had an effect on a single trait only, excluding QTL 1A.3 that affected TKW, KNSP, and OP. A total of 10 QTLs had effect on DP-H and 7 other QTLs displayed full suppression of QTL effects after adjustment for heading time. Those loci were marked as ‘associated’ with heading (Figure 3).

### 2.5. Genomic Dissection of Drought Plasticity Traits

QTLs were defined as ‘non-plastic’ concerning drought when significant effects were revealed for the observed traits, while no effect for drought plasticity traits drought plasticity traits I and II (‘dtraits’ and/or ‘ddftraits’) was detected in the same QTL region. On the contrary, QTLs that affected only drought plasticity traits without displaying significant effect on the observed traits or QTLs with co-localization of effects on both observed and derivative traits were classified as ‘plastic’ (Table S14, Figure 3). According to this approach, we identified 33 QTLs with plastic drought effects on at least one trait (Table 1 and Table S14, Figure 3): 9 QTLs for dtraits; 11 QTLs for ddftraits, and 13 QTLs for combinations of dtraits and ddftraits. These results highlight the importance of adjusting for the effect of heading time in QTL mapping of plasticity to water availability and adaptation to drought. Most of the plastic QTL effects (33 out 49) were collocated with corresponding observed traits, while 15 QTL effects presented 14 QTLs affected on drought plasticity traits only. A major plastic QTL 7B.1 for the response to drought-affected six dtraits (dGY, dTKW, dKNSP, dHI, dSpDM, and dDH-M) with ITV allele contributed by G18-16. The highest number of plastic QTLs (six) was found for HI.

A comparison of QTL effects for plasticity to drought conferred by the G18-16 and LDN increased trait value (ITV) alleles is presented in Table 2. In most cases, the ITV alleles for the plasticity QTL effects on the yield-related traits (GY, TKW, KNSP) were contributed by G18-16. For HI and SpDM, the number and proportion of explained variation (PEV) scores of plasticity QTL effects, were very similar for ITV alleles contributed by both LDN and G18-16 (Table 2), while ITV alleles for plasticity QTL effects on VegDM and TotDM were provided mainly by LDN. Plasticity of morphological traits showed different origins of ITV alleles: equal for LDN and G18-16 for SpL; higher for LDN alleles for CL; higher for LDN for flag leaf length trait, but higher for G18-16 for flag leaf width trait. The QTL analysis of plasticity of two DATs (δ^13^C and OP) showed that alleles for higher adaptability originated from G18-16. QTLs for the plasticity of Chl had an equal number of ITV of G18-16 and LDN alleles. The plasticity of LR was associated with the ITV allele of LDN in the two detected QTLs, suggesting the high importance of LR plasticity for plant adaptation to water deficit in LDN, which has wider leaves than G18-16.

### 2.6. QTLs Associated with Drought Resistance Strategies

We attempted to use QTL effects on four DATs in order to classify the QTLs in relation to drought resistance strategies, considering that OP and Chl are associated with drought tolerance strategy (i.e., indicating the extent of photosynthetic apparatus damage caused by the water-deficit stress) and δ^13^C and LR with avoidance strategy. A total of 33 QTLs fell into these categories, out of which 17 were designated as plastic (Table 3). Nevertheless, most of these QTLs (67%) did not affect yield-related traits. The ITV alleles for most of QTLs affecting observed and plasticity DATs originated from G18-16 (79% and 65%, respectively). Four major plastic drought QTLs were associated with drought avoidance: three for δ^13^C (3A-1, 4A-3, and 7B-2 with G18-16 ITV allele) and one for LR (7A-4 with LDN ITV allele) (Tables S13 and S14). A major QTL effect on OP was identified on Chr. 4B (QTL 4B-5) with the G18-16 allele associated with drought tolerance (lower OP values). This QTL had also a strong effect on FLW with LDN ITV allele (Tables S13 and S14). In the current study, we have classified QTLs as ‘drought-escape’ QTLs if they showed significant effects on drought plasticity I (dtraits), but no effect on the corresponding ddftraits for drought plasticity II, (Table 3, Figure 3).

### 2.7. Candidate Gene Analysis

More than 95% of the SNPs (3824/4015) from our G×L genetic map were anchored to the reference genome of tetraploid WEW [42] (Table S2). The physical and genetic positions of these SNP markers (Table S2) enabled us to define the physical intervals of QTLs and the contents of genes within these intervals (Table S15). The physical intervals of QTLs ranged from 3.15 to 487.85 Mbp and the number of genes within these intervals varied from 25 to 2136 (Table S16). Most of QTLs with large intervals (>100 Mbp) were located in pericentromeric regions. On the contrary, 16 QTLs with small physical intervals (<10 Mbp) and relatively low gene content (Table S16) were dispersed along with different chromosome parts, except for the pericentromeric regions. Most QTLs with more than 200 genes within intervals were excluded from the CG analysis. Our search for CGs was focused on known genes associated with studied traits, regulation of flowering, and development (genes related to hormonal pathways and biosynthesis). We identified 53 potential CGs within our QTL intervals (Table S17). The putative genetic positions of CGs on the QTL map are shown in Figures S6–S19. The list of the candidates includes six CGs with well-known effects on the studied traits, *Glu-B3* (TKW), *TaCly1* (SpL), *Wx-B1* (GY), *Wx-A1* (TKW), *WAP2-B* (SpL), and *Gpc-B1* (DH-M, TKW). A total of 11 CGs related to phenology were identified within 8 QTL intervals (Table S17). Around half of all CGs (26) were associated with the regulation of hormonal balance: 13 CGs identified within eight QTL intervals were related to signaling and biosynthesis of gibberellin (GA) (Table S17); 11 CGs and located within ten QTLs were associated with the ethylene signaling pathway, and two CGs found within two QTLs were associated with regulation of auxin. Besides, we identified two heat stress associated CGs (*HSFA2C* and *HSP22.0*) within two QTLs, three genes related to transport of nitrate (*NRT2.6*) and sugars (*STP1* and *SUT4*) within two QTLs affecting TKW, and a cluster of seven CGs with NAC domain within interval of QTL 2A.7 affecting chlorophyll content.

## 3. Discussion

Phenotypic plasticity is one of the main mechanisms of adaptation to abiotic stresses via changes in critical developmental stages, such as the timing of the transition from vegetative to reproductive growth [34] and also by affecting physiological traits [35]. Altering flowering time is an evolutionary strategy adopted by plants to cope with environmental stresses, such as drought, to ensure maximum reproduction under changing environments [36]. Diversity for plasticity is commonly found in wild plants adapted to their environments. However, the genetic diversity of many crops was eroded during domestication and subsequent improvement under domestication, due to the one-sided selection for increasing yield that reduced adaptability of cultivars [44]. The present dissection of the genomic architecture of agronomic and physiological traits plasticity in response to drought is demonstrating the effect of heading time on adaptation to WL conditions. The comprehensive genetic analysis of the observed traits and their derivatives, based on regression residuals, enabled us to identify plasticity QTLs and tentatively classify them into several drought adaptation strategies.

### 3.1. Detection of QTLs Using a High-Density SNP-Based Genetic Map

Several QTL mapping studies were previously conducted based on a genetic map constructed by genotyping of the G×L RIL population with SSR and DArT markers [45]. These studies included the genetic dissection of drought resistance [10], grain protein content (GPC), and grain micronutrient content [46], and domestication related traits [43,47]. Genotyping of this RIL population with a high-throughput SNP array allowed us to achieve a shorter map (1836 cM for SNPs vs. 2317 cM of SSR-DArT map) and considerably increase the number of ordered polymorphic markers. Our current map includes an over four-fold higher amount of skeletal (framework) markers (1369 vs. 307 in the previous map) and five-fold smaller average interval lengths between adjacent markers (1.3 cM vs. 7.5 cM). In the current SNP map, the short arms of Chr. 3A, 4A, 5A, 5B, and 7A are extended and the short arm of Chr. 4B is fully present, while it was completely absent in the previous map. Our results confirmed the observed earlier patterns of an increased amount of non-recombinant chromosomes and segregation distortions for different chromosomes in this population [45]. The current SNP-based map allowed us to identify new QTLs, with an improved overlap of QTL effects and shorter QTL intervals. For example, on chromosome 7AS we detected two linked QTLs affecting biomass related traits, the first distal QTL (7A.1) had effects with ITV allele of LDN and the second QTL (7A.3) had ITV allele of G18-16 for SpDM. The second QTL was previously detected [10], and the corresponding genomic region from G18-16 was introgressed into hexaploid wheat (*T. aestivum*) cultivars using MAS [12,13,14]. The introgression resulted in an improved GY and biomass under water-deficit. Identified closest SNPs of target QTLs will improve the accuracy and efficiency of MAS to modern wheat cultivars.

### 3.2. Complexity of Quantitative Trait Genetic Architecture and the Interaction with Altered Phenology

Correlation analysis and the obtained results of QTL analysis demonstrate the complexity of the studied traits and their intra- and inter-group relationships. For example, all the detected QTLs for GY, SpDM, TotDM, SpL, and DP-H traits conferred pleiotropic effects on other traits and did not show even one case of a single-trait-only QTL effect. Moreover, the average proportion of single-trait QTLs for the remaining 12 traits was also very low (~20%). Three major QTLs for phenological traits (2B.6 and 7B.1, and 5A.3), showed strong pleiotropic effects on many other traits, with trade-off relationships between them. Furthermore, these effects were considerably stronger under WL conditions. A similar trade-off related to the influence of phenology was found in a collection of Old-World lupines (*Lupinus* sp.) [48]. The wide range of heading in the studied population and strong association of phenology and other traits found in this study at the genotypic (pleiotropic effects) and phenotypic (correlation) levels, required taking special measures to avoid potential biases in the QTL analysis. Our results demonstrate that regression analysis for adjustment to heading date, enabled us to identify the relationships between the mapped QTLs and phenology. Furthermore, it enabled to increase the QTL detection power that led to identification of QTL effects of phenology adjusted traits that were not significant for the observed traits as well as higher LOD scores of QTL effects of phenology adjusted traits compare to LOD scores of observed traits in some co-localized QTLs.

### 3.3. Drought-Plasticity and Drought-Resistance Strategies

A variance ratio and a slope of norm reaction serve as two main methods of the phenotypic plasticity quantification [49]. The reaction norm was used as a measure for plasticity traits to identify QTLs associated with barley performance in response to aphids and rhizobacteria [50]. However, our approach can be applied as an alternative to these methods with two advantages: (i) normalization of drought plasticity scores for variation in the trait under non-stress conditions, (ii) accounting for the variation in additional important factors, for example, phenology. Linear regression residuals were previously used for various purposes in the analysis of quantitative variation, such as the exclusion of the effect of phenology on the performance of pearl millet (*Cenchrus americanus*) cultivars under drought conditions [41] and characterization of the impact of heat, corrected for differences in the size of the siliques between Arabidopsis accessions in control conditions [51]. Nevertheless, it seems that the current study is the first to apply this approach that considers the variation in phenology to reduce biases in mapping plasticity QTL. Although we used a different approach to map plasticity QTLs, we also found co-localization of QTL effects for the observed and the plasticity traits, as well as the presence of separate QTLs affecting only plasticity traits similar to those found in barley [50]. This phenomenon may have resulted from pleiotropic and/or epistatic effects in the genetic control of phenotypic plasticity [52]. Moreover, it was suggested [53] that co-located effects are the result of the clustering of genes affecting phenotypic plasticity.

Three main strategies of drought resistance are recognized: drought escape, drought avoidance, and drought tolerance [54], and some authors recently proposed chlorophyll content as an important drought adaptive trait [23,55,56,57]. Our results indicate that we can classify QTLs based on their association with the three strategies. Most QTLs associated with drought avoidance and drought tolerance strategies showed neutral or light positive effects on productivity traits. Correlation analysis between traits showed that there is a trade-off between developmental periods from DP-H and DH-M, suggesting the strong interactions of these two phenology-related traits with most of the other traits. Furthermore, four drought-escape QTLs showed significant effects on drought plasticity I (dtraits) while no effects were found on the corresponding ddftraits for drought plasticity II. All these four QTLs have positively affected yield. This suggests that drought escape strategy plays a central role in wheat genetic adaptation to water stress especially in the Mediterranean region [58].

### 3.4. Candidate Genes within QTL Intervals

When a full genome sequence is available, high-density genetic maps can provide sufficient accuracy and resolution for the identification of CGs underlying the QTLs [59,60,61]. For example, genes involved in flowering pathways in cereals were proposed as CGs for QTLs associated with complex traits [62]. In the current study, eight genes regulating flowering time were localized within 6 out of 13 QTL intervals affecting phenology. Although we did not identify major photoperiodic wheat *Ppd* genes within these intervals, *Ppd-A1* [63] was found within a QTL interval on Chr. 2A affecting all biomass related traits as well as GY and KNSP after adjustment for flowering time. Furthermore, the *TaGI*, which is known to be associated with circadian clock regulation of photoperiodic response in wheat [64], was localized together with *TaFT2-A* within the 3A.2 QTL interval for DP-H. The vernalization gene *Vrn-A1* was localized within the interval of QTL 5A.5 affecting DP-H. Genes of FT family, which are involved in the regulation of flowering, development, and plant adaptation [65], were localized within 4 QTL intervals, affected phenological traits.

The crosstalk of plant hormones is involved in plant development and its response to abiotic stresses [66,67]. GA biosynthesis genes and signaling related genes are dispersed along several wheat chromosomes [68] and some of them were proposed to be involved in response to abiotic stresses [16,67,69]. Our suggested GA-CGs agree with the known function of *GA2ox* genes in response to drought [67], and with our previous results that showed differential expression of *GA2ox3* in the roots of a drought-resistant WEW accession, as compared with susceptible WEW drought associated with [16]. *Ga20ox* and GASA family genes are also known to be involved in growth promotion under stress conditions [66,67], while *GA13ox* genes were not reported previously as regulators of response to abiotic stresses. Ethylene response factors are also involved in the regulation of plant growth [70] and stress responses [71]. Interestingly, these ethylene CGs were identified within seven plastic to drought QTLs that highlight the importance of this gene family for drought resistance in wheat.

### 3.5. Vrn-B3 Is a Candidate Gene for Major Drought Escape QTL

The 7B.1 QTL with the highest effect on DP-H explained 37% of the variation in flowering time. The WEW parent allele of 7B.1, associated with earlier heading in both WW and WL, prolongation of the maturity period, and increasing yield-related traits in WL, appeared to have strong effects on the plasticity of yield-related traits. The *Vrn-B3* (*TaFT1*), known to affect flowering in wheat [72] and found to reside within this region (together with other 25 genes), seems to be a CG for these strong effects. This gene is homologous to the *FLOWERING LOCUS T* (*FT*) gene of Arabidopsis that plays a central role in the control of the transition from vegetative to reproductive phase in flowering plants [73]. In barley (*Hordeum vulgare*), earlier flowering was associated with an increase of *HvFT1* copy number or with haplotype differences in the promoter region and first intron of this gene [74]. Orthologs of *FT* in different plant species were associated with the regulation of flowering in response to abiotic stresses [75,76]; however, *TaFT1* was not reported as a flowering regulator in response to drought in wheat previously.

## 4. Materials and Methods

### 4.1. Plant Material and Growth Conditions

The G×L RIL population (150 F_6_ lines) was derived from a cross between durum wheat LDN and WEW (accession G18-16), developed by single-seed descent [10]. The continuous water-deficient experiment was conducted in an insect-proof screen-house protected by a polyethylene top, at the experimental farm of the Hebrew University of Jerusalem in Rehovot, Israel (34°47′ N, 31°54′ E; 54 m above sea level). Two irrigation regimes were applied: WW, 750 mm control and WL, 350 mm, irrigated with a drip water system. A split-plot factorial (RIL × irrigation regime) block design with three replicates was employed; each block consisted of two main plots (for the two irrigation regimes), with main plots split into subplots as described in [10].

### 4.2. DNA Extraction and SNP Genotyping

DNA was extracted from fresh leaf tissue of the parental genotypes (LDN and G18-16) and a pooled sample of each of the 150 F_6_ RILs following a standard CTAB protocol [77]. DNA concentration was normalized to 50 ng/µL. SNP genotyping was performed using the Illumina Infinium 15 K Wheat platform, developed by TraitGenetics, Gatersleben, Germany [78], consisting of 12,905 SNPs selected from the wheat 90 K array [79].

### 4.3. Phenotypic Traits

Four sets of phenotypic traits were used in the present QTL analysis. One set of observed traits (e.g., measured in the field or the lab), and three sets of derivative traits: traits that were adjusted for phenology, or drought plasticity traits I and II (with and without adjustment for phenology). The observed set included 17 traits of which 13 were previously measured in the population under WL and WW conditions [10,43]: grain yield (GY); thousand kernel weight (TKW); kernel number per spike (KNSP); harvest index (HI); spike dry matter (SpDM); total dry matter (TotDM); carbon isotope ratio (δ^13^C); osmotic potential (OP); chlorophyll content (Chl); flag leaf rolling (LR); culm length (CL); days from planting to heading (DP-H); days from heading to maturity (DH-M). The four additional traits that were not analyzed earlier included: (i) vegetative dry matter (VegDM), comprised of stems and leaves, weighed after drying at 80 °C for 48 h; (ii) spike length (SpL) (cm) measured from the base of the spike to the start of awns at maturity stage; (iii and iv) flag leaf length (FLL) (cm) and flag leaf width (FLW) (mm), of the longest and widest parts of the flag leaf, respectively. Three representative plants were measured in each plot for each trait.

**Figure 4:**
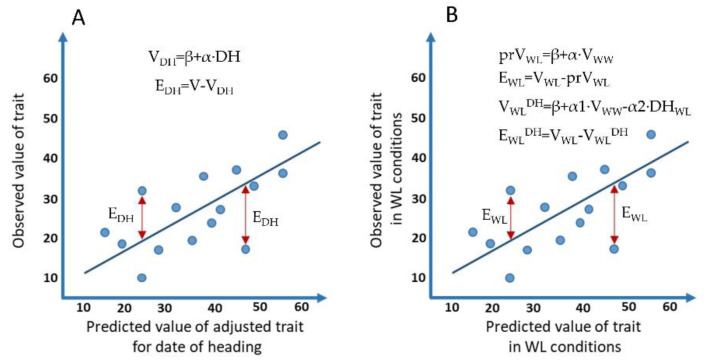
Graphical representation of the regression approach for the calculation of derivative traits. (**A**) Linear regression was calculated between the means of corresponding observed traits and DH to get predicted values of the trait (V_DH_). Obtained residuals between observed and predicted values of traits were used as “adjusted phenology traits” for each environment separately. (**B**) Linear regression was calculated between means of the observed trait values in the WW and WL conditions (V_WL_) and linear regression between means of the corresponding observed trait values in the WL treatment and trait values in the WW conditions and DP-H valuesin the dry treatment (V_WL_^DH^). Obtained residuals were used as “drought plasticity traits I” and “drought plasticity traits II”, respectively.

The three derivative sets of traits that were used for QTL mapping were obtained by calculating the deviations (residuals) from the regression line (Figure 4).

(1) The first derivative set defined here as ‘adjusted phenology traits’ was obtained, for each environment separately, by calculating the residuals of the linear regression between the means of the corresponding observed trait values and DP-H values (Figure 1A) to exclude the effect of differences in flowering phenology on these traits (prefix ’df‘ was added to the observed trait name):

V_DH_ = β + α∙DH, (1)

Ε_DH_ = V − V_DH,_ (2)

where V is a value of the observed trait, DH is DP-H value, V_DH_ is a predicted value of the trait based on linear regression, and Ε_DH_ is a residual from the regression line.

(2) The second derivative set defined here as ‘drought plasticity traits I’ (Figure 1B) was obtained by calculating the residuals of the linear regression between means of the observed trait values in the WW and WL conditions (prefix ’d‘ was added to observed trait name), to get a deviation between trait value in WL stress and WW condition, adjusted for the differences in trait values in the population under normal conditions:

prV_WL_ = β + α∙V_WW_, (3)

Ε_WL_ = V_WL_ − prV_WL,_ (4)

where V_WW_ is a value of the observed trait under WW conditions, V_WL_ is a value of the observed trait under WL conditions, prV_WL_ is a predicted value of the trait based on the linear regression, and E_WL_ is a residual from the regression line.

(3) The third derivative set defined here as ‘drought plasticity traits II’ was obtained by calculating the residuals of the linear regression between means of the corresponding observed trait values in the WL treatment and trait values in the WW conditions and DP-H values in the WL (prefix ‘ddf’ was added to the observed trait name), to exclude the effect of drought escape mechanisms in ‘drought plasticity traits I’ by taking into account the effect of heading time:

V_WL_^DH^ = β + α1∙V_WW_ − α2∙DH_WL_, (5)

E_WL_^DH^ = V_WL_ − V_WL_^DH^, (6)

where V_WW_ is a value of the observed trait under WW conditions, V_WL_ is a value of the observed trait under WL conditions, DH_WL_ is a value of DP-H under WL conditions, V_WL_^DH^ is a predicted value of the trait based on the linear regression, and E_WL_^DH^ is a residual from the regression line.

### 4.4. Statistical Analysis of Phenotypic Data

The JMP statistical package, version 11.0 (SAS Institute, Cary, NC, USA) was used for correlation and regression analyses. The correlation network analysis was conducted with the Software JASP 0.9 (JASP Team, Amsterdam, The Netherlands). All phenotypic values of observed and derivative traits were tested for normal distribution. The ANOVA was performed as a factorial model, with the irrigation regimes as fixed effects and genotypes and blocks as random effects. Heritability (*h*^2^) was calculated for each trait across the two irrigation treatments using variance components of ANOVA:

*h*^2^ = (σ_g_^2^)/((σ_g_^2^ + (σ_g×e_^2^)/e)), (7)

where:

σ_g_^2^ = [(MS_gen_ − MS_gen×e_)/e], (8)

σ_g×e_^2^ = MS_gen__×e_ (9)

e is the number of environments and MS is the mean square. The correlation values and the descriptive statistics were calculated on the mean values of phenotypic data for each observed trait and corresponding derivative traits.

### 4.5. Construction of the High-Density Genetic Map

The genetic map was constructed using MultiPoint software, section «UltraDense» (http://www.multiqtl.com, accessed on 21 August 2017) [80]. After filtering for missing data (removing markers with more than 10% missing data points) and large segregation distortion (χ^2^ > 35), the function “bound together” was applied to select the best candidate skeleton markers representing groups of co-segregating markers with a size of ≥2. Clustering of candidate markers into LG was performed at the threshold of recombination fraction RF = 0.2. The next step included marker ordering and testing of the local map stability and monotonicity for each LG [81,82]. Reducing the final number of LGs to 14, corresponding to haploid number chromosomes of tetraploid wheat, was performed by merging the LGs with minimum pairwise RF values expressed by their end markers (end-to-end association). Orientation of each LG concerning the short (S) and long (L) chromosome arms was performed according to the correspondence of the mapped markers with those on the consensus maps of hexaploid [79] and tetraploid wheat [83].

### 4.6. QTL Analysis

QTL analysis was performed using the general interval mapping (IM) procedure of MultiQTL 4.6 software package (http://www.multiqtl.com, accessed on 21 August 2017). First, single-QTL and two-linked-QTL models were used to screen the genetic linkage for each trait in each environment separately [82]. MEA was performed by a joint analysis of trait values scored in two environments (WL and WW). After separate analysis for each chromosome, multiple interval mapping (MIM) was used for reducing the residual variation for each QTL under consideration, by considering QTLs that reside on other chromosomes [84]. The significance of the detected QTL effects was tested using 5000 permutation runs. Significant models were further analyzed by 5000 bootstrap runs to estimate standard deviations of the chromosomal positions and QTL effects. Overlapping QTL effects (when a detected QTL affects two or more separate traits, were referred to as multi-trait QTLs. The software MapChart 2.2 was used for visualization of the QTL map [85].

### 4.7. Identification of Physical Position of the Mapped SNP Markers and CGs Residing within QTL Intervals

The physical positions of SNP markers were obtained by BLAST search of sequences of probes [79] against the whole-genome assembly of WEW accession ‘Zavitan’ [42]. A list of genes residing within each QTL interval (1.5 LOD support interval of QTL effect with highest LOD) was obtained from the annotated gene models of the ‘Zavitan’ genome assembly [42]. The putative genetic positions of the potential CGs on the QTL map were calculated based on a local linear approximation of genetic distances using the known physical positions of CGs relative to near markers.

## 5. Conclusions

Global climate changes require a better understanding of the genetic basis of crop plasticity in response to drought and other abiotic stresses. Here, we propose a new approach for quantification of the plasticity of complex traits measured under contrasting environmental conditions, which can be utilized in classical QTL and genome-wide association study (GWAS) analyses of plant response to a wide range of biotic and abiotic stresses. Furthermore, the application of the simultaneous adjustment of drought plasticity and phenological differences in the mapping population can improve the accuracy of QTL mapping and reveal hidden plasticity QTLs. The identification of CGs within QTL intervals may lead to the discovery of new pleiotropic effects of these genes by their interactions with additional networks that affect not only developmental processes but also plant response to environmental stresses. For example, *Vrn-B3* (*TaFT1*), which is proposed here as an important CG underlying a major drought plasticity QTL, possibly responsible for accelerated development of plants and significant improvement of yield under WL conditions by the drought escape strategy. Also, the higher phenotypic plasticity of the WEW parental line confirms the importance of crop wild relatives gene pool for the improvement of crop adaptability to environmental stresses.

## Figures and Tables

**Table 1:** Summary of quantitative trait loci (QTL) effects associated with yield, biomass, and phenology related, morphological, and drought adaptive physiological, traits, under water-limited (WL) and well-watered (WW) conditions.

Trait	# QTLs			LOD	ITV Allele		Environmental Specificity		Relation to Phenology			Relation to Drought	
	Total	Multi-trait	Single-trait		G18-16	LDN	WL	WW	Associated	Non-Plastic	Plastic	Non-Plastic	Plastic
Yield related traits													
GY	8	8	0	2.1–11.2	2	6	1	0	2	5	1	7	1
TKW	16	9	7	2.3–9.2	9	7	4	2	4	8	4	12	4
KNSP	11	10	1	2.3–8.9	2	9	1	0	4	4	3	9	2
HI	13	10	3	2.2–23.9	7	6	2	1	4	7	2	7	6
Biomass related traits													
SpDM	10	10	0	2.0–13.9	4	6	0	0	3	5	2	6	4
VegDM	6	5	1	2.2–4.4	1	4	0	0	0	5	0	1	4
TotDM	6	6	0	2.0–5.4	1	5	1	0	1	5	0	5	1
Morphological traits													
CL	15	12	3	2.5–9.5	4	11	0	1	6	7	2	10	5
SpL	10	10	0	2.0–10.8	6	4	1	1	2	7	1	7	3
FLL	9	7	2	2.0–7.7	3	6	0	0	4	5	0	6	3
FLW	17	13	4	2.1–32.4	8	9	0	2	5	9	3	14	3
Drought adaptive physiological traits													
δ^13^C	8	6	2	2.2–9.3	7	1	1	0	1	7	0	4	4
OP	9	7	2	2.3–4.9	1	8	0	3	0	7	1	7	2
Chl	13	10	3	2.0–7.0	7	6	0	2	1	7	5	11	2
LR	5	3	2	2.1–5.9	3	2	0	0	1	4	0	3	2
Phenology related traits													
DP-H	10	8	2	2.4–40.8	3	7	0	0	10	0	0	5	5
DP-M	8	8	0	3.0–13.6	4	4	1	0	5	3	0	6	2
All	78	44	34	2.0–40.8	--	--	--	--	15	41	21	46	33

**Table 2:** Number of QTL effects with increased trait value (ITV) alleles of drought plasticity QTL effects, conferred by the G18-16 and LDN.

Trait	ITV Allele of LDN		ITV Allele of G18-16	
	Number of QTLs	Total PEV *	Number of QTLs	Total PEV *
GY	0	0	1	0.21
TKW	1	0.13	3	0.37
KNSP	0	0	2	0.26
HI	3	0.33	3	0.39
SpDM	2	0.22	2	0.29
VegDM	3	0.31	1	0.13
TotDM	1	0.14	0	0
CL	4	0.46	1	0.11
SpL	2	0.17	1	0.17
FLL	2	0.21	1	0.14
FLW	1	0.12	2	0.21
δ^13^C	0	0	4	0.53
OP **	2	0.30	0	0
Chl	1	0.09	1	0.09
LR	2	0.24	0	0
DH-M	1	0.12	2	0.32

* Total PEV was calculated as a sum of PEV of effects of dtraits and ddftraits. When effects were co-located, PEV of higher effect was used for sum. ** Higher value of OP was associated with susceptibility to drought, allele contributed more negative values were associated with adaptivity.

**Table 3:** Number of QTL effects with ITV alleles of drought plasticity QTL effects, conferred by the G18-16 or LDN.

Drought Strategy	# QTLs	# QTLs Plastic to Drought	Allele Responsible for Drought Resistance		Effect on Productivity ***		
			G18-16	LDN	−	0	+
Escape	4	4	2 (2)	2 (2)	0	0	4
Avoidance	13	5	10 (4)	3 (1)	2	9	2
Tolerance	22	8	15 (5) *	7 (3)	1	13	8
Total	33 **	17	26 (11)	11 (6)	3	22	13

* For osmotic potential lower value reflects higher drought tolerance; ** The total number of QTLs presented in the column is lower than the sum of the number in the cells since some of the QTLs had pleiotropic effects on traits associated with different drought strategies; *** Positive effect (+) means the same ITV allele for the effects of the QTL on yield-related traits and physiological traits associated with drought resistance, while negative effect (**−**) means alternative ITV alleles; cases with no effect on productivity are denoted as ‘0′; The number of plastic QTLs for each allele responsible for drought resistance were shown in brackets.

## Data Availability

The data that support the findings of this study are available from the corresponding author upon reasonable request.

## Supplementary Materials

The following are available online at https://www.mdpi.com/1422-0067/22/4/1723/s1, Table S1: Summary of the genetic map constructed based on G×L RIL population, Table S2: Genetic and physical positions of mapped SNPs, Table S3: Number of RILs with parental (non-recombinant) chromosomes, Table S4: Rank correlations of genetic positions of the mapped markers with corresponding positions on other wheat genetic maps and physical positions and WEW genome assembly, Table S5: Normality test of observed and derivative traits, Table S6: Mean values and ranges of 17 phenotypic traits, Table S7: Analyses of variance (ANOVA) and heritability (*h*^2^), Table S8: Association between 17 observed traits in both treatments, Table S9: Associations between adjusted traits to time of heading in both treatments, Table S10: Association between plasticity traits to water stress with and without accounting of time of heading, Table S11: Association between corresponding traits in WW and WL and their variation between treatments, Table S12: Kendall’s tau coefficients of rank correlations between observed and derivative traits, Table S13: Parameters of QTL effects, Table S14: Summary of QTLs, Table S15: List of HC genes within QTL intervals, Table S16: Number of HC genes and physical intervals of QTLs, Table S17: Summary of CGs, Figure S1: Distribution of attached markers along 14 wheat chromosomes, Figure S2: Segregation distortion for each chromosome in the G×L RIL population, Figure S3 Frequency distribution of the 150 F6 RILs for 17 observed phenotypic traits, Figure S4: Frequency distribution of the 150 F6 RILs for 16 adjusted for heading date traits, Figure S5: Frequency distribution of the 150 F6 RILs for 33 drought plasticity traits, Figures S6–S19: LOD score plot with putative genetic positions of CG and 1.5 LOD support intervals of QTL effects for 14 chromosomes.

## Abbreviations

WEW	wild emmer wheat
QTL	quantitative trait loci
DAT	drought adaptive traits
MEA	multi-environmental approach
DRI	drought-resistant index
RIL	recombinant inbred line
CG	candidate gene
SNP	single nucleotide polymorphism
LG	linkage group
cM	centimorgan
Chr.	chromosome
WW	well-watered
WL	water-limited
GY	grain yield
TKW	thousand kernel weight
KNSP	kernel number per spike
HI	harvest index
SpDM	spike dry matter
VegDM	vegetative dry matter
TotDM	total dry matter
CL	culm length
SpL	spike length
FLL	flag leaf length
FLW	flag leaf width
δ13C	carbon isotope ratio
OP	osmotic potential
Chl	chlorophyll content
LR	flag leaf rolling
DP-H	days from planting to heading
DH-M	days from heading to maturity
ANOVA	analysis of variance
dtraits	drought plasticity traits I
ddftraits	drought plasticity traits II
dftraits	traits adjusted for the effect of phenology
G18-16	wild emmer wheat accession G18-16
LDN	cv. Langdon
LOD	logarithm of the odds
ITV allele	increased trait value allele
PEV	proportion of explained variation
MIM	multiple interval mapping
GWAS	genome-wide association study
