# Supplementary material for: Genomic Architecture of Phenotypic Plasticity in Response to Water Stress in Tetraploid Wheat
Source: Int J Mol Sci. 2021 Feb 9;22(4):1723. doi: 10.3390/ijms22041723 (PMC7915520; doi:10.3390/ijms22041723)
Supplement: Supplementary file 1 [file ijms-22-01723-s001.zip › drought_ijms_SM1_rev.pdf]

Article title: Genomic architecture of phenotypic plasticity in response to water stress in tetraploid wheat.

Authors: Andrii Fatiukha[1,2], Mathieu Deblieck[3], Valentyna Klymiuk[1,2], Lianne Merchuk-Ovnat[4], Zvi Peleg[4], Frank Ordon[3], Tzion Fahima[1,2], Abraham Korol[1,2], Yehoshua Saranga[4], Tamar Krugman[1].

Table S1 Summary of the genetic map constructed based on G×L RIL population.
Table S2 Genetic and physical positions of mapped SNPs.
Table S3 Number of RILs with parental (non-recombinant) chromosomes.
Table S4 Rank correlations of genetic positions of the mapped markers with corresponding positions on other wheat genetic maps and physical positions and WEW genome assembly.
Table S5 Normality test of observed and derivative traits.
Table S6 Mean values and ranges of 17 phenotypic traits.
Table S7 Analyses of variance (Anova) and heritability (H^2).
Table S8 Association between 17 observed traits in both treatments.
Table S9 Associations between adjusted traits to time of heading in both treatments.
Table S10 Association between plasticity traits to water stress with and without accounting of time of heading.
Table S11 Association between corresponding traits in WW and WL and their variation between treatments.
Table S12 Kendall's tau coefficients of rank correlations between observed and derivative traits.
Table S13 Parameters of QTL effects.
Table S14 Summary of QTLs.
Table S15 List of HC genes within QTL intervals.
Table S16 Number of HC genes and physical intervals of QTLs.
Table S17 Summary of CGs.
Fig. S1 Distribution of attached markers along 14 wheat chromosomes.
Fig. S2 Segregation distortion for each chromosome in the G×L RIL population.
Fig. S3 Frequency distribution of the 150 F6 RILs for 17 observed phenotypic traits.
Fig. S4 Frequency distribution of the 150 F6 RILs for 16 adjusted for heading date traits.
Fig. S5 Frequency distribution of the 150 F6 RILs for 33 drought plasticity traits.Fig. S6 LOD

score plot with putative genetic positions of CG and 1.5 LOD support intervals of QTL effects for chromosome 1A.

Fig. S7 LOD score plot with putative genetic positions of CG and 1.5 LOD support intervals of QTL effects for chromosome 1B.

Fig. S8 LOD score plot with putative genetic positions of CG and 1.5 LOD support intervals of QTL effects for chromosome 2A.

Fig. S9 LOD score plot with putative genetic positions of CG and 1.5 LOD support intervals of QTL effects for chromosome 2B.

Fig. S10 LOD score plot with putative genetic positions of CG and 1.5 LOD support intervals of QTL effects for chromosome 3A.

Fig. S11 LOD score plot with putative genetic positions of CG and 1.5 LOD support intervals of QTL effects for chromosome 3B.

Fig. S12 LOD score plot with putative genetic positions of CG and 1.5 LOD support intervals of QTL effects for chromosome 4A.

Fig. S13 LOD score plot with putative genetic positions of CG and 1.5 LOD support intervals of QTL effects for chromosome 4B.

Fig. S14 LOD score plot with putative genetic positions of CG and 1.5 LOD support intervals of QTL effects for chromosome 5A.

Fig. S15 LOD score plot with putative genetic positions of CG and 1.5 LOD support intervals of QTL effects for chromosome 5B.

Fig. S16 LOD score plot with putative genetic positions of CG and 1.5 LOD support intervals of QTL effects for chromosome 6A.

Fig. S17 LOD score plot with putative genetic positions of CG and 1.5 LOD support intervals of QTL effects for chromosome 6B.

Fig. S18 LOD score plot with putative genetic positions of CG and 1.5 LOD support intervals of QTL effects for chromosome 7A.

Fig. S19 LOD score plot with putative genetic positions of CG and 1.5 LOD support intervals of QTL effects for chromosome 7B.

Table S1 Summary of the genetic map constructed based on G×L RIL population

Linkage group	Total markers	Skeletal markers	Co-segregated markers	Length (cM)	Average interval (cM)/locus
1A	267	89	178	123.0	1.38
1B	325	114	211	112.9	0.99
2A	242	90	152	159.3	1.77
2B	446	134	312	147.0	1.10
3A	256	84	172	138.0	1.64
3B	351	117	234	136.9	1.17
4A	184	72	112	127.8	1.78
4B	121	51	70	84.6	1.66
5A	300	100	200	157.0	1.57
5B	393	146	247	165.3	1.13
6A	278	85	193	107.3	1.26
6B	336	90	246	107.5	1.19
7A	257	96	161	140.7	1.47
7B	259	101	158	128.4	1.27
Group 1	592	203	389	235.9	1.16
Group 2	688	224	464	306.3	1.37
Group 3	607	201	406	274.9	1.37
Group 4	305	123	182	212.4	1.73
Group 5	693	246	447	322.3	1.31
Group 6	614	175	439	214.8	1.23
Group 7	516	197	319	269.1	1.37
A genome	1784	617	1235	953.1	1.54
B genome	2231	753	1513	882.6	1.17
**Total**	**4015**	**1369**	**2646**	**1835.7**	**1.34**

Table S3 Number of RILs with non-recombinant chromosomes.

Chromosome	Langdon	Gitit (G18-16)	Total	% RIL
1A	3	4	7	4.6
1B	1	12	13	8.6
2A	2	1	3	2.0
2B	2	10	12	7.9
3A	3	7	10	6.6
3B	6	1	7	4.6
4A	7	3	10	6.6
4B	0	26	26	17.2
5A	4	2	6	4.0
5B	5	0	5	3.3
6A	3	8	11	7.3
6B	6	10	16	10.6
7A	2	2	4	2.6
7B	4	1	5	3.3
Genome A	24	27	51	4.8
Genome B	24	60	84	7.9
**Total**	**48**	**87**	**135**	**6.3**

Table S4 Rank correlations of genetic positions of the mapped markers with corresponding positions on other wheat genetic maps and physical positions and WEW genome assembly.

Chromosome	Svevo×Zavitan	Consensus tetraploid	Consensus hexaploid	Reference genome
1A	0.99	0.99	0.98	0.99
1B	1.00	1.00	0.99	1.00
2A	0.97	1.00	0.93	1.00
2B	1.00	1.00	1.00	1.00
3A	1.00	1.00	0.98	1.00
3B	1.00	1.00	0.96	1.00
4A	1.00	1.00	1.00	1.00
4B	0.99	0.99	0.97	0.98
5A	1.00	0.99	0.99	0.99
5B	1.00	1.00	1.00	1.00
6A	1.00	0.97	0.99	0.99
6B	1.00	1.00	0.99	1.00
7A	1.00	1.00	0.99	1.00
7B	1.00	1.00	1.00	1.00
**Average**	**1.00**	**1.00**	**0.98**	**1.00**

Table S6 Mean values and ranges of 17 phenotypic traits.

	GY	VegDM	SpDM	TotDM	HI	TKW	δ13C	KNSP	DP-H	DH-M	CL	SpL	FLW	FLL	LR	Chl	OP
**Observed traits**																	
Mean																	
WL	56.2	162.5	119.5	282.0	0.425	42.4	-24.8	39.6	95.0	35.7	142.9	24.2	1.6	27.1	1.8	54.5	-13.5
WW	27.8	111.7	70.8	182.2	0.389	30.4	-23.0	32.2	91.3	32.5	116.8	23.7	1.4	20.2	2.1	59.3	-16.1
Range																	
WL	16.3–93.5	98.3–237.5	65.3–183.3	179.2–417.2	0.269–0.50	17.4–60.6	-26.6—23.5	14.1–69.8	83.0–107.0	27.3–41.0	101.0–174.2	19.0–47.3	1.1–2.4	18.1–35.3	1.0–3.0	47.6–64.3	-9.7—16.5
WW	4.9–59.3	70.9–167.8	44.7–106.7	134.7–254.3	0.298–0.523	14.8–45.4	-24.0—21.9	5.4–52.6	78.5–110.7	17.0–39.7	91.3–151.7	18.2–32.2	1.0–2.1	13.2–31.1	1.0–3.0	44.4–68.4	-11.9—19.3
**Adjusted to heading date traits**																	
Range																	
WL	-27.1–28.2	-35.4–52.1	-28–26.7	-45.4–73.9	-0.077–0.076	-14.7–13.6	-0.98–0.86	-19.4–20.2	-//-	-6.16–5.66	-36.7–30.5	-4.8–7.5	-0.34–0.73	-6.10–6.20	-1.21–1.15	-13.5–9.2	-3.1–4.2
WW	-36.1–36.7	-62.1–75.5	-41.4–57.4	-96.4–132.8	-0.101–0.095	-25–18.1	-1.82–1.37	-24.3–30.7	-//-	-6.75–5.76	-46.7–35.1	-5.4–22.8	-0.49–0.75	-8.95–8.58	-0.92–1.34	-7.0–7.6	-3.1–3.8
**Drought plasticity traits I**																	
Range																	
	-24.5–25.8	-26.3–34.7	-26.3–34.7	-47.1–77.2	-0.073–0.115	-17.1–13.7	-1.12–1.19	-21.3–14.8	-7.4–11.5	-14.93–6.29	-25.3–29.0	-9.0–7.2	-0.26–0.55	-7.40–11.12	-1.15–1.15	-13.1–9.7	-2.7–3.8
**Drought plasticity traits**																	
Range																	
	-30.1–22.1	-30.9–26.7	-30.9–26.7	-47.6–75.8	-0.074–0.085	-16.2–12.8	-0.93–0.93	-16.6–15.5	-//-	-5.89–5.39	-31.3–27.3	-8.7–6.2	-0.30–0.53	-6.43–6.65	-1.22–1.24	-12.5–10.4	-2.6–3.9

Table S7 Analyses of variance (Anova) and heritability (H^2) for 17 observed phenotypic traits in 150 RILs.

	GY	VegDM	SpDM	TotDM	HI	TKW	δ13C	KNSP	DP-H	DH-M	CL	SpL	FLW	FLL	LR	Chl	OP
Mean square																	
Genotype	1898.4 ***	1875.2 **	1205.4 ***	4456.4 **	0.0065 ***	130.58 ***	0.860 ***	305.4 ***	159.54 ***	48.82 ***	738.7 ***	28.97 ***	0.2094 ***	42.25 ***	1.151 ***	46.08 ***	5.51 ***
Irrigation	672286.3 ***	517347.4 ***	513293.5 ***	2005144.5 ***	0.27 ***	31296.3 ***	697.7 ***	13418.7 ***	2822.29 ***	2265.7 ***	155636.1 ***	48.41 *	11.88 ***	10071.6 *	17.37 ***	4874.3 ***	1377.3 ***
G × I	711.6 n.s.	1044.8 n.s.	571.2 n.s.	2463.6 n.s.	0.0023 n.s.	66.9 n.s.	0.406 n.s.	83.29 *	15.15 n.s.	25.33 ***	186.1 n.s.	8.50 n.s.	0.0415 n.s.	19.55 n.s.	0.457 n.s.	17.95 n.s.	3.27 n.s.
Block	1137.3 *	2714.6 n.s.	1117.6 n.s.	7281.3 n.s.	0.0051 *	1081.2 n.s.	10.38 ***	22.38 n.s.	637.3 ***	384.7 ***	11774.7 ***	3.39 n.s.	1.04 ***	194.1 n.s.	1.494 *	76.93 n.s.	19.46 ***
Heritability (h^2)	0.63	0.44	0.53	0.45	0.65	0.49	0.53	0.73	0.91	0.48	0.75	0.71	0.80	0.54	0.60	0.61	0.41

*, **, *** and n.s. indicate significance at P ≤ 0.05, 0.01, 0.001 or non-significant effect, respectively.

Table S8 Association between 17 observed traits in both treatments. Coefficients of correlation (r) between 17 observed traits in both treatments (WL and WW). Significant correlation coefficients are marked red text. Positive correlations are marked with yellow and negative are marked with blue.

		WL																
		GY	KNSp	TKW	HI	SpDM	VegDM	TotDM	CL	SpL	FLW	FLL	LR	Chl	OP	δ13C	DP-H	DH-M
WW	GY	1.00	0.60	0.55	0.52	0.84	0.21	0.53	0.47	-0.19	0.07	-0.18	-0.08	0.13	-0.09	-0.11	-0.55	0.58
	KNSP	0.62	1.00	0.06	0.28	0.50	0.14	0.33	0.37	-0.07	0.18	-0.11	-0.06	0.15	-0.08	-0.03	-0.35	0.39
	TKW	0.52	0.19	1.00	0.25	0.44	0.13	0.30	0.29	0.03	-0.03	0.01	-0.01	0.05	-0.08	-0.04	-0.30	0.39
	HI	0.56	0.33	0.22	1.00	0.52	-0.55	-0.14	0.39	-0.20	-0.09	-0.35	0.09	0.11	-0.12	-0.35	-0.71	0.63
	SpDM	0.82	0.49	0.35	0.53	1.00	0.38	0.73	0.50	-0.01	-0.02	-0.11	0.04	0.04	0.05	-0.17	-0.56	0.54
	VegDM	0.32	0.23	0.21	-0.38	0.54	1.00	0.89	0.05	0.23	0.06	0.26	-0.03	-0.08	0.18	0.20	0.24	-0.16
	TotDM	0.61	0.39	0.30	0.01	0.83	0.91	1.00	0.26	0.19	0.03	0.14	0.01	-0.04	0.16	0.04	-0.08	0.14
	CL	0.17	0.22	0.10	0.04	0.25	0.29	0.31	1.00	-0.05	0.04	-0.20	0.10	-0.03	-0.10	0.11	-0.58	0.58
	SpL	-0.14	-0.08	-0.01	-0.09	0.01	0.10	0.07	0.15	1.00	0.04	0.33	0.03	-0.17	0.13	0.12	0.24	-0.25
	FLW	0.16	0.32	0.12	-0.07	0.17	0.28	0.26	0.16	0.21	1.00	0.16	-0.24	0.33	-0.25	0.27	0.07	0.02
	FLL	-0.01	0.17	0.16	-0.20	0.05	0.27	0.22	0.29	0.27	0.37	1.00	0.08	-0.05	0.03	0.16	0.48	-0.41
	LR	-0.12	-0.12	-0.04	-0.05	0.00	0.05	0.03	0.01	0.02	-0.18	-0.03	1.00	-0.10	0.08	-0.02	-0.13	0.10
	Chl	0.27	0.20	0.23	0.11	0.23	0.14	0.20	-0.01	0.04	0.31	-0.04	0.03	1.00	-0.22	0.01	-0.11	0.13
	OP	-0.24	-0.17	-0.11	-0.21	-0.22	-0.05	-0.13	-0.17	0.08	-0.25	-0.07	0.05	-0.24	1.00	-0.14	0.14	-0.16
	δ13C	-0.02	-0.09	0.02	-0.11	0.10	0.25	0.21	0.26	0.12	0.12	0.01	0.11	0.05	-0.07	1.00	0.22	-0.16
	DP-H	-0.31	-0.06	-0.06	-0.47	-0.37	0.05	-0.16	-0.34	-0.06	0.00	0.09	-0.19	-0.21	0.30	-0.07	1.00	-0.93
	DH-M	0.35	0.07	0.41	0.21	0.30	0.10	0.22	0.26	0.08	-0.04	0.00	0.13	0.10	-0.07	-0.07	-0.46	1.00

Table S9 Associations between adjusted traits to time of heading in both treatments. Coefficients of correlation (r) between adjusted traits to time of heading in both treatments (WW and WL). Significant correlation coefficients are marked red text. Positive correlations are marked with yellow and negative are marked with blue.

		**Adjusted traits to time of heading (df traits) in WL**															
		**dfGY**	**dfKNSp**	**dfTKW**	**dfHI**	**dfSpDM**	**dfVegDM**	**dfTotDM**	**dfCL**	**dfSpL**	**dfFLW**	**dfFLL**	**dfLR**	**dfChl**	**dfOP**	**dfδ13C**	**dfDH-M**
**Adjusted traits to time of heading (df traits) in WW**	**dfGY**	1.00	0.52	0.49	0.23	0.77	0.42	0.58	0.21	-0.07	0.13	0.11	-0.19	0.09	-0.02	0.02	0.23
	**dfKNSp**	0.63	1.00	-0.05	0.06	0.39	0.25	0.32	0.22	0.02	0.22	0.07	-0.12	0.12	-0.04	0.05	0.18
	**dfTKW**	0.53	0.18	1.00	0.06	0.35	0.21	0.29	0.15	0.11	-0.01	0.18	-0.05	0.02	-0.04	0.03	0.32
	**dfHI**	0.49	0.34	0.21	1.00	0.20	-0.56	-0.29	-0.05	-0.04	-0.05	-0.01	-0.01	0.05	-0.03	-0.28	-0.14
	**dfSpDM**	0.79	0.51	0.36	0.44	1.00	0.63	0.83	0.26	0.16	0.02	0.22	-0.04	-0.03	0.15	-0.05	0.09
	**dfVegDM**	0.36	0.23	0.22	-0.40	0.60	1.00	0.94	0.24	0.19	0.05	0.17	0.01	-0.06	0.16	0.15	0.19
	**dfTotDM**	0.60	0.39	0.30	-0.07	0.84	0.93	1.00	0.26	0.22	0.04	0.20	0.00	-0.04	0.17	0.06	0.18
	**dfCL**	0.07	0.21	0.09	-0.14	0.14	0.32	0.28	1.00	0.12	0.10	0.11	0.03	-0.12	-0.03	0.30	0.11
	**dfSpL**	-0.17	-0.08	-0.01	-0.14	-0.01	0.10	0.07	0.13	1.00	0.02	0.25	0.07	-0.15	0.10	0.07	-0.07
	**dfFLW**	0.17	0.32	0.12	-0.08	0.19	0.28	0.27	0.17	0.21	1.00	0.15	-0.24	0.34	-0.26	0.26	0.23
	**dfFLL**	0.02	0.18	0.17	-0.17	0.09	0.27	0.24	0.34	0.28	0.37	1.00	0.17	0.00	-0.04	0.06	0.12
	**dfLR**	-0.19	-0.13	-0.05	-0.17	-0.07	0.06	0.00	-0.05	0.00	-0.18	-0.01	1.00	-0.12	0.10	0.01	-0.06
	**dfChl**	0.22	0.19	0.22	0.02	0.17	0.15	0.17	-0.09	0.03	0.32	-0.02	-0.01	1.00	-0.21	0.03	0.08
	**dfOP**	-0.16	-0.15	-0.10	-0.09	-0.12	-0.07	-0.09	-0.07	0.10	-0.26	-0.10	0.12	-0.19	1.00	-0.17	-0.09
	**dfδ13C**	-0.04	-0.10	0.01	-0.16	0.08	0.25	0.20	0.25	0.12	0.12	0.02	0.10	0.04	-0.05	1.00	0.12
	**dfDH-M**	0.25	0.04	0.43	-0.01	0.16	0.14	0.16	0.13	0.05	-0.05	0.05	0.05	0.00	0.08	-0.11	1.00

Table S10 Association between plasticity traits to water stress with and without accounting of time of heading. Coefficients of correlation (r) between plasticity traits to water stress with and without accounting of time of heading. Significant correlation coefficients are marked red text. Positive correlations are marked with yellow and negative are marked with blue.

		Traits of plasticity to water stress with accounting of time of heading (ddftraits)																
		ddfGY	ddfKNSp	ddfTKW	ddfHI	ddfSpDM	ddfVegDM	ddfTotDM	ddfCL	ddfSpL	ddfFLW	ddfFLL	ddfLR	ddfChl	ddfOP	ddfδ13C	ddfDP-H	ddfDH-M
Traits of plasticity to water stress (dtraits)	dGY	1.00	0.55	0.54	0.23	0.77	0.43	0.59	0.23	0.03	0.18	0.11	-0.12	0.06	0.02	0.01	--	0.20
	dKNSp	0.62	1.00	0.20	0.03	0.42	0.31	0.39	0.12	0.08	0.10	0.01	-0.05	-0.02	0.05	-0.07	--	0.16
	dTKW	0.60	0.29	1.00	0.11	0.35	0.18	0.26	0.14	0.11	0.07	0.14	-0.05	0.06	-0.04	-0.03	--	0.32
	dHI	0.41	0.22	0.23	1.00	0.19	-0.53	-0.27	-0.01	0.06	-0.02	0.02	0.04	0.05	-0.05	-0.24	--	-0.10
	dSpDM	0.82	0.51	0.43	0.38	1.00	0.65	0.83	0.26	0.18	0.08	0.20	-0.02	0.00	0.15	-0.05	--	0.08
	dVegDM	0.26	0.21	0.10	-0.54	0.45	1.00	0.95	0.18	0.11	0.06	0.15	-0.01	-0.05	0.18	0.12	--	0.14
	dTotDM	0.54	0.38	0.26	-0.20	0.75	0.91	1.00	0.21	0.17	0.08	0.18	-0.01	-0.03	0.18	0.04	--	0.13
	dCL	0.38	0.23	0.24	0.24	0.39	0.03	0.19	1.00	0.17	0.02	0.10	0.02	-0.02	-0.01	0.13	--	0.04
	dSpL	-0.11	-0.03	0.01	-0.11	0.00	0.18	0.14	-0.01	1.00	0.09	0.21	0.01	-0.05	-0.02	0.00	--	-0.04
	dFLW	0.06	0.04	0.02	-0.12	-0.03	0.09	0.07	-0.07	0.13	1.00	0.15	-0.10	0.25	-0.10	0.13	--	0.30
	dFLL	-0.13	-0.15	-0.01	-0.20	-0.07	0.25	0.14	-0.14	0.33	0.21	1.00	0.11	-0.01	-0.02	0.05	--	0.15
	dLR	-0.05	-0.02	-0.03	0.07	0.04	-0.03	-0.01	0.05	-0.01	-0.11	0.06	1.00	-0.01	0.00	0.07	--	-0.03
	dChl	0.06	-0.01	0.07	0.03	0.02	-0.05	-0.02	0.03	-0.06	0.24	-0.03	0.00	1.00	-0.17	0.04	--	0.11
	dOP	0.01	0.03	-0.05	-0.07	0.12	0.19	0.18	-0.05	-0.01	-0.09	0.01	-0.01	-0.17	1.00	-0.13	--	-0.10
	dδ13C	-0.11	-0.16	-0.10	-0.28	-0.16	0.18	0.03	-0.03	0.08	0.16	0.16	0.05	0.02	-0.11	1.00	--	0.10
	dDP-H	-0.14	-0.11	-0.10	-0.18	-0.15	0.12	0.02	-0.33	0.12	0.11	0.37	-0.12	-0.19	0.16	0.11	1.00	--
	dDH-M	0.46	0.34	0.39	0.45	0.41	-0.20	0.06	0.43	-0.27	-0.03	-0.34	0.05	0.15	-0.12	-0.24	-0.60	1.00

Table S11. Association between corresponding traits in WW and WL and their variation between treatments. Coefficients of correlation (R1) between values of traits under WW and WL conditions and coefficients of correlation (R2) between values of traits under WW and trait variation calculated as difference of values of trait under WL and WW. Significant correlation coefficients are marked red text (P≤0.05).

	R1	R2
**GY**	0.46	-0.81
**KNSP**	0.67	-0.49
**TKW**	0.29	-0.73
**HI**	0.56	-0.46
**SpDM**	0.35	-0.85
**VegDM**	0.21	-0.80
**TotDM**	0.19	-0.83
**CL**	0.54	-0.53
**SpL**	0.55	-0.65
**FLW**	0.69	-0.58
**FLL**	0.30	-0.49
**LR**	0.41	-0.49
**Chl**	0.41	-0.43
**OP**	0.18	-0.59
**δ13C**	0.31	-0.71
**DP-H**	0.85	0.16
**DH-M**	0.40	-0.14

Table S12 Kendall's tau coefficients of rank correlations between observed and derivative traits.

Traits	Adjusted to heading traits		Drought plasticity trats I*	Drought plasticity trats II*
	WW	WL		
**GY**	0.797	0.600	0.679	0.550
**KNSP**	0.951	0.755	0.560	0.500
**TKW**	0.960	0.790	0.807	0.747
**HI**	0.680	0.504	0.625	0.484
**SpDM**	0.778	0.622	0.776	0.624
**VegDM**	0.968	0.853	0.862	0.802
**TotDM**	0.907	0.952	0.884	0.883
**CL**	0.790	0.609	0.639	0.519
**SpL**	0.953	0.851	0.750	0.691
**FLW**	0.991	0.964	0.515	0.502
**FLL**	0.941	0.701	0.803	0.634
**LR**	0.895	0.924	0.810	0.784
**Chl**	0.862	0.937	0.772	0.771
**OP**	0.807	0.917	0.840	0.834
**δ13C**	0.956	0.863	0.811	0.765
**DP-H**	-//-	-//-	0.280	-//-
**DH-M**	0.704	0.185	0.720	0.187

*For calculation of correlations for drought plasticity traits only WL observed set was used.

Table S13 Parameters of QTL effects. Parameters of QTL effects for 17 observed traits, 16 traits adjusted to heading and 33 drought plasticity traits in G×L RIL population under two water regimes (WL – water limited and WW – well-watered).

QTL effects	QTL	LOD	Position (cM)	Interval (cM)	Length (cM)	Nearest marker	WL		WW		ITV Allele
							PEV	d	PEV	d	
**Yield related traits**											
**Grain yield**											
QdfGy.huj.uh-2A	2A.1	3.8	38.3	32.7—42.6	9.9	BobWhite_c10977_834	0.07	-3.85±1.40	0.07	-6.53±3.75	L
QGy.huj.uh-2B.1	2B.2	2.4	40.8	29.7—50.1	20.3	GENE-1741_103	0.13	3.10±1.96	0.15	4.13±3.38	G
QdfGy.huj.uh-2B.1	2B.2	2.1	39.4	29.8—52.8	23.0	Kukri_c62277_80	0.07	3.07±2.56	0.07	0.47±7.75	G
QGy.huj.uh-2B.2	2B.6	8.2	103.8	101.4—107.9	6.6	BobWhite_c47357_535	0.13	-4.12±1.56	0.15	-10.98±2.16	L
QGy.huj.uh-3B	3B.2	3.1	67.9	64.5—86.0	21.5	BobWhite_c17191_297	0.08	-4.91±1.86	0.08	-8.00±3.17	L
QdfGy.huj.uh-3B	3B.2	2.7	79.3	65.0—87.1	22.1	wsnp_Ex_c12781_20280445	0.08	-3.95±1.63	0.08	-7.60±3.21	L
QGy.huj.uh-4A	4A.6	4.1	104.0	96.2—110.9	14.7	wsnp_Ex_c3988_7221220	0.05	-3.85±1.75	0.12	-9.89±3.13	L
QdfGy.huj.uh-4A	4A.6	3.1	104.0	89.1—119.5	30.4	wsnp_Ex_c3988_7221220	0.06	-3.66±1.47	0.06	-6.53±2.94	L
QGy.huj.uh-4B	4B.3	6.1	35.0	28.7—43.0	14.3	RAC875_c1052_94	0.07	-4.81±1.72	0.11	-10.49±2.39	L
QdfGy.huj.uh-4B	4B.3	5.2	23.3	19.5—39.7	20.3	BS00022431_51	0.07	-3.98±1.66	0.14	-10.59±2.35	L
QGy.huj.uh-5A	5A.3	11.2	39.1	36.3—41.6	5.3	Ra_c69221_1167	0.03	-2.98±1.17	0.09	-9.01±1.22	L
QdfGy.huj.uh-5A	5A.3	3.4	39.1	33.1—52.8	19.7	Ra_c69221_1167	0.05	-2.38±2.52	0.08	-7.39±3.96	L
QGy.huj.uh-7B	7B.1	11.1	14.9	13.0—16.5	3.4	Tdurum_contig42423_1963	0.23	8.64±0.93	--	--	G
QdGy.huj.uh-7B	7B.1	6.3	14.9	12.8—18.9	6.2	Tdurum_contig42423_1963	0.21	7.25±1.07	--	--	G
**Thousand kernel weight**											
QdfTkw.huj.uh-1A	1A.3	4.7	94.3	88.9—102.6	13.7	Ra_c41164_730	0.10	3.19±0.42	--	--	G
QddfTkw.huj.uh-1A	1A.3	2.9	94.3	87.5—110.4	22.9	Ra_c41164_730	0.10	3.08±0.49	--	--	G
QTkw.huj.uh-1B	1B.2	5.3	18.0	10.3—22.3	10.0	CAP7_c3847_204	0.03	-1.63±0.96	0.11	-4.46±1.09	L
QdfTkw.huj.uh-1B	1B.2	3.1	18.0	0.1—34.12	34.0	GENE-1756_115	0.05	-1.84±1.18	0.12	-4.53±1.58	L
QTkw.huj.uh-2B.1	2B.1	3.3	27.4	19.2—36.0	16.8	IAAV3165	0.11	3.48±0.42	--	--	G
QdfTkw.huj.uh-2B.1	2B.1	5.9	25.2	23.4—29.3	5.9	RAC875_c17720_436	0.10	3.12±0.48	--	--	G
QddfTkw.huj.uh-2B.2	2B.4	3.7	83.1	60.5—98.2	37.7	IAAV5350	0.12	3.41±0.49	--	--	G
QTkw.huj.uh-2B.3	2B.7	2.3	117.7	110.4—127.5	17.1	Excalibur_c18966_1008	--	--	0.09	-3.61±1.87	L
QTkw.huj.uh-3A	3A.2	5.6	19.9	15.4—24.3	8.9	Excalibur_c34649_556	0.10	3.29±0.69	0.04	2.64±1.13	G
QTkw.huj.uh-4A.1	4A.3	9.2	44.7	40.6—50.2	9.7	wsnp_Ex_c24443_33688235	0.10	-3.24±0.72	0.13	-4.77±1.03	L
QdfTkw.huj.uh-4A.2	4A.4	6.6	56.3	35.9—64.0	28.1	IAAV4351	0.11	-3.30±0.83	0.08	-3.86±1.05	L
QddfTkw.huj.uh-4A.2	4A.4	3.2	60.8	44.9—74.6	29.7	RAC875_c48107_65	0.13	-3.32±1.04			L
QTkw.huj.uh-4B	4B.3	2.8	36.4	32.4—43.1	10.7	RAC875_c25710_297	0.10	-3.00±1.24	0.06	-2.82±1.75	L
QdfTkw.huj.uh-4B	4B.3	3.6	36.4	33.7—39.8	6.1	RAC875_c25710_297	0.07	-2.45±1.24	0.06	-3.03±1.40	L
QdfTkw.huj.uh-5A	5A.1	4.7	0.0	0.0—7.9	7.8	BobWhite_c7114_237	0.05	1.24±1.78	0.09	3.27±2.86	G
QTkw.huj.uh-5B	5B.1	5.2	43.6	37.8—46.5	8.7	BS00023081_51	0.06	2.36±0.74	0.09	4.09±0.81	G
QdfTkw.huj.uh-5B	5B.1	2.0	43.9	31.1—67.1	36.0	Excalibur_c8082_478	0.05	1.83±1.28	0.07	3.23±1.68	G
QTkw.huj.uh-6A	6A.3	3.5	55.3	46.5—63.3	16.8	Kukri_c14877_303	0.09	3.16±0.49	--	--	G

QTL effects	QTL	LOD	Position (cM)	Interval (cM)	Length (cM)	Nearest marker	WL		WW		ITV Allele
							PEV	d	PEV	d	
QdfTkw.huj.uh-6A	6A.3	2.4	53.5	45.0—62.3	17.3	Tdurum_contig76709_195	0.10	2.93±1.21	0.03	1.68±1.87	G
QTkw.huj.uh-6B	6B.3	2.9	41.5	36.7—49.8	13.0	TA002500-1001	--	--	0.08	-2.97±2.51	L
QdfTkw.huj.uh-6B	6B.3	2.3	41.5	28.2—48.9	20.7	TA002500-1001	0.03	-0.79±1.61	0.08	-2.49±2.90	L
QdfTkw.huj.uh-7A.1	7A.2	4.7	19.6	14.6—26.5	11.9	Excalibur_rep_c115261_135	0.03	-0.70±1.55	0.12	-4.11±2.62	L
QTkw.huj.uh-7A.2	7A.5	4.4	81.8	73.3—96.2	22.9	BS00049871_51	0.06	2.47±0.98	0.06	2.68±1.95	G
QTkw.huj.uh-7B	7B.1	5.7	14.91	11.2—31.5	20.35	Tdurum_contig42423_1963	0.15	4.18±0.52	--	--	G
QdTkw.huj.uh-7B	7B.1	3.9	14.9	10.8—24.1	13.3	Tdurum_contig42423_1963	0.15	3.84±0.62	--	--	G
**Kernel number per spike**											
QdfKnsp.huj.uh-1A	1A.3	3.2	91.7	86.4—101.8	15.4	Excalibur_c33470_175	0.05	-2.84±2.33	0.05	-3.38±2.31	L
QKnsp.huj.uh-2A	2A.1	3.3	36.4	32.5—38.9	6.3	wsnp_Ex_c2033_3814035	0.04	-2.67±2.09	0.07	-4.22±2.20	L
QdfKnsp.huj.uh-2A	2A.1	3.7	38.3	35.0—40.1	5.1	BobWhite_c10977_834	0.04	-2.76±1.84	0.06	-3.97±2.10	L
QKnsp.huj.uh-2B.1	2B.4	8.9	86.0	82.8—88.8	6.0	wsnp_Ex_c55735_58127324	0.20	-0.68±7.34	0.23	-1.38±8.46	L
QdfKnsp.huj.uh-2B.1	2B.4	7.5	86.0	82.8—89.3	6.5	wsnp_Ex_c55735_58127324	0.05	-3.35±1.20	0.11	-5.94±1.02	L
QKnsp.huj.uh-2B.2	2B.6	7.3	106.4	101.7—111.6	9.9	Tdurum_contig12879_1200	0.20	-2.49±7.19	0.23	-3.72±8.20	L
QdfKnsp.huj.uh-3A	3A.6	4.2	130.7	127.2—135.4	8.2	wsnp_Ex_rep_c66274_64426901	0.05	-2.85±2.28	0.06	-3.55±2.80	L
QKnsp.huj.uh-5A.1	5A.3	4.7	45.5	36.9—50.7	13.8	wsnp_Ex_rep_c70343_69286072	0.05	-3.36±1.45	0.12	-6.07±1.10	
QdfKnsp.huj.uh-5A.1	5A.3	6.9	45.5	42.7—50.2	7.4	wsnp_Ex_rep_c70343_69286072	0.06	-3.71±1.11	0.11	-5.93±1.11	L
QKnsp.huj.uh-5A.2	5A.7	3.6	136.6	130.1—144.0	13.9	wsnp_Ex_c12684_20157261	0.04	-2.83±1.67	0.12	-5.95±1.32	L
QKnsp.huj.uh-6A	6A.2	2.3	51.4	44.1—57.0	12.9	BS00063977_51	0.04	-2.02±2.47	0.09	-4.78±2.38	L
QdfKnsp.huj.uh-6A	6A.2	5.1	48.4	44.33—54.32	10.0	wsnp_Ex_c19770_28768859	0.05	-3.35±1.07	0.09	-5.36±0.97	L
QKnsp.huj.uh-7A	7A.6	3.1	92.2	79.0—98.6	19.6	BobWhite_rep_c49367_405	0.05	-3.31±1.47	0.13	-5.96±1.23	L
QKnsp.huj.uh-7B.1	7B.1	3.0	14.9	4.7—19.8	15.2	Tdurum_contig42423_1963	0.10	5.21±1.16	--	--	G
QdKnsp.huj.uh-7B.1	7B.1	2.8	14.9	8.0—20.9	12.8	Tdurum_contig42423_1963	0.13	4.18±0.76	--	--	G
QdfKnsp.huj.uh-7B.2	7B.4	7.0	123.4	120.4—128.2	7.8	CAP7_c3950_160	0.13	5.70±0.96	0.04	3.27±1.15	G
QddfKnsp.huj.uh-7B.2	7B.4	2.8	123.4	113.2—128.4	15.2	CAP7_c3950_160	0.13	3.98±0.86	--	--	G
**Harvest index**											
QddfHi.huj.uh-1A	1A.4	3.8	109.3	98.4—117.4	19.0	CAP12_c8163_118	0.10	0.02±0.00	--	--	G
QHi.huj.uh-2B.1	2B.2	2.8	34.0	29.8—38.9	9.0	RAC875_rep_c109207_706	0.08	0.01±0.01	0.17	0.03±0.01	G
QdfHi.huj.uh-2B.1	2B.2	2.9	39.4	30.1—50.0	19.9	Kukri_c62277_80	0.07	0.01±0.01	0.11	0.02±0.01	G
QHi.huj.uh-2B.2	2B.6	9.3	98.2	90.9—103.9	13.0	Excalibur_c64983_65	0.08	-0.02±0.01	0.17	-0.02±0.01	L
QdfHi.huj.uh-3B	3B.1	3.5	31.3	96.8—112.5	15.6	BS00022242_51	0.10	-0.02±0.00	--	--	L
QddfHi.huj.uh-3B	3B.1	3.9	31.3	13.7—39.3	25.6	BS00022242_51	0.10	-0.02±0.00	--	--	L
QHi.huj.uh-4A.1	4A.1	2.2	24.0	4.3—31.9	27.7	Kukri_rep_c101034_516	0.13	0.02±0.03	--	--	G
QHi.huj.uh-4A.2	4A.6	2.4	95.1	84.2—118.6	34.4	BS00072157_51	--	--	0.06	-0.01±0.02	L
QdfHi.huj.uh-4A.2	4A.6	3.1	94.0	86.0—116.5	30.5	RAC875_c17197_504	0.09	-0.01±0.01	0.04	-0.01±0.01	L
QddfHi.huj.uh-5A.1	5A.2	4.4	19.5	16.6—33.5	17.0	BS00031073_51	0.12	-0.02±0.01	--	--	L
QHi.huj.uh-5A.2	5A.3	7.8	41.9	38.3—44.0	5.8	wsnp_Ra_c14112_22155312	0.08	-0.02±0.01	0.07	-0.02±0.01	L
QdfHi.huj.uh-5A.2	5A.3	3.2	39.1	16.7—45.4	28.7	Ra_c69221_1167	0.09	-0.02±0.01	0.07	-0.01±0.02	L
QdHi.huj.uh-5A.3	5A.6	3.9	110.9	97.7—138.7	41.0	Ku_c21235_676	0.15	0.02±0.02			G
QHi.huj.uh-5B	5B.4	5.7	98.4	86.5—105.0	18.6	Tdurum_contig27797_654	0.03	0.00±0.01	0.09	0.03±0.00	G

QTL effects	QTL	LOD	Position (cM)	Interval (cM)	Length (cM)	Nearest marker	WL		WW		ITV Allele
							PEV	d	PEV	d	
QddfHi.huj.uh-6A	6A.1	4.5	7.4	2.1—15.0	12.9	Excalibur_c20597_569	0.11	-0.02±0.00			L
QHi.huj.uh-6B	6B.3	5.8	51.5	46.6—54.3	7.7	BobWhite_c1059_1825	0.06	0.02±0.00	0.06	0.02±0.01	G
QdfHi.huj.uh-6B	6B.3	5.1	49.7	44.5—55.2	10.7	IACX203	0.10	0.02±0.00	0.07	0.02±0.01	G
QHi.huj.uh-7B	7B.1	23.9	14.9	13.9—15.9	1.9	Tdurum_contig42423_1963	0.34	0.05±0.00	0.15	0.03±0.01	G
QdHi.huj.uh-7B	7B.1	7.0	14.9	12.7—20.9	8.1	Tdurum_contig42423_1963	0.19	0.03±0.00			G
**Biomass related traits**											
**Spike dry matter**											
QdfSpdm.huj.uh-2A	2A.1	3.1	38.3	34.6—44.9	10.3	BobWhite_c10977_834	0.04	-2.50±3.09	0.11	1-0.49±8.61	L
QSpdm.huj.uh-2B	2B.6	13.9	103.8	102.2—105.7	3.5	BobWhite_c47357_535	0.14	-8.78±1.60	0.17	-18.64±2.87	L
QdSpdm.huj.uh-2B	2B.6	2.5	103.8	95.4—118.3	22.9	BobWhite_c47357_535	0.09	-5.86±3.34	--	--	L
QdSpdm.huj.uh-4B.1	4B.1	2.0	8.4	0.0—18.0	18.0	RAC875_rep_c106667_302	0.09	-5.16±4.26	--	--	L
QddfSpdm.huj.uh-4B.1	4B.1	2.5	8.4	0.0—16.0	15.9	RAC875_rep_c106667_302	0.13	-6.90±1.90	--	--	L
QSpdm.huj.uh-4B.2	4B.3	2.8	40.5	21.9—45.4	23.5	RFL_Contig3563_1130	0.06	-5.16±3.11	0.05	-9.02±5.97	L
QSpdm.huj.uh-5A	5A.3	3.4	45.5	33.9—51.8	17.9	wsnp_Ex_rep_c70343_69286072	0.03	-0.86±2.44	0.10	-14.32±3.14	L
QSpdm.huj.uh-5B	5B.3	3.2	75.9	71.8—96.0	24.2	Ex_c24477_484	0.05	3.49±3.72	0.08	11.12±6.28	G
QSpdm.huj.uh-7A.1	7A.1	2.4	7.4	0.0—15.3	15.3	RAC875_c63822_185	0.07	-3.60±8.60	0.08	-11.93±16.04	L
QSpdm.huj.uh-7A.2	7A.3	1.8	39.4	32—52	20	IACX17522	0.07	3.85±8.77	0.08	6.50±18.27	G
QSpdm.huj.uh-7B.1	7B.1	10.6	14.9	5.0—19.1	14.1	Tdurum_contig42423_1963	0.18	9.85±1.86	0.10	13.70±4.21	G
QdSpdm.huj.uh-7B.1	7B.1	6.5	14.9	13.2—20.7	7.5	Tdurum_contig42423_1963	0.19	9.53±1.33			G
QddfSpdm.huj.uh-7B.2	7B.4	2.3	128.4	119.4—128.4	9.0	RFL_Contig3607_648	0.10	5.48±3.04			G
**Vegetative dry matter**											
QVegdm.huj.uh-2A	2A.1	3.7	36.4	33.5—41.2	7.7	wsnp_Ex_c2033_3814035	0.06	-7.99±5.45	0.08	-14.48±8.46	L
QdfVegdm.huj.uh-2A	2A.1	4.4	38.3	35.7—42.5	6.8	BobWhite_c10977_834	0.06	-8.12±3.81	0.07	-13.24±7.53	L
QVegdm.huj.uh-4A	4A.1	4.1	17.1	7.2—19.6	12.4	BS00065863_51	0.13	-12.42±6.56	0.03	-5.79±7.42	L
QdfVegdm.huj.uh-4A	4A.1	3.6	17.1	7.7—19.5	11.8	BS00065863_51	0.11	-10.99±5.77	0.03	-6.70±6.86	L
QdVegdm.huj.uh-4A	4A.1	3.7	17.1	7.2—19.8	12.5	BS00065863_51	0.12	-12.23±4.98	--	--	L
QddfVegdm.huj.uh-4A	4A.1	2.4	17.1	4.0—33.7	29.7	BS00065863_51	0.10	-9.48±6.43	--	--	L
QVegdm.huj.uh-4B	4B.3	3.1	40.5	32.2—45.0	12.9	RFL_Contig3563_1130	0.08	-8.98±5.83	0.09	-8.43±15.27	L
QdfVegdm.huj.uh-4B	4B.3	4.1	40.5	33.9—44.8	10.9	RFL_Contig3563_1130	0.09	-10.38±4.53	0.08	-9.84±13.08	L
QddfVegdm.huj.uh-4B	4B.3	2.3	32.2	29.2—44.8	15.6	TA002925-3757	0.11	-11.71±2.67	--	--	L
QVegdm.huj.uh-5A	5A.2	2.6	29.1	11.9—46.0	34.1	wsnp_CAP7_c2282_1107112	0.05	10.03±7.02	0.03	3.79±7.44	G
QdfVegdm.huj.uh-5A	5A.2	3.0	29.1	14.2—45.4	31.2	wsnp_CAP7_c2282_1107112	0.11	11.48±4.02	0.02	2.14±6.97	G
QdVegdm.huj.uh-5A	5A.2	2.9	29.1	16.5—45.1	28.6	wsnp_CAP7_c2282_1107112	0.13	12.68±5.13	--	--	G
QddfVegdm.huj.uh-5A	5A.2	2.5	29.1	11.6—45.6	33.9	wsnp_CAP7_c2282_1107112	0.11	11.36±3.23	--	--	G
QVegdm.huj.uh-7A	7A.1	2.7	2.8	0.0—10.1	10.1	IAAV6131	0.06	-5.25±7.23	0.09	-12.80±10.69	L
QdfVegdm.huj.uh-7A	7A.1	4.4	7.8	5.8—19.0	13.2	Tdurum_contig13048_89	0.07	-5.81±6.96	0.09	-13.46±9.98	L
QdVegdm.huj.uh-7B	7B.3	2.2	43.0	23.9—57.7	33.8	wsnp_Ex_c23755_32994701	0.09	-9.43±5.71	--	--	L
**Total dry matter**											
QTotdm.huj.uh-2A	2A.1	3.6	36.4	32.1—40.1	8.1	wsnp_Ex_c2033_3814035	0.05	-8.68±7.83	0.09	-23.60±12.50	L

QTL effects	QTL	LOD	Position (cM)	Interval (cM)	Length (cM)	Nearest marker	WL		WW		ITV Allele
							PEV	d	PEV	d	
QdfTotdm.huj.uh-2A	2A.1	3.8	38.3	35.3—44.5	9.2	BobWhite_c10977_834	0.04	-7.34±7.39	0.08	-21.81±12.56	L
QTotdm.huj.uh-2B	2B.6	5.4	103.8	101.7—108.7	7.0	BobWhite_c47357_535	0.06	-11.78±5.00	0.13	-32.15±5.96	L
QdfTotdm.huj.uh-2B	2B.6	3.1	106.4	101.0—112.5	11.5	Tdurum_contig12879_1200	0.05	-6.09±9.44	0.08	-20.50±12.70	L
QTotdm.huj.uh-4A	4A.1	2.9	17.1	5.8—20.3	14.5	BS00065863_51	0.11	-14.49±9.80	0.02	-3.56±10.41	L
QdfTotdm.huj.uh-4A	4A.1	3.5	17.1	5.9—20.3	14.3	BS00065863_51	0.12	-15.01±10.12	0.02	-4.52±10.29	L
QdTotdm.huj.uh-4A	4A.1	2.5	10.2	3.5—33.6	30.1	BS00043286_51	0.13	-17.31±7.74	--	--	L
QddfTotdm.huj.uh-4A	4A.1	2.6	17.1	4.5—32.8	28.3	BS00065863_51	0.14	-17.42±6.84	--	--	L
QTotdm.huj.uh-4B	4B.3	4.6	41.5	36.7—45.3	8.7	Kukri_c32064_629	0.08	-13.14±7.33	0.11	-24.94±16.72	L
QdfTotdm.huj.uh-4B	4B.3	3.3	40.5	32.0—52.6	20.5	RFL_Contig3563_1130	0.07	-10.06±9.23	0.08	-13.30±21.27	L
QTotdm.huj.uh-5A	5A.2	2.0	29.1	17.0—59.7	42.7	wsnp_CAP7_c2282_1107112	0.13	18.44±4.47	--	--	G
QTotdm.huj.uh-7A	7A.1	3.3	6.0	0.0—11.2	11.1	RAC875_c18446_506	0.07	-6.95±11.62	0.09	-20.19±16.29	L
QdfTotdm.huj.uh-7A	7A.1	3.5	6.0	0.0—10.6	10.6	RAC875_c18446_506	0.07	-7.19±11.75	0.09	-21.16±15.67	L
**Morphological traits**											
**Culm length**											
QCl.huj.uh-2B	2B.6	5.8	103.8	101.0—111.4	10.4	BobWhite_c47357_535	0.10	-8.06±1.59	0.05	-5.17±2.92	L
QdCl.huj.uh-2B	2B.6	4.0	103.8	93.8—117.4	23.6	BobWhite_c47357_535	0.12	-7.47±1.69	--	--	L
QCl.huj.uh-3A	3A.4	2.5	58.4	46.0—69.5	23.5	wsnp_Ex_c2331_4369782	0.07	-2.68±6.01	0.08	-4.58±6.02	L
QdfCl.huj.uh-3A	3A.4	4.4	58.0	52.6—71.2	18.5	IAAV3900	0.08	-5.46±1.51	0.07	-5.95±3.07	L
QdCl.huj.uh-3B.1	3B.2	2.7	72.2	60.9—83.0	22.1	BS00032926_51	0.13	-7.70±1.10	--	--	L
QCl.huj.uh-3B	3B.3	5.1	97.2	94.9—104.5	9.7	BS00063624_51	0.08	-6.91±1.54	0.04	-4.72±2.73	L
QCl.huj.uh-4A	4A.3	9.5	41.8	37.5—44.0	6.5	TA004056-0809	0.10	-7.68±1.69	0.13	-9.41±1.93	L
QdfCl.huj.uh-4A	4A.3	7.8	41.8	39.2—44.9	5.7	TA004056-0809	0.10	-6.37±1.30	0.08	-7.26±1.84	L
QCl.huj.uh-4B.1	4B.4	7.2	44.1	38.3—48.4	10.1	Ex_c25467_851	0.09	-7.54±1.79	0.07	-6.89±1.95	L
QdfCl.huj.uh-4B.2	4B.6	4.2	84.6	77.9—84.6	6.7	wsnp_Ex_c19844_28854305	0.03	-2.83±1.87	0.11	-8.33±2.35	L
QddfCl.huj.uh-5A.1	5A.3	3.3	41.9	38.5—43.9	5.4	wsnp_Ra_c14112_22155312	0.11	5.83±2.16	--	--	G
QCl.huj.uh-5A.2	5A.6	2.5	110.9	95.6—129.9	34.4	Ku_c21235_676	--	--	0.11	7.64±5.53	G
QdfCl.huj.uh-5A.3	5A.9	3.8	157.0	152.2—157.1	4.9	IACX5850	0.05	-2.47±4.05	0.05	-4.62±3.78	L
QCl.huj.uh-5B	5B.8	4.4	133.5	129.1—137.7	8.6	BS00050057_51	0.08	6.66±2.35	0.12	8.96±2.21	G
QdfCl.huj.uh-6B	6B.2	4.1	37.1	24.0—47.0	23.1	BS00109708_51	0.10	-6.39±1.41	0.03	-3.54±2.74	L
QddfCl.huj.uh-6B	6B.2	2.5	30.1	22.1—49.9	27.8	TA003005-0339	0.10	-5.90±1.06	--	--	L
QCl.huj.uh-7A.1	7A.5	5.1	73.0	63.5—82.1	18.6	Kukri_rep_c70389_57	0.07	-6.11±2.31	0.04	-4.38±2.80	L
QdfCl.huj.uh-7A.1	7A.5	5.4	78.5	71.4—84.2	12.8	wsnp_Ex_c11636_18742884	0.10	-6.08±2.28	0.05	-5.29±2.62	L
QddfCl.huj.uh-7A.2	7A.7	2.9	104.5	94.7—111.7	17.0	wsnp_Ex_c9428_15641639	0.11	-5.84±1.61	--	--	L
QCl.huj.uh-7B	7B.1	7.5	11.5	5.8—18.1	12.3	wsnp_CAP8_c334_304253	0.10	7.68±1.53	0.08	7.21±1.81	G
**Spike length**											
QSpl.huj.uh-1A	1A.2	3.9	53.0	50.1—56.0	6.0	wsnp_Ex_c572_1138503	0.12	1.49±0.30	0.06	1.29±0.49	G
QdfSpl.huj.uh-1A	1A.2	2.3	53.0	48.8—59.7	10.9	wsnp_Ex_c572_1138503	0.05	0.89±0.28	0.02	0.63±0.69	G
QSpl.huj.uh-2A	2A.5	4.6	130.8	126.3—133.0	6.8	BS00065366_51	0.07	-1.06±0.54	0.02	-0.59±0.68	L
QSpl.huj.uh-4A.1	4A.3	2.6	41.8	22.9—51.1	28.2	TA004056-0809	0.12	-0.99±1.18	--	--	L
QddfSpl.huj.uh-4A.1	4A.3	2.6	41.8	33.0—51.1	18.1	TA004056-0809	0.08	-0.97±0.26	--	--	L

QTL effects	QTL	LOD	Position (cM)	Interval (cM)	Length (cM)	Nearest marker	WL		WW		ITV Allele
							PEV	d	PEV	d	
QSpl.huj.uh-4A.2	4A.6	3.4	101.4	90.8—108.9	18.1	RAC875_c35979_263	--	--	0.10	1.82±0.62	G
QSpl.huj.uh-5A	5A.3	10.8	41.9	38.6—43.5	4.9	wsnp_Ra_c14112_22155312	0.17	1.89±0.24	0.03	0.87±0.41	G
QdfSpl.huj.uh-5A	5A.3	10.1	41.9	39.0—43.7	4.7	wsnp_Ra_c14112_22155312	0.18	1.86±0.28	0.04	1.13±0.44	G
QdSpl.huj.uh-5A	5A.3	5.5	39.8	36.1—45.2	9.1	BS00077990_51	0.17	1.58±0.24	--	--	G
QddfSpl.huj.uh-5A	5A.3	4.5	39.1	32.8—56.0	23.1	Ra_c69221_1167	0.16	1.42±0.21	--	--	G
QSpl.huj.uh-5B	5B.8	7.4	133.5	130.4—136.3	5.9	BS00050057_51	0.06	1.13±0.25	0.08	1.63±0.37	G
QSpl.huj.uh-6A	6A.4	5.1	69.4	58.4—72.9	14.6	BobWhite_c47143_592	0.09	-1.34±0.22	0.02	-0.72±0.47	L
QdfSpl.huj.uh-6A	6A.4	4.0	69.4	59.62—76.34	16.7	BobWhite_c47143_592	0.08	-1.15±0.46	0.02	-0.56±0.59	L
QdSpl.huj.uh-6A	6A.4	2.3	74.1	54.7—98.4	43.7	RFL_Contig5314_1147	0.09	-1.11±0.23	--	--	L
QddfSpl.huj.uh-6A	6A.4	2.0	73.1	50.1—103.8	53.7	BS00065152_51	0.07	-0.91±0.28	--	--	L
QdfSpl.huj.uh-6B	6B.2	3.1	36.8	30.0—50.6	20.6	IACX6023	0.05	0.96±0.33	0.04	1.09±0.45	G
QSpl.huj.uh-7A	7A.5	5.5	79.6	74.0—86.3	12.3	Kukri_c64283_212	0.08	1.27±0.22	0.04	1.08±0.40	G
QdfSpl.huj.uh-7A	7A.5	3.8	80.7	72.8—91.9	19.1	Kukri_c26517_691	0.09	1.25±0.30	0.03	0.93±0.51	G
QSpl.huj.uh-7B	7B.1	8.2	14.9	13.4—21.2	7.8	Tdurum_contig42423_1963	0.10	-1.45±0.26	0.06	-1.45±0.39	L
**Flag leaf length**											
QFll.huj.uh-1B	1B.1	3.2	6.2	0.4—11.4	11.0	TA012713-0667	0.06	-1.31±0.65	0.06	-1.52±0.61	L
QFll.huj.uh-2A	2A.4	2.1	82.5	70.3—94.6	24.3	Excalibur_c34913_743	0.08	1.55±0.75	0.06	1.49±0.78	G
QFll.huj.uh-3A	3A.5	2.9	72.4	68.8—78.2	9.3	IAAV8483	0.12	-2.09±0.44	0.03	-0.80±0.86	L
QFll.huj.uh-4B	4B.4	3.0	45.5	41.1—51.0	9.9	RAC875_c18736_133	0.12	1.92±0.94	0.05	1.02±1.08	G
QFll.huj.uh-5A.1	5A.3	4.5	41.9	38.8—45.5	6.7	wsnp_Ra_c14112_22155312	0.25	1.92±1.47	0.16	1.37±1.12	G
QdfFll.huj.uh-5A.1	5A.3	5.4	43.3	38.5—47.1	8.5	BobWhite_rep_c49979_373	0.22	1.64±0.96	0.14	1.11±1.05	G
QddfFll.huj.uh-5A.1	5A.3	2.8	43.3	37.4—52.7	15.3	BobWhite_rep_c49979_373	0.14	0.26±1.95	--	--	G
QFll.huj.uh-5A.2	5A.8	7.7	146.9	141.8—154.1	12.3	wsnp_Ex_rep_c101323_86702546	0.25	-2.05±1.19	0.16	-2.03±0.95	L
QdfFll.huj.uh-5A.2	5A.8	6.4	153.5	144.4—148.4	4.0	tplb0049a09_1302	0.22	-1.62±0.95	0.14	-2.00±0.84	L
QdFll.huj.uh-5A.2	5A.8	3.3	145.8	140.6—157.0	16.5	wsnp_Ex_c3136_5798236	0.13	-1.23±1.87	--	--	L
QFll.huj.uh-6A.1	6A.1	3.0	11.3	0.5—24.5	24.0	Tdurum_contig97611_150	0.17	-0.04±2.50	0.17	-1.78±2.25	L
QdFll.huj.uh-6A.1	6A.1	2.0	12.3	0.0—29.3	29.3	Tdurum_contig54957_624	0.08	-1.71±0.38	--	--	L
QFll.huj.uh-6A.2	6A.4	2.6	69.4	63.4—86.0	22.7	BobWhite_c47143_592	0.11	-1.07±1.57	0.11	-0.24±1.99	L
QdfFll.huj.uh-6A.2	6A.4	3.3	69.4	66.23—72.12	5.9	BobWhite_c47143_592	0.05	-1.20±0.48	0.08	-1.79±0.74	L
QFll.huj.uh-7A	7A.3	2.3	36.8	26.2—40.5	14.3	Tdurum_contig82438_73	0.08	-1.37±1.09	0.07	-1.34±1.12	L
**Flag leaf width**											
QFlw.huj.uh-1A	1A.1	4.1	27.6	8.1—32.0	23.9	GENE-0002_856	0.03	-0.07±0.02	0.05	-0.11±0.02	L
QdfFlw.huj.uh-1A	1A.1	4.7	11.3	6.6—16.0	9.4	Excalibur_c9676_163	0.04	-0.07±0.02	0.03	-0.09±0.02	L
QFlw.huj.uh-2A	2A.2	3.0	55.1	32.1—63.2	31.1	Kukri_rep_c73477_888	0.10	-0.12±0.03	0.05	-0.09±0.05	L
QdfFlw.huj.uh-2A	2A.2	2.9	56.6	34.6—64.2	29.6	Tdurum_contig5311_67	0.11	-0.12±0.03	0.05	-0.09±0.05	L
QFlw.huj.uh-2B.1	2B.6	5.5	103.8	98.6—110.8	12.2	BobWhite_c47357_535	0.02	-0.04±0.03	0.08	-0.15±0.02	L
QdfFlw.huj.uh-2B.1	2B.6	6.7	106.4	98.1—111.5	13.4	Tdurum_contig12879_1200	0.02	-0.05±0.03	0.07	-0.13±0.03	L
QdFlw.huj.uh-2B.2	2B.7	2.1	118.4	110.5—132.5	22.1	BS00068181_51	0.08	0.08±0.01	--	--	G
QFlw.huj.uh-3A	3A.1	4.1	8.3	0.9—17.0	16.1	Ra_c6118_350	0.07	0.09±0.05	0.10	0.14±0.06	G
QdfFlw.huj.uh-3A	3A.1	5.2	11.9	6.9—18.3	11.4	wsnp_JD_c2722_3653988	0.03	0.06±0.03	0.04	0.10±0.03	G
QFlw.huj.uh-3B	3B.4	6.7	118.2	115.9—122.4	6.5	BobWhite_c15763_205	0.05	0.09±0.02	0.05	0.11±0.03	G

QTL effects	QTL	LOD	Position (cM)	Interval (cM)	Length (cM)	Nearest marker	WL		WW		ITV Allele
							PEV	d	PEV	d	
QdfFlw.huj.uh-3B	3B.4	5.0	129.9	124.9—133.6	8.6	Tdurum_contig100787_79	0.05	0.09±0.02	0.03	0.08±0.03	G
QFlw.huj.uh-4A.1	4A.5	2.8	74.4	62.7—78.9	16.2	Kukri_c19216_123	0.03	0.02±0.07	0.10	0.12±0.10	G
QFlw.huj.uh-4A.2	4A.7	6.2	120.9	118.1—123.5	5.4	RAC875_rep_c69632_65	0.13	0.01±0.08	0.09	0.07±0.12	G
QdfFlw.huj.uh-4A.2	4A.7	4.3	120.9	113.1—123.9	10.8	RAC875_rep_c69632_65	0.00	0.00±0.03	0.06	0.12±0.03	G
QdFlw.huj.uh-4A.2	4A.7	3.7	120.9	110.2—125.0	14.8	RAC875_rep_c69632_65	0.13	0.10±0.02	--	--	G
QddfFlw.huj.uh-4A.2	4A.7	3.0	120.9	108.7—125.6	16.9	RAC875_rep_c69632_65	0.11	0.08±0.05	--	--	G
QFlw.huj.uh-4B	4B.5	26.6	61.0	60.3—63.0	2.7	RAC875_c24515_602	0.25	-0.20±0.03	0.25	-0.26±0.03	L
QdfFlw.huj.uh-4B	4B.5	32.4	61.0	60.4—62.2	1.8	RAC875_c24515_602	0.28	-0.21±0.02	0.27	-0.26±0.03	L
QFlw.huj.uh-5A	5A.5	18.8	100.3	98.0—102.9	4.9	Tdurum_contig50175_875	0.20	-0.18±0.02	0.12	-0.18±0.03	L
QdfFlw.huj.uh-5A	5A.5	16.1	99.5	97.8—102	4.2	RAC875_rep_c109969_119	0.14	-0.15±0.02	0.08	-0.14±0.03	L
QdFlw.huj.uh-5A	5A.5	2.9	97.0	91.8—120.8	29.0	Excalibur_c23354_306	0.12	-0.10±0.02	--	--	L
QddfFlw.huj.uh-5A	5A.5	2.3	97.0	91.0—126.7	35.7	Excalibur_c23354_306	0.12	-0.10±0.02	--	--	L
QdfFlw.huj.uh-5B.1	5B.3	4.8	56.1	49.0—67.5	18.5	Ra_c35412_1057	0.11	-0.01±0.13	0.14	-0.07±0.10	L
QdfFlw.huj.uh-5B.2	5B.7	3.0	117.4	107.0—135.0	28.0	BS00098292_51	0.11	0.04±0.11	0.14	0.12±0.07	G
QdfFlw.huj.uh-5B.3	5B.8	6.1	132.8	126.5—137.6	11.1	BS00084106_51	--	--	0.08	0.14±0.03	G
QFlw.huj.uh-6A	6A.2	8.8	49.1	45.6—52.5	6.9	IACX14305	0.06	-0.10±0.02	0.06	-0.13±0.03	L
QdfFlw.huj.uh-6A	6A.2	8.3	49.1	45.03—53.47	8.4	IACX14305	0.05	-0.09±0.02	0.05	-0.12±0.02	L
QFlw.huj.uh-6B	6B.4	3.2	51.5	47.4—58.4	11.0	BobWhite_c1059_1825	--	--	0.05	-0.10±0.04	L
QdfFlw.huj.uh-7A	7A.6	4.8	87.3	81.8—101.2	19.4	BS00109319_51	0.03	0.06±0.02	0.06	0.12±0.02	G
QFlw.huj.uh-7B	7B.4	5.7	128.4	125.4—128.4	3.0	RFL_Contig3607_648	0.11	-0.13±0.03	0.10	-0.14±0.04	L
**Drought adaptive physiological traits**											
**Carbon isotope ratio**											
Qδ13c.huj.uh-1A	1A.2	4.4	59.8	48.7—66.9	18.2	IAAV5535	--	--	0.13	0.36±0.07	L
Qdfδ13c.huj.uh-1A	1A.2	4.5	59.8	48.4—67.3	18.9	IAAV5535	0.02	0.03±0.08	0.12	0.34±0.08	L
Qδ13c.huj.uh-3A.1	3A.1	2.2	8.3	0.0—18.1	18.0	Ra_c6118_350	0.07	-0.07±0.19	0.11	-0.03±0.16	G
Qddfδ13c.huj.uh-3A.1	3A.1	2.5	11.9	3.4—18.0	14.6	wsnp_JD_c2722_3653988	0.10	-0.22±0.09	--	--	G
Qδ13c.huj.uh-3A.2	3A.5	6.6	68.1	64.9—73.8	8.9	wsnp_Ex_c18223_27035083	0.07	-0.06±0.13	0.11	-0.25±0.18	G
Qdfδ13c.huj.uh-3A.2	3A.5	5.7	68.1	65.0—74.9	9.9	wsnp_Ex_c18223_27035083	0.02	-0.05±0.08	0.13	-0.36±0.10	G
Qδ13c.huj.uh-4A	4A.3	8.0	41.8	31.4—45.8	14.4	TA004056-0809	0.14	-0.30±0.06	0.06	-0.23±0.08	G
Qdfδ13c.huj.uh-4A	4A.3	9.3	36.4	31.7—45.6	13.8	RAC875_c31051_490	0.19	-0.35±0.06	0.06	-0.25±0.08	G
Qdδ13c.huj.uh-4A	4A.3	3.1	24.0	22.6—47.6	25.0	Kukri_rep_c101034_516	0.16	-0.30±0.06	--	--	G
Qddfδ13c.huj.uh-4A	4A.3	3.1	36.4	23.2—48.3	25.1	RAC875_c31051_490	0.11	-0.25±0.04	--	--	G
Qδ13c.huj.uh-5A.1	5A.4	5.6	69.2	63.3—73.9	10.6	Kukri_c63163_141	0.10	-0.25±0.06	0.07	-0.26±0.09	G
Qdfδ13c.huj.uh-5A.1	5A.4	7.0	68.1	63.1—72.4	9.4	Ex_c104539_35	0.11	-0.26±0.06	0.08	-0.28±0.07	G
Qdδ13c.huj.uh-5A.2	5A.7	2.3	133.7	129.1—140.5	11.4	wsnp_Ex_c54211_57168122	0.10	-0.26±0.04	--	--	G
Qδ13c.huj.uh-6B	6B.4	5.1	55.7	42.6—60.5	18.0	Tdurum_contig93283_513	0.08	-0.22±0.05	0.07	-0.27±0.06	G
Qdfδ13c.huj.uh-6B	6B.4	4.3	55.7	43.1—61.5	18.5	Tdurum_contig93283_513	0.07	-0.21±0.06	0.06	-0.24±0.09	G
Qδ13c.huj.uh-7B	7B.2	5.6	23.3	14.6—28.0	13.4	Tdurum_contig99020_139	0.14	-0.31±0.04	0.01	-0.03±0.08	G
Qdδ13c.huj.uh-7B	7B.2	6.0	23.7	21.0—27.5	6.5	BobWhite_c44404_312	0.17	-0.33±0.05	--	--	G
Qddfδ13c.huj.uh-7B	7B.2	3.9	23.7	21.5—31.7	10.2	BobWhite_c44404_312	0.13	-0.27±0.07	--	--	G

QTL effects	QTL	LOD	Position (cM)	Interval (cM)	Length (cM)	Nearest marker	WL		WW		ITV Allele
							PEV	d	PEV	d	
**Osmotic potential at heading**											
QdfOp.huj.uh-1A	1A.3	2.3	94.3	81.3—110.6	29.3	Ra_c41164_730	--	--	0.11	0.74±0.13	L
QOp.huj.uh-2A	2A.3	3.9	64.3	52.8—70.8	18.0	Tdurum_contig27887_55	--	--	0.15	0.96±0.12	L
QdfOp.huj.uh-2A	2A.3	4.0	66.2	51.4—71.6	20.2	wsnp_Ex_c22862_32074455	0.02	0.00±0.39	0.13	0.80±0.28	L
QOp.huj.uh-2B	2B.3	3.2	75.6	66.5—87.9	21.4	RFL_Contig4718_1269	0.04	0.36±0.22	0.14	0.62±0.19	L
QdfOp.huj.uh-2B	2B.3	4.0	66.2	51.4—71.6	20.2	wsnp_Ex_c22862_32074455	0.02	0.00±0.39	0.13	0.80±0.28	L
QOp.huj.uh-3B	3B.2	4.5	64.7	61.8—72.0	10.2	BS00032926_51	0.10	0.88±0.19	0.08	0.65±0.22	L
QdfOp.huj.uh-3B	3B.2	3.8	72.6	65.5—75.7	10.2	RAC875_c16281_195	0.10	0.81±0.28	0.06	0.52±0.25	L
QOp.huj.uh-4B	4B.5	3.7	60.7	50.3—70.8	20.5	Tdurum_contig59914_323	0.16	1.12±0.24	0.04	0.16±0.44	L
QdfOp.huj.uh-4B	4B.5	3.3	65.2	50.6—59.6	9.0	IAAV8848	0.12	0.87±0.35	0.05	0.25±0.47	L
QdOp.huj.uh-4B	4B.5	3.4	65.2	55.3—75.2	19.9	IAAV8848	0.19	1.11±0.24	--	--	L
QddfOp.huj.uh-4B	4B.5	3.3	65.2	55.0—75.7	20.8	IAAV8848	0.18	1.07±0.30	--	--	L
QdOp.huj.uh-5A	5A.6	2.1	128.0	99.8—145.0	45.1	wsnp_Ex_c54655_57455110	0.11	0.62±0.17	--	--	L
QOp.huj.uh-5B	5B.5	2.9	106.9	103.6—109.5	5.9	BobWhite_c28333_454	0.07	-0.50±0.20	0.08	-0.46±0.19	G
QdfOp.huj.uh-5B	5B.5	2.9	106.9	103.3—109.8	6.5	BobWhite_c28333_454	0.07	-0.49±0.23	0.06	-0.37±0.21	G
QOp.huj.uh-6A	6A.3	2.8	53.5	46.7—62.8	16.1	Tdurum_contig76709_195	--	--	0.10	0.58±0.12	L
QdfOp.huj.uh-6A	6A.3	3.2	53.5	44.7—59.5	14.8	Tdurum_contig76709_195	0.02	0.11±0.27	0.11	0.59±0.12	L
QOp.huj.uh-6B	6B.2	4.5	36.4	32.0—39.2	7.2	RAC875_c13920_747	0.05	0.40±0.18	0.10	0.58±0.13	L
QdfOp.huj.uh-6B	6B.2	4.9	36.4	32.1—39.1	7.0	RAC875_c13920_747	0.05	0.45±0.17	0.12	0.61±0.12	L
**Chlorophyll content at heading**											
QChl.huj.uh-2A	2A.2	3.0	43.6	38.1—57.6	19.6	Excalibur_rep_c105284_110	--	--	0.11	-2.47±0.39	L
QdfChl.huj.uh-2A	2A.2	3.8	43.6	36.9—51.7	14.9	Excalibur_rep_c105284_110	0.08	-1.85±0.60	0.09	-2.12±0.69	L
QdfChl.huj.uh-2B.1	2B.3	3.3	78.2	69.0—88.0	19.0	Kukri_c4097_898	0.06	-0.10±1.81	0.07	-1.27±0.98	L
QChl.huj.uh-2B.2	2B.5	4.4	98.5	77.7—111.0	33.2	Excalibur_rep_c86807_132	0.05	-1.15±1.23	0.11	-1.95±0.78	L
QChl.huj.uh-3A	3A.4	2.4	60.1	49.6—69.1	19.5	BS00011612_51	--	--	0.06	0.99±1.08	G
QdChl.huj.uh-4A	4A.2	2.1	28.4	22.1—34.5	12.5	Ku_c3891_395	0.09	1.41±1.38	--	--	G
QddfChl.huj.uh-4A	4A.2	2.0	28.4	22.1—34.7	12.5	Ku_c3891_395	0.09	1.45±1.36	--	--	G
QChl.huj.uh-4B.1	4B.1	2.9	4.1	0.0—21.4	21.4	IAAV7323	0.09	1.99±0.64	0.08	1.81±0.80	G
QdfChl.huj.uh-4B.1	4B.2	7.0	16.0	12.9—20.8	7.9	Tdurum_contig76213_1156	0.09	2.19±0.65	0.11	2.04±0.52	G
QChl.huj.uh-5A	5A.3	4.6	45.5	33.2—53.4	20.2	wsnp_Ex_rep_c70343_69286072	0.06	-1.60±0.85	0.10	-1.98±0.43	L
QdfChl.huj.uh-5A	5A.3	3.8	35.4	32.8—41	8.2	BS00021873_51	0.05	-1.34±0.85	0.13	-2.04±0.51	L
QChl.huj.uh-5B	5B.6	4.7	112.2	103.8—119.6	15.9	Tdurum_contig70554_1004	0.09	1.97±0.52	0.12	2.49±0.51	G
QdfChl.huj.uh-5B	5B.6	2.9	111.8	96.3—122.4	26.1	Tdurum_contig47833_484	0.06	0.71±1.62	0.10	1.41±1.28	G
QChl.huj.uh-6A.1	6A.2	2.8	48.4	38.3—54.3	16.0	wsnp_Ex_c19770_28768859	0.09	-1.93±0.73	0.03	-0.96±0.93	L
QdfChl.huj.uh-6A.1	6A.2	2.8	47.7	37.3—53.9	16.6	BS00012297_51	0.08	-1.84±0.76	0.05	-1.30±0.92	L
QdChl.huj.uh-6A.1	6A.2	2.5	47.7	40.2—52.8	12.6	BS00012297_51	0.10	-1.85±0.78	--	--	L
QddfChl.huj.uh-6A.1	6A.2	2.5	47.7	40.0—52.8	12.8	BS00012297_51	0.09	-1.74±0.94	--	--	L
QdfChl.huj.uh-6A.2	6A.4	6.4	65.8	63.6—68.3	4.7	wsnp_Ra_c12086_19452422	0.04	1.44±0.63	0.12	2.13±0.41	G
QdfChl.huj.uh-6B	6B.5	4.7	99.8	93.0—105.0	12.1	Excalibur_c4261_342	0.08	2.07±0.51	0.07	1.69±0.37	G
QChl.huj.uh-7A	7A.6	4.8	86.6	78.9—91.8	12.9	Kukri_rep_c105999_572	0.07	1.78±0.80	0.09	1.86±0.41	G

QTL effects	QTL	LOD	Position (cM)	Interval (cM)	Length (cM)	Nearest marker	WL		WW		ITV Allele
							PEV	d	PEV	d	
QdfChl.huj.uh-7A	7A.6	2.8	86.6	77.5—92.2	14.7	Kukri_rep_c105999_572	0.06	1.17±1.36	0.08	1.48±0.73	G
QdfChl.huj.uh-7B	7B.4	5.2	128.4	121.8—128.4	6.6	RFL_Contig3607_648	0.04	-1.47±0.63	0.10	-2.00±0.40	L
**Flag leaf rolling index**											
QdLr.huj.uh-1B	1B.3	2.1	40.1	28.6—52.0	23.4	IAAV1158	0.10	-0.31±0.16	--	--	L
QLr.huj.uh-2A	2A.5	3.3	133.7	126.9—140.0	13.1	Tdurum_contig93508_295	0.06	0.27±0.10	0.09	0.33±0.10	G
QdfLr.huj.uh-2A	2A.5	3.2	134.4	129.9—140.7	10.8	BS00022813_51	0.06	0.26±0.10	0.08	0.28±0.10	G
QLr.huj.uh-5A	5A.3	5.4	47.7	43.9—56.1	12.3	Excalibur_c53930_53	0.07	0.28±0.11	0.13	0.39±0.08	G
QdfLr.huj.uh-5A	5A.3	5.5	47.7	43.5—54.7	11.2	Excalibur_c53930_53	0.06	0.27±0.11	0.14	0.40±0.08	G
QLr.huj.uh-5B	5B.3	3.4	83.3	60.5—86.4	25.9	RAC875_c32611_347	0.08	0.29±0.12	0.10	0.33±0.09	G
QLr.huj.uh-7A	7A.4	5.8	61.3	56.1—64.3	8.1	RAC875_c67063_703	0.14	-0.43±0.09	0.05	-0.23±0.10	L
QdfLr.huj.uh-7A	7A.4	5.9	61.3	56.1—64.4	8.3	RAC875_c67063_703	0.14	-0.43±0.08	0.05	-0.23±0.09	L
QdLr.huj.uh-7A	7A.4	3.0	61.3	55.3—65.5	10.2	RAC875_c67063_703	0.12	-0.37±0.08	--	--	L
QddfLr.huj.uh-7A	7A.4	3.1	61.3	53.2—65.6	12.4	RAC875_c67063_703	0.14	-0.38±0.08	--	--	L
**Phenology related traits**											
**Days from planting to heading**											
QDph.huj.uh-1B	1B.1	3.3	6.2	0.0—8.7	8.7	TA012713-0667	0.08	-2.39±2.66	0.08	-1.46±2.16	L
QDph.huj.uh-2B	2B.6	21.7	106.4	103.0—107.9	5.0	Tdurum_contig12879_1200	0.17	5.03±0.58	0.14	3.51±0.48	G
QDph.huj.uh-3A	3A.3	7.5	44.6	38.7—49.0	10.3	wsnp_Ex_rep_c101942_87217430	0.03	-2.01±0.60	0.07	-2.48±0.45	L
QDph.huj.uh-4B	4B.4	2.4	45.5	37.1—50.5	13.4	RAC875_c18736_133	0.08	3.35±0.96	0.07	2.44±0.91	G
QDph.huj.uh-5A.1	5A.3	2.8	41.9	36.9—49.9	12.9	wsnp_Ra_c14112_22155312	0.11	1.71±2.45	0.07	0.60±1.90	G
QDph.huj.uh-5A.2	5A.5	4.9	100.3	88—106.5	18.5	Tdurum_contig50175_875	0.11	-3.82±1.28	0.11	-2.98±0.91	L
QDph.huj.uh-5A.3	5A.8	7.1	146.9	140.3—149.7	9.3	wsnp_Ex_rep_c101323_86702546	0.11	-2.38±2.02	0.07	-1.28±1.62	L
QDph.huj.uh-5B	5B.7	4.6	120.3	112.7—123.7	11.0	wsnp_RFL_Contig1548_762547	0.03	-2.20±0.56	0.04	-1.81±0.43	L
QDph.huj.uh-6B	6B.1	2.4	3.9	0.0—17.0	16.9	CAP11_c1087_327	0.08	-3.19±1.24	0.07	-2.21±1.13	L
QDph.huj.uh-7B	7B.1	40.8	14.9	14.1—15.7	1.6	Tdurum_contig42423_1963	0.37	-7.45±0.62	0.37	-5.75±0.50	L
**Days from heading to maturity**											
QDhm.huj.uh-2B	2B.6	10.4	106.4	102.6—108.4	5.8	Tdurum_contig12879_1200	0.15	-3.47±0.46	0.08	-1.38±0.33	L
QdDhm.huj.uh-2B	2B.6	4.5	110.7	101.4—115.7	14.3	Ra_c13298_783	0.12	-2.50±1.46	--	--	L
QDhm.huj.uh-4A	4A.3	4.2	44.3	36.7—51.9	15.2	wsnp_Ex_c10886_17694220	0.02	-0.77±0.67	0.10	-1.52±0.26	L
QdfDhm.huj.uh-4A	4A.3	4.1	45.0	36.2—54.7	18.5	BS00022174_51	0.04	-0.63±0.35	0.09	-1.29±0.32	L
QDhm.huj.uh-5A.1	5A.3	4.5	41.9	37.4—45.7	8.4	wsnp_Ra_c14112_22155312	0.16	-1.21±3.64	0.11	-0.40±1.34	L
QDhm.huj.uh-5A.2	5A.8	5.9	146.2	141.4—151.3	9.9	Ku_c19516_384	0.16	1.06±3.11	0.11	0.54±1.30	G
QDhm.huj.uh-5B	5B.7	3.8	120.3	112.2—128.3	16.1	wsnp_RFL_Contig1548_762547	0.04	1.64±0.57	0.05	1.04±0.38	G
QDhm.huj.uh-6A	6A.1	3.0	14.1	7.0—27.9	20.9	IAAV2577	0.06	1.84±1.13	0.11	1.50±0.38	G
QDhm.huj.uh-6B	6B.3	3.9	38.2	31.4—46.7	15.3	CAP7_c6528_99	0.02	-0.28±0.65	0.13	-1.73±0.44	L
QDhm.huj.uh-7B	7B.1	13.6	14.9	12.9—16.4	3.5	Tdurum_contig42423_1963	0.28	4.70±0.50	--	--	G
QdDhm.huj.uh-7B	7B.1	13.1	14.9	11.6—16.4	4.9	Tdurum_contig42423_1963	0.32*	-4.63±0.62*			G

Table S14 Summary of QTLs. Summary of QTLs detected for 17 observed traits, 16 traits adjusted to heading and 33 drought plasticity traits in G×L RIL population under two water regimes.

QTL	Interval, cM	LOD	# of trait effects	ITV alleles	Traits	Phenology	Drought	Drought strategy	Candidate genes
1A.1	6.6—32.3	4.1—4.7	single	--	FLW	non-plastic	non-plastic	--	--
1A.2	48.4—67.3	2.3—4.5	multi	opposite	SpL, δ13C	non-plastic	non-plastic	avoidance	*TaGID1-A1, TaGA20ox-A4, GASA family*
1A.3	81.3—110.6	2.3—4.7	multi	opposite	TKW, KNSP, OP	plastic	plastic	tolerance	*GASA family, TaGA2ox-A8*
1A.4	98.4—117.4	3.8	single	--	HI	plastic	plastic	--	--
1B.1	0.0—11.4	3.2—3.3	multi	uniform	DPH, FLL	linked	non-plastic	--	--
1B.2	10.3—22.3	3.1—5.3	single	--	TKW	non-plastic	non-plastic	--	--
1B.3	28.6—52.0	2.1	single	--	LR	non-plastic	plastic	avoidance*	*Glu-B3, TaGA2ox-B1, TaGA2ox-B10, TaGID1-B1, TaGA20ox-B4, GASA family*
2A.1	32.7—44.9	3.1—4.4	multi	uniform	GY, KNSP, SpDM, VegDM, TotDM	plastic	non-plastic	--	*Ppd-A1*
2A.2	34.6—64.2	2.9—3.8	multi	uniform	Chl, FLW	non-plastic	non-plastic	stay green	*TaGA13ox-A2*
2A.3	51.4—70.9	3.9—4.0	single	--	OP	non-plastic	non-plastic	tolerance	*FPF1-like, TaFT4-A1*
2A.4	70.3—94.6	2.1	single	--	FLL	non-plastic	non-plastic	--	*TaFT9-A1, TaGA2ox-A6*
2A.5	126.3—140.7	3.3—4.6	multi	opposite	SpL, LR	non-plastic	non-plastic	avoidance	*TaGly1/Zeo*
2B.1	18.6—36.0	3.3—5.9	single	--	TKW	non-plastic	non-plastic	--	--
2B.2	27.9—52.8	2.1—5.8	multi	uniform	HI, GY	non-plastic	non-plastic	--	*TaGA13ox-B2, FPF1-like, TaFT4-B1*
2B.3	65.9—88.0	3.2—4.0	multi	uniform	OP, Chl	plastic	non-plastic	stay green, tolerance	*TaFT9-B1, TaGA2ox-B6*
2B.4	82.8—89.3	3.7—8.9	multi	opposite	KNSP, TKW	non-plastic	plastic	--	--
2B.5	77.7—111.0	4.4—9.3	multi	uniform	HI, Chl	non-plastic	non-plastic	--	--
2B.6	95.4—118.3	2.5—21.7	multi	opposite	DPH, DHM, GY, KNSP, SpDM, CL, FLW, TotDM	linked	plastic	escape	*TaFT8-B1*

QTL	Interval, cM	LOD	# of trait effects	ITV alleles	Traits	Phenology	Drought	Drought strategy	Candidate genes
2B.7	110.4—132.5	2.1—2.3	multi	uniform	TKW, FLW	non-plastic	plastic	--	*TaFT8-B2, TaFT8-B3, TaGA1ox-B1, TaGA3ox-B3*
3A.1	0.0—18.3	2.2—5.2	multi	opposite	FLW, δ13C	non-plastic	plastic	avoidance*	--
3A.2	15.4—23.4	5.6	single	--	TKW	linked	non-plastic	--	*TaGID2-A1*
3A.3	38.7—49.0	7.5	single	--	DPH	linked	non-plastic	--	*TaGI-A, TaGA3ox, TaGA2ox, TaFT2-A1, FPF1-like, TaGA2ox-A3*
3A.4	46.0—71.2	2.4—4.4	multi	opposite	Chl, Cl	non-plastic	non-plastic	stay green	--
3A.5	64.9—78.0	2.9—6.6	multi	uniform	FLL, δ13C	non-plastic	non-plastic	avoidance	--
3A.6	127.2—135.4	4.2	single	--	KNSP	plastic	non-plastic	--	--
3B.1	13.7—40.1	3.5—3.9	single	--	HI	plastic	plastic	--	--
3B.2	60.9—87.5	2.7—4.5	multi	uniform	GY, CL, OP	non-plastic	plastic	tolerance*	--
3B.3	94.9—104.5	5.1	single	--	CL	linked	non-plastic	--	--
3B.4	115.9—133.6	5.0—6.7	single	--	FLW	non-plastic	non-plastic	--	--
4A.1	3.5—33.6	2.2—4.1	multi	opposite	HI, TotDM, VegDM	non-plastic	plastic	--	--
4A.2	22.1—74.6	2.0—2.1	single	--	Chl	plastic	plastic	stay green*	*TaGA2ox-A11*
4A.3	23.2—54.7	2.6—9.5	multi	uniform	CL, DHM, TKW, SpL, δ13C	non-plastic	plastic	avoidance*	*GID1, TaPRR73-A, TaGA13ox-A1*
4A.4	35.9—74.6	3.2—6.6	single	--	TKW	plastic	plastic	--	*GASA_family, TaFT11-A1*
4A.5	62.7—78.9	2.8	single	--	FLW	linked	non-plastic	--	*TaGA20ox-A1*
4A.6	79.1—119.5	2.4—4.1	multi	opposite	GY, HI, SpL	non-plastic	non-plastic	--	*Wx-B1*
4A.7	108.7—125.6	3.0—6.2	single	--	FLW	non-plastic	plastic	--	--
4B.1	0.0—21.4	2.0—2.9	multi	opposite	SpDM, Chl	non-plastic	plastic	stay green	--
4B.2	8.2—23.3	7.0	single	--	Chl	plastic	non-plastic	stay green	--
4B.3	21.9—52.6	2.3—6.1	multi	uniform	GY, TKW, SpDM, VegDM, TotDM	non-plastic	plastic	--	*TaGA13ox-B1, TaGA2ox-B11, GID1*
4B.4	37.1—51.0	2.4—7.2	multi	opposite	DPH, FLL, CL	linked	non-plastic	--	--
4B.5	50.3—75.7	3.3—32.4	multi	uniform	FLW, OP	non-plastic	plastic	tolerance	--
4B.6	77.9—84.6	4.2	single	--	CL	plastic	non-plastic	--	*TaGA2ox*
5A.1	0.0—7.9	4.7	single	--	TKW	plastic	non-plastic	--	--
5A.2	11.9—59.7	2.0—4.4	multi	opposite	HI, VegDM, TotDM	non-plastic	plastic	--	--

QTL	Interval, cM	LOD	# of trait effects	ITV alleles	Traits	Phenology	Drought	Drought strategy	Candidate genes
5A.3	32.3—56.1	2.8—11.2	multi	opposite	DPH, DHM, GY, KNSP, HI, FLL, SpL, CL, Chl, SpDM, LR	non-plastic	plastic	avoidance	*TaFT7-A1, FPA*
5A.4	63.1—73.9	5.6—7.0	single	--	δ13C	non-plastic	non-plastic	avoidance	*TaFT10-A, FPA*
5A.5	88.0—120.8	2.3—18.8	multi	uniform	FLW, DPH	linked	plastic	--	*VRN-A1, GASA_family*
5A.6	95.6—145.0	2.1—3.9	multi	opposite	HI, CL, OP	non-plastic	plastic	tolerance	--
5A.7	129.1—144.0	2.3—3.6	multi	opposite	KNSP, δ13C	linked	plastic	avoidance	*TaGA2ox-A8, FPF1*
5A.8	140.3—157.0	3.3—7.7	multi	opposite	DHP, DHM, FLL	linked	plastic	escape	*FPF1*
5A.9	152.2—157.1	3.8	single	--	CL	plastic	non-plastic	--	*TaFT5-A1*
5B.1	37.8—46.5	2.0—5.2	single	--	TKW	non-plastic	non-plastic	--	*GASA_family*
5B.2	49.0—67.5	4.8	single	--	FLW	plastic	non-plastic	--	--
5B.3	60.5—96.0	3.2—3.4	multi	uniform	LR, SpDM	linked	non-plastic	--	*TaFT10-B1, FPA,*
5B.4	86.5—105.0	5.7	single	--	HI	linked	non-plastic	--	*VRN-B1, GASA_family*
5B.5	103.3—109.8	2.9	single	--	OP	non-plastic	non-plastic	tolerance	--
5B.6	96.3—135.0	2.9—4.7	single	--	Chl	non-plastic	non-plastic	stay green	--
5B.7	112.2—128.3	3.0—4.6	multi	opposite	DPH, DHM, FLW	linked	non-plastic	--	--
5B.8	126.5—137.6	4.4—7.4	multi	uniform	CL, SpL, FLW	linked	non-plastic	--	*WAP2-B*
6A.1	0.0—29.3	2.0—4.5	multi	opposite	HI, FLL, DHM	linked	plastic	escape	--
6A.2	37.3—57.0	2.3—8.8	multi	uniform	FLW, KNSP, Chl	non-plastic	plastic	stay green	*TaFT6-A1*
6A.3	44.7—63.3	2.4—3.5	multi	uniform	TKW, OP	non-plastic	non-plastic	tolerance	*TaFT12-A1, TaGA2ox-A9*
6A.4	58.4—90.2	2.0—6.4	multi	opposite	FLL, SpL, Chl	plastic	plastic	stay green	--
6B.1	0.0—17.0	2.4	single	--	DPH	linked	non-plastic	--	--
6B.2	22.1—49.9	2.5—4.9	multi	opposite	CL, SpL, OP	plastic	plastic	tolerance	--
6B.3	31.4—49.8	2.3—3.9	multi	uniform	TKW, DHM	non-plastic	non-plastic	--	*Gpc-B1*
6B.4	42.6—61.5	3.2—5.8	multi	opposite	HI, FLW, δ13C	non-plastic	non-plastic	avoidance	*TaFT6-B1, TaFT12-B, TaGA2ox-B9*
6B.5	93.0—105.0	4.7	single	--	Chl	plastic	non-plastic	stay green	--
7A.1	0.0—19.0	2.4—4.4	multi	uniform	SpDM, VegDM, TotDM	non-plastic	non-plastic	--	*KAO1*
7A.2	14.6—26.5	4.7	single	--	TKW	plastic	non-plastic	--	*Wx-A1*
7A.3	26.2—40.5	2.3	single	--	FLL, SpDM	non-plastic	non-plastic	--	--
7A.4	53.2—65.6	3.0—5.9	single	--	LR	non-plastic	plastic	avoidance	*TaSS-A1*
7A.5	63.5—91.9	3.8—5.5	multi	opposite	CL, SpL, TKW	non-plastic	non-plastic	--	*GID1, TaFT4-A2, KO-A*
7A.6	73.3—102.7	2.8—4.8	multi	opposite	FLW, Chl, KNSP	plastic	non-plastic	--	*TaGA2ox-A2*

QTL	Interval, cM	LOD	# of trait effects	ITV alleles	Traits	Phenology	Drought	Drought strategy	Candidate genes
7A.7	94.7—111.7	2.9	single	--	CL	non-plastic	plastic	--	--
7B.1	4.7—29.5	2.8—40.8	multi	opposite	GY, HI, KNSP, TKW, DPH, DHM, SpDM, SpL, CL	linked	plastic	escape	*VRN-B3*
7B.2	14.6—31.7	3.9—6.0	single	--	δ13C	non-plastic	plastic	avoidance	--
7B.3	23.9—57.7	2.2	single	--	VegDM	non-plastic	plastic	--	*GASA_family*
7B.4	113.2—128.4	—	multi	opposite	KNSP, SpDM, Chl, FLW	plastic	plastic	stay green	*CPS-B*

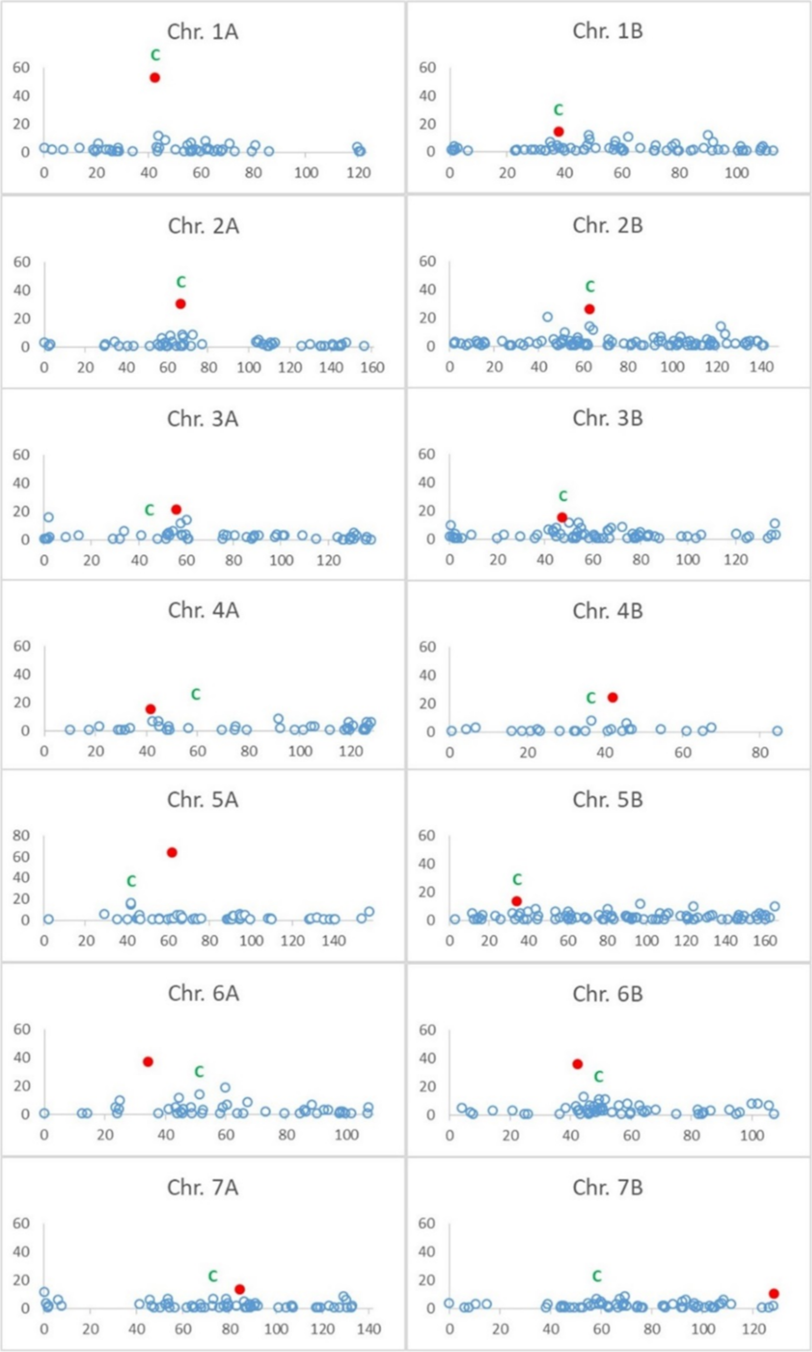

Figure S1 Distribution of attached markers along 14 wheat chromosomes.
Unique genetic positions on the genetic map (Skeletal markers) include attached markers that are completely linked. The distributions of attached markers are shown for each chromosome in a separated plot. The position with the highest number of attached markers in each chromosome is marked by red circle; the putative position of the centromere is marked with green “C”.

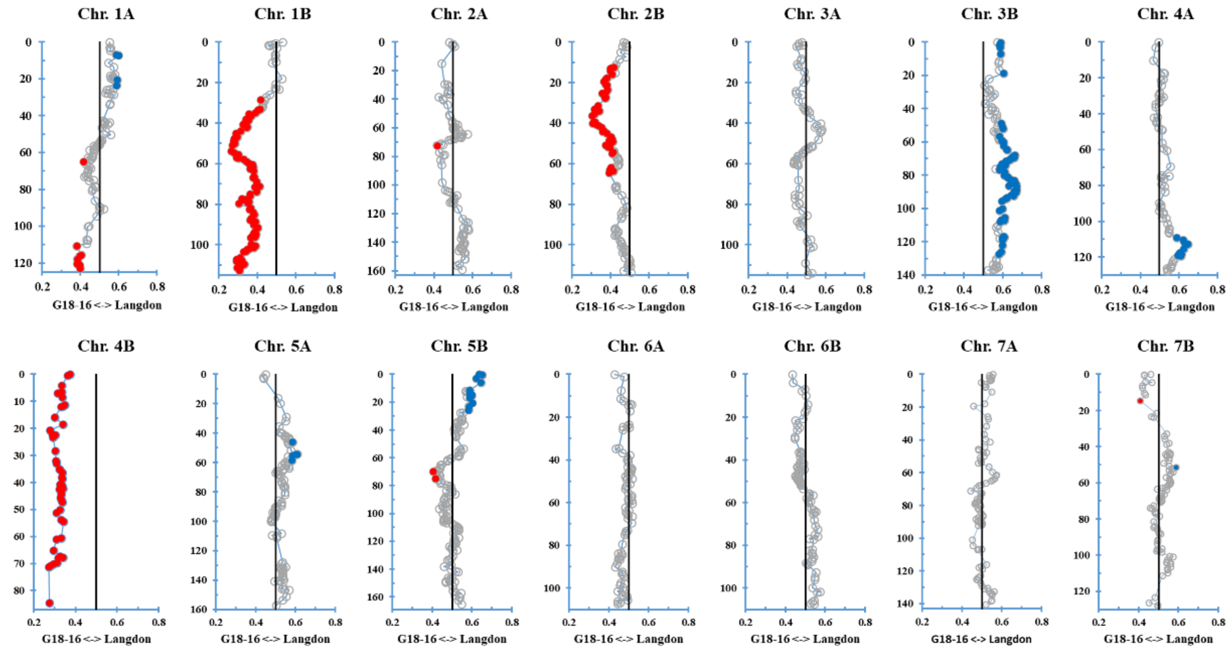

Figure S2 Segregation distortion for each chromosome in the G×L RIL population. Markers with significant (P<0.05) distortion are shown in color, dark blue for LDN and red for G18-16 alleles excess.

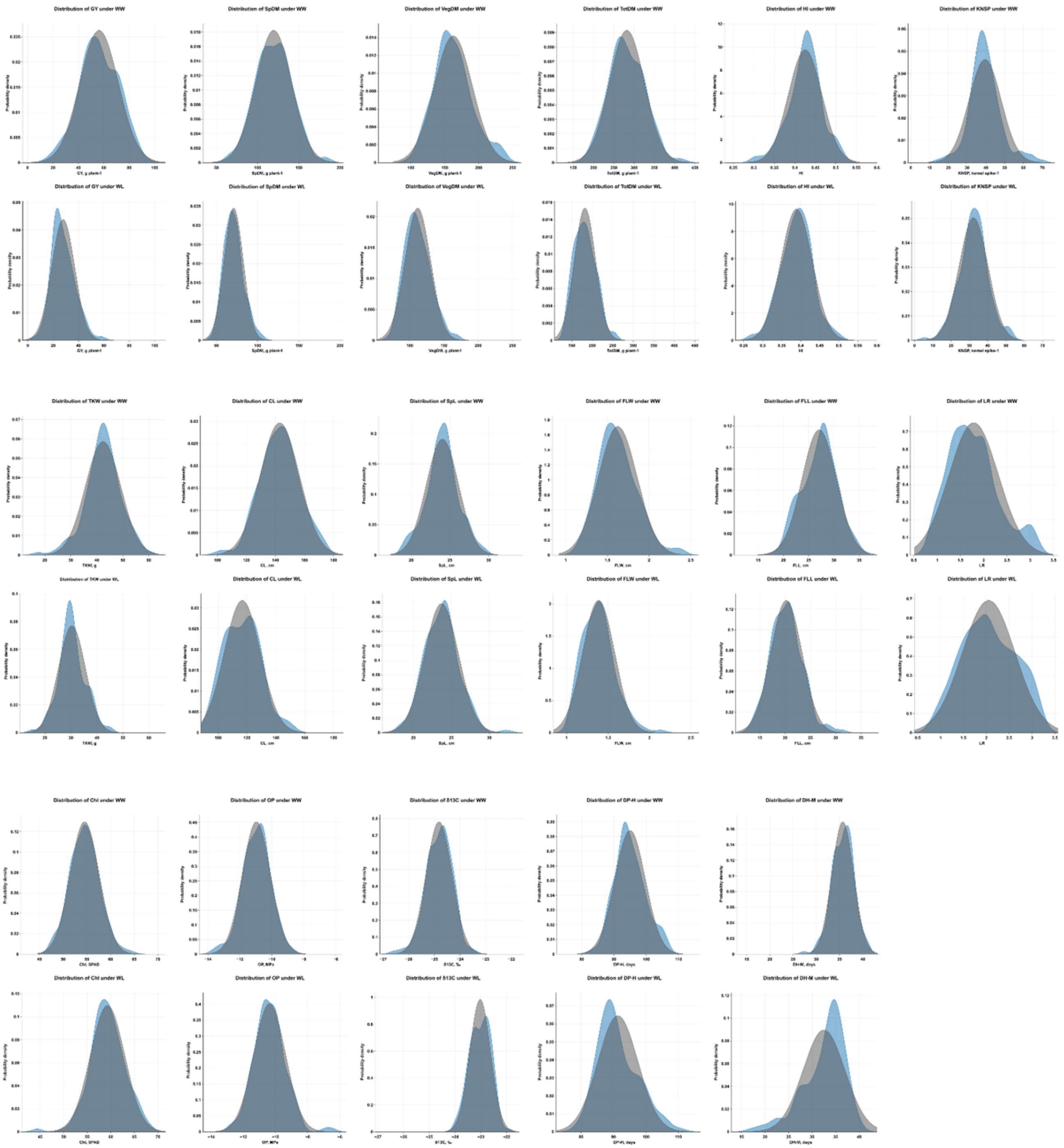

Figure S3 Frequency distribution of the 150 F6 RILs for 17 observed phenotypic traits. Phenotypic distribution of 17 observed traits demonstrated by frequency of the trait among 150 F_6 RILs. (a) Grain yield (GY), (b) thousand kernel weight (TKW), (c) kernel number per spike (KNSP), (d) harvest index (HI), (e) spike dry matter (SpDM), (f) vegetative dry matter (VegDM), (g) total dry matter (TotDM), (h) culm length (CL), (i) spike length (SpL), (j) flag leave length (FLL), (k) flag leave width (FLW), (l) carbon isotope ratio (δ13C), (m) osmotic potential (OP), (n) chlorophyll content (Chl), (o) leave rolling index (LR), (p) days from planting to heading (DP-H), (q) days from heading to maturity (DH-M) under WL and WW irrigation regimes. The expected normal distribution for each trait is presented in gray, while the observed distribution is presented in blue.

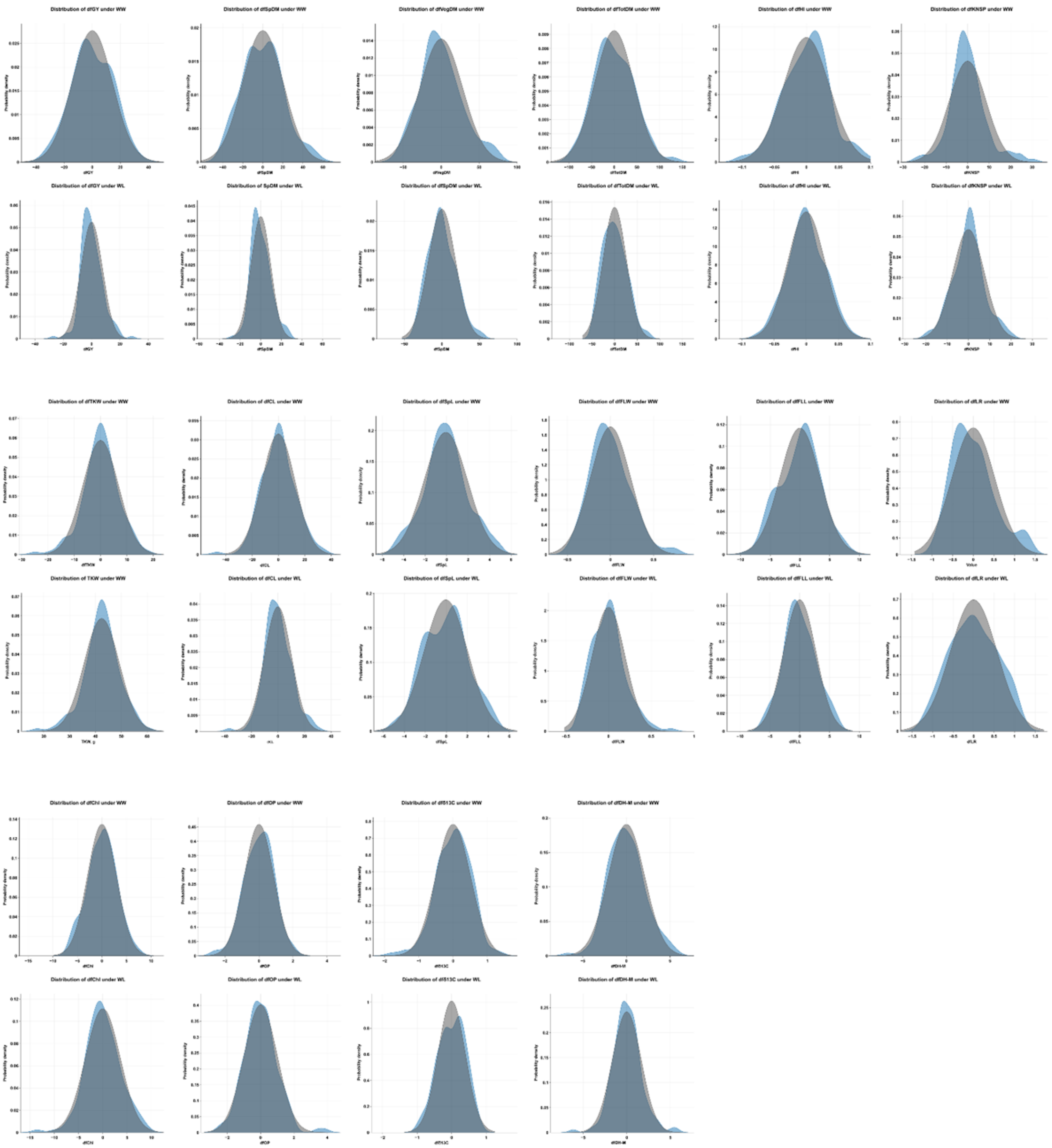

Figure S4 Frequency distribution of the 150 F6 RILs for 16 adjusted for heading date traits. Phenotypic distribution of 16 adjusted traits for heading demonstrated by frequency of the trait among 150 F6 RILs. (a) Grain yield (GY), (b) thousand kernel weight (TKW), (c) kernel number per spike (KNSP), (d) harvest index (HI), (e) spike dry matter (SpDM), (f) vegetative dry matter (VegDM), (g) total dry matter (TotDM), (h) culm length (CL), (i) spike length (SpL), (j) flag leave length (FLL), (k) flag leave width (FLW), (l) carbon isotope ratio (δ13C), (m) osmotic potential (OP), (n) chlorophyll content (Chl), (o) leave rolling index (LR), (p) days from planting to heading (DP-H), (q) days from heading to maturity (DH-M) under WL and WW irrigation regimes. The expected normal distribution for each trait is presented in gray, while the observed distribution is presented in blue.

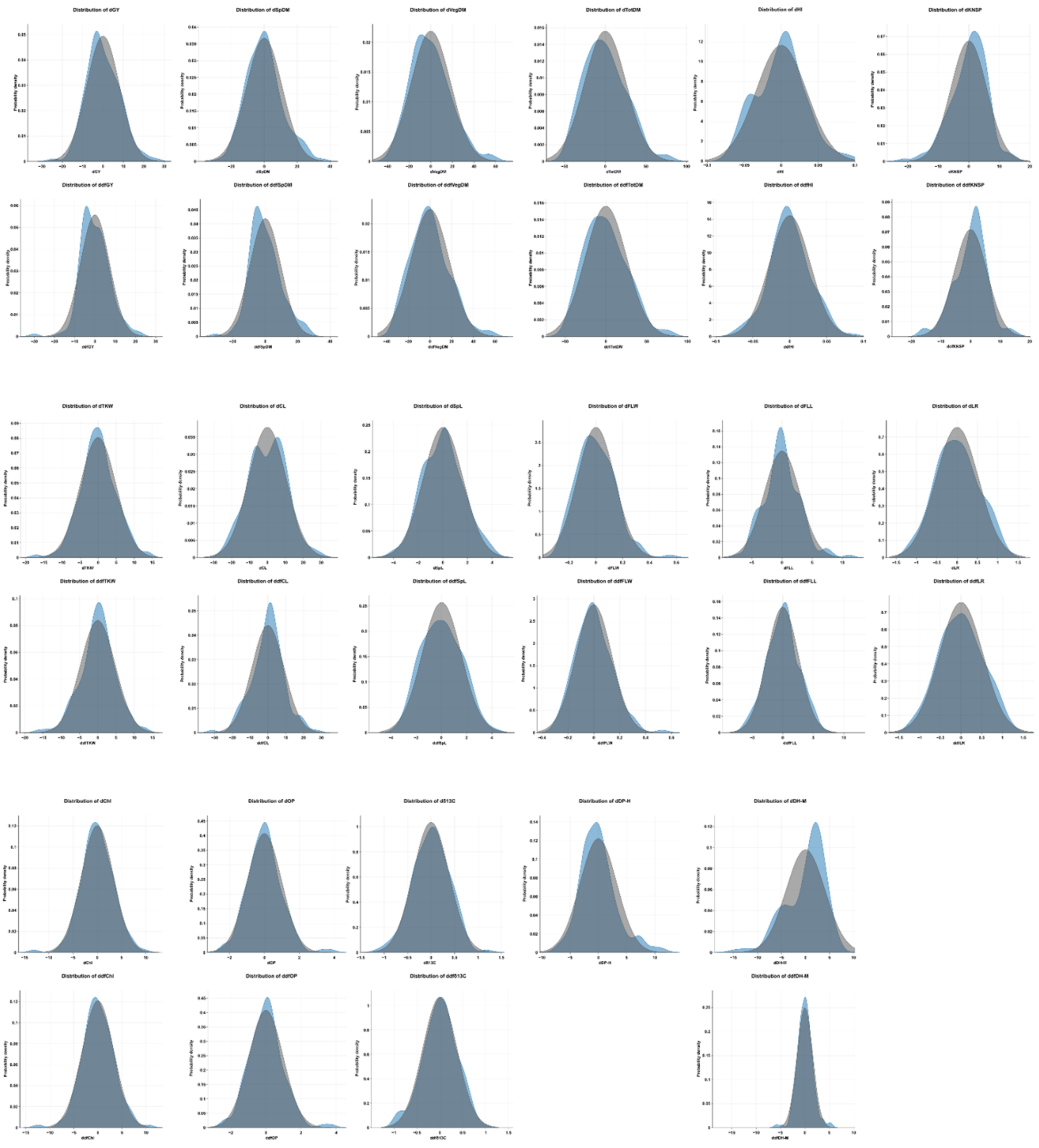

Figure S5 Frequency distribution of the 150 F6 RILs for 33 drought plasticity traits. Phenotypic distribution of 33 drought plasticity traits demonstrated by frequency of the trait among 150 F_6 RILs. (a) Grain yield (GY), (b) thousand kernel weight (TKW), (c) kernel number per spike (KNSP), (d) harvest index (HI), (e) spike dry matter (SpDM), (f) vegetative dry matter (VegDM), (g) total dry matter (TotDM), (h) culm length (CL), (i) spike length (SpL), (j) flag leave length (FLL), (k) flag leave width (FLW), (l) carbon isotope ratio (δ13C), (m) osmotic potential (OP), (n) chlorophyll content (Chl), (o) leave rolling index (LR), (p) days from planting to heading (DP-H), (q) days from heading to maturity (DH-M) under WL and WW irrigation regimes. The expected normal distribution for each trait is presented in gray, while the observed distribution is presented in blue.

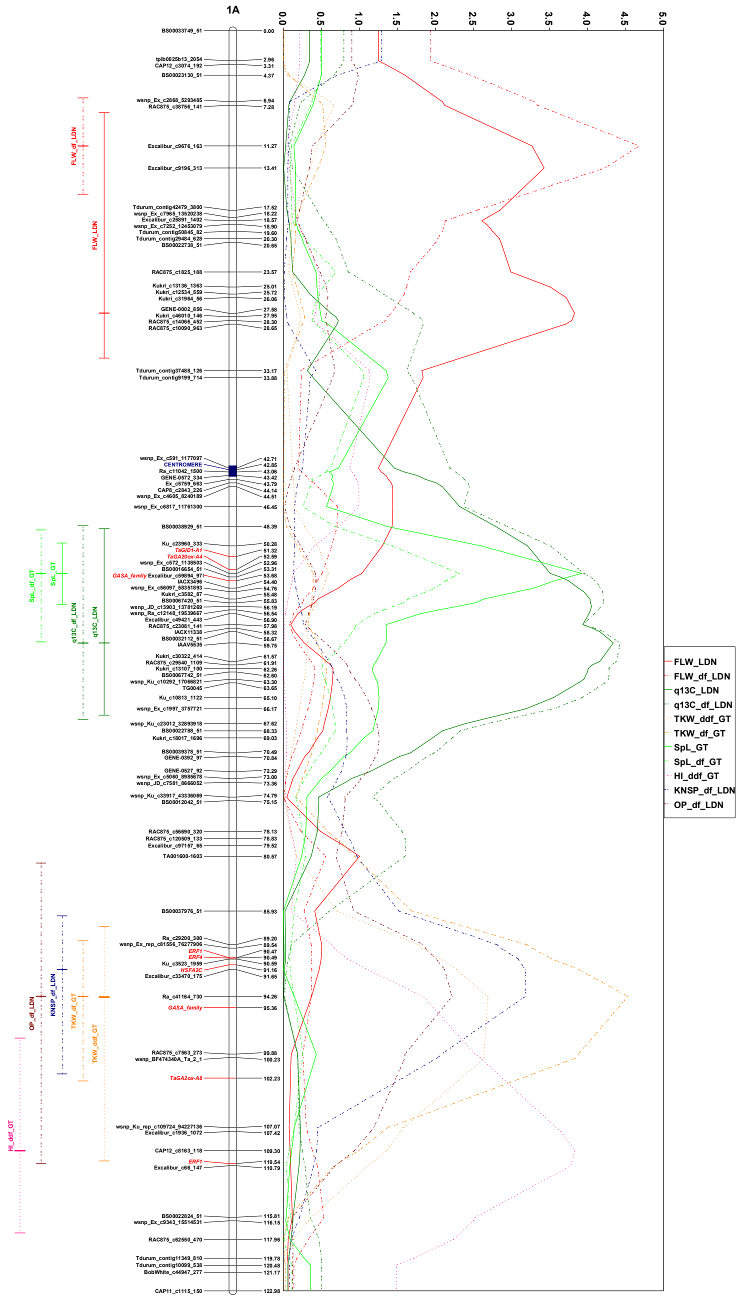

Figure S6 Genetic map, putative genetic positions of target genes, likelihood plots and 1.5 LOD support intervals of QTL effects for chromosome 1A.

Genetic map based on skeletal markers, putative genetic positions of target genes were marked in red. Likelihood plots and 1.5 LOD support intervals were named according to corresponding traits, where first part is name of trait, where name of trait without suffix is name of observed trait (solid line), suffix “df” is marked traits accounting to heading (dash line), suffix “d” is marked plasticity traits trait without accounting to heading (dash line with dots) and suffix “ddf” is marked plasticity traits trait with accounting to heading (dotted line). Last part of name includes association with increase trait value allele: G18-16 allele is named with suffix “GT” and Langdon allele is named with suffix “LDN”.

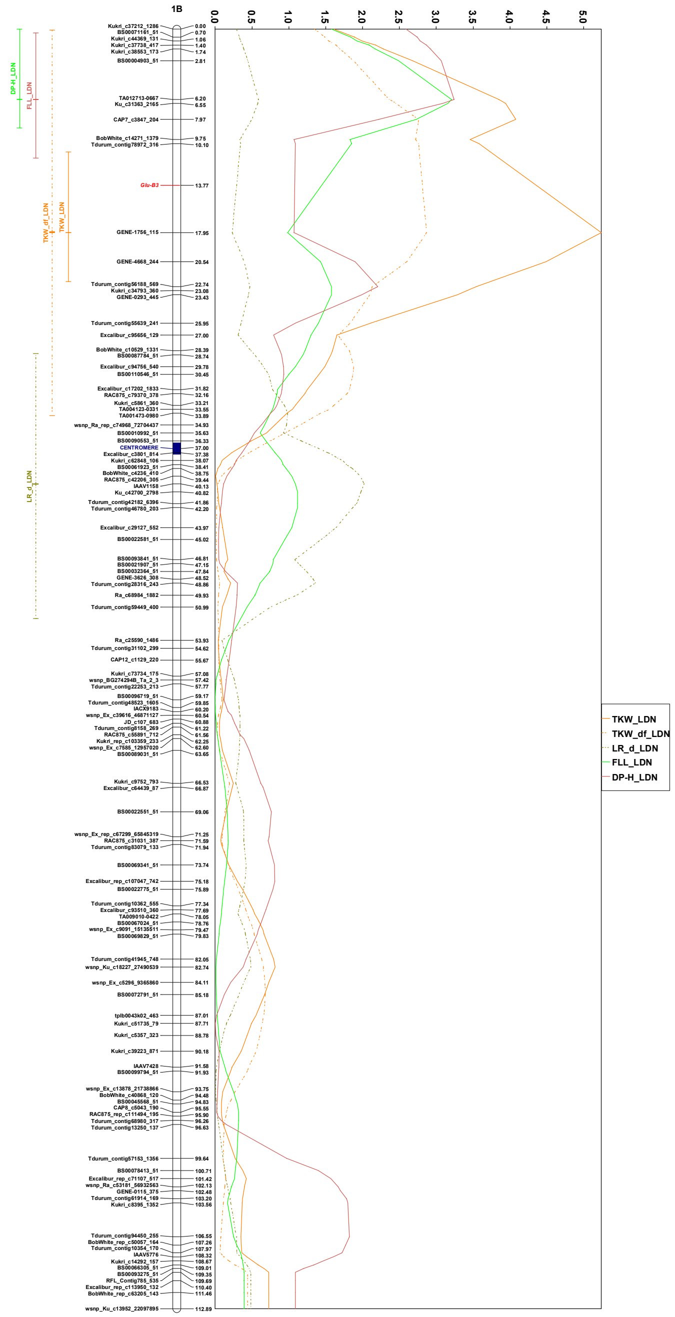

Figure S7 Genetic map, putative genetic positions of target genes, likelihood plots and 1.5 LOD support intervals of QTL effects for chromosome 1B.

Genetic map based on skeletal markers, putative genetic positions of target genes were marked in red. Likelihood plots and 1.5 LOD support intervals were named according to corresponding traits, where first part is name of trait, where name of trait without suffix is name of observed trait (solid line), suffix “df” is marked traits accounting to heading (dash line), suffix “d” is marked plasticity traits trait without accounting to heading (dash line with dots) and suffix “ddf” is marked plasticity traits trait with accounting to heading (dotted line). Last part of name includes association with increase trait value allele: G18-16 allele is named with suffix “GT” and Langdon allele is named with suffix “LDN”.

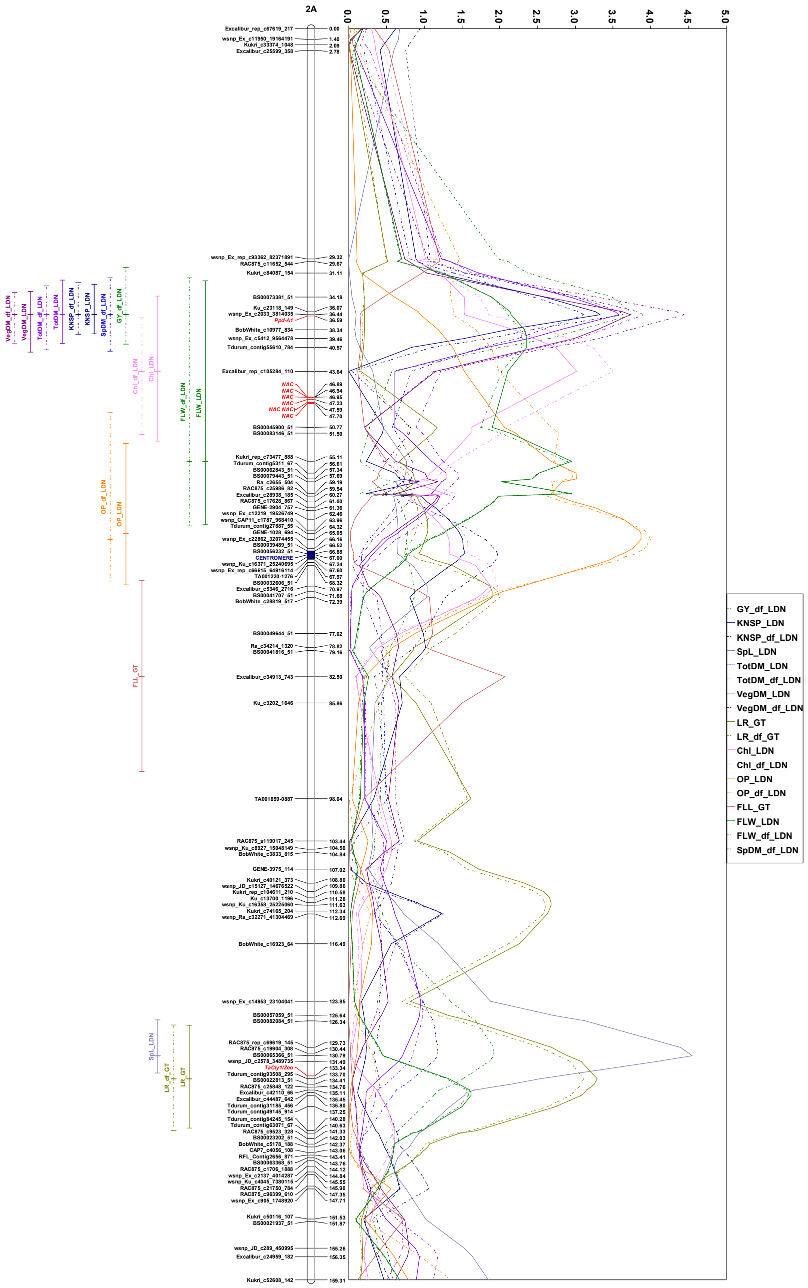

Figure S8 Genetic map, putative genetic positions of target genes, likelihood plots and 1.5 LOD support intervals of QTL effects for chromosome 2A.

Genetic map based on skeletal markers, putative genetic positions of target genes were marked in red. Likelihood plots and 1.5 LOD support intervals were named according to corresponding traits, where first part is name of trait, where name of trait without suffix is name of observed trait (solid line), suffix “df” is marked traits accounting to heading (dash line), suffix “d” is marked plasticity traits trait without accounting to heading (dash line with dots) and suffix “ddf” is marked plasticity traits trait with accounting to heading (dotted line). Last part of name includes association with increase trait value allele: G18-16 allele is named with suffix “GT” and Langdon allele is named with suffix “LDN”.

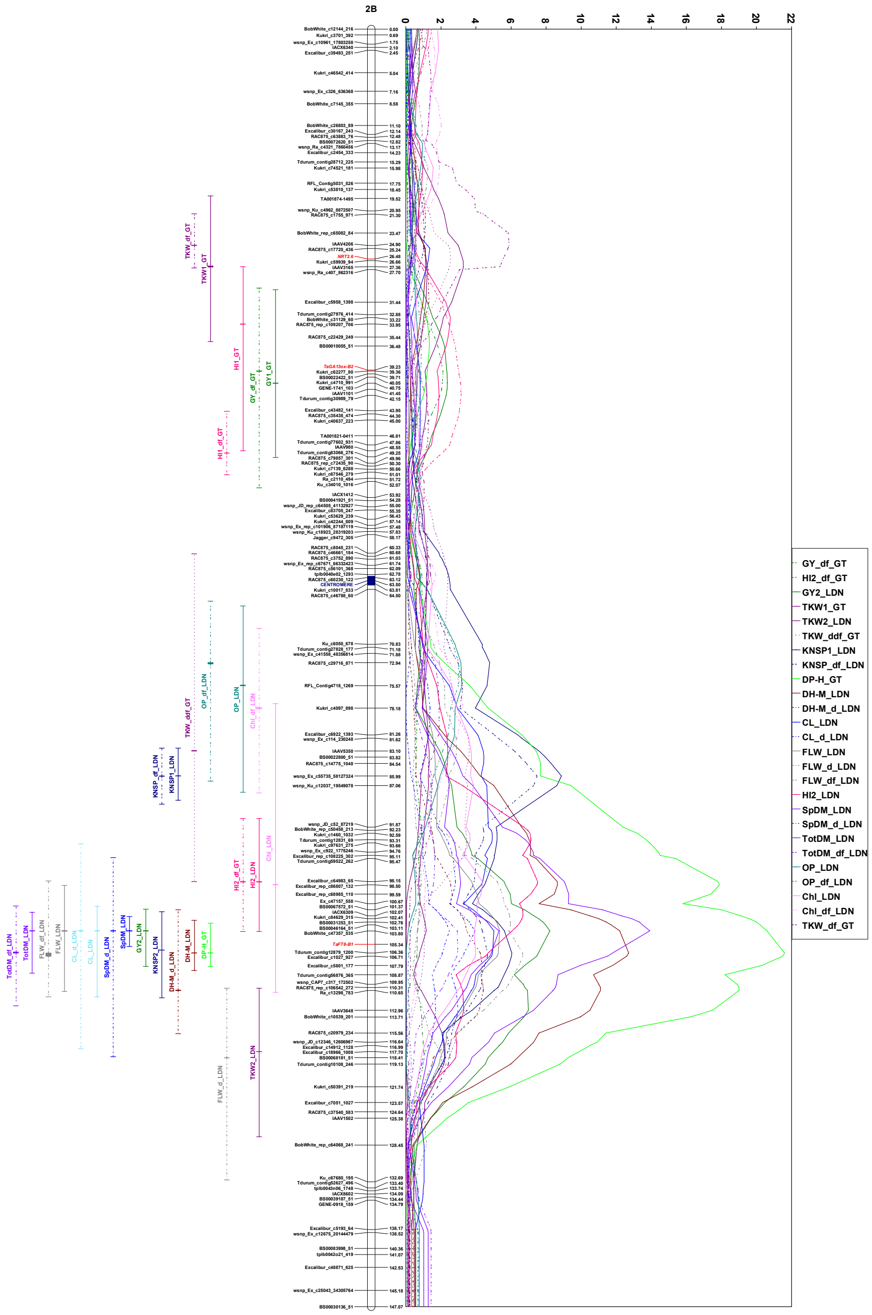

Figure S9 Genetic map, putative genetic positions of target genes, likelihood plots and 1.5 LOD support intervals of QTL effects for chromosome 2B.

Genetic map based on skeletal markers, putative genetic positions of target genes were marked in red. Likelihood plots and 1.5 LOD support intervals were named according to corresponding traits, where first part is name of trait, where name of trait without suffix is name of observed trait (solid line), suffix "df" is marked traits accounting to heading (dash line), suffix "d" is marked plasticity traits trait without accounting to heading (dash line with dots) and suffix "ddf" is marked plasticity traits trait with accounting to heading (dotted line). Last part of name includes association with increase trait value allele: G18-16 allele is named with suffix "GT" and Langdon allele is named with suffix "LDN".

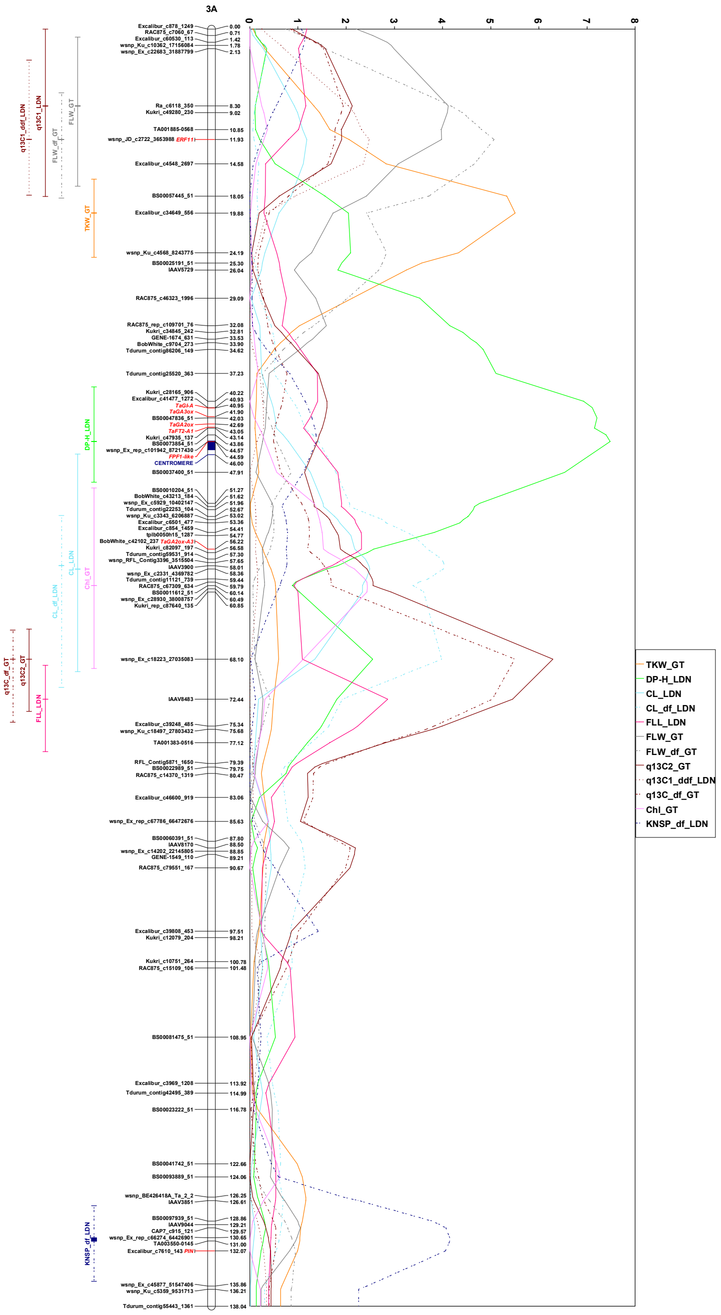

Figure S10 Genetic map, putative genetic positions of target genes, likelihood plots and 1.5 LOD support intervals of QTL effects for chromosome 3A.

Genetic map based on skeletal markers, putative genetic positions of target genes were marked in red. Likelihood plots and 1.5 LOD support intervals were named according to corresponding traits, where first part is name of trait, where name of trait without suffix is name of observed trait (solid line), suffix “df” is marked traits accounting to heading (dash line), suffix “d” is marked plasticity traits trait without accounting to heading (dash line with dots) and suffix “ddf” is marked plasticity traits trait with accounting to heading (dotted line). Last part of name includes association with increase trait value allele: G18-16 allele is named with suffix “GT” and Langdon allele is named with suffix “LDN”.

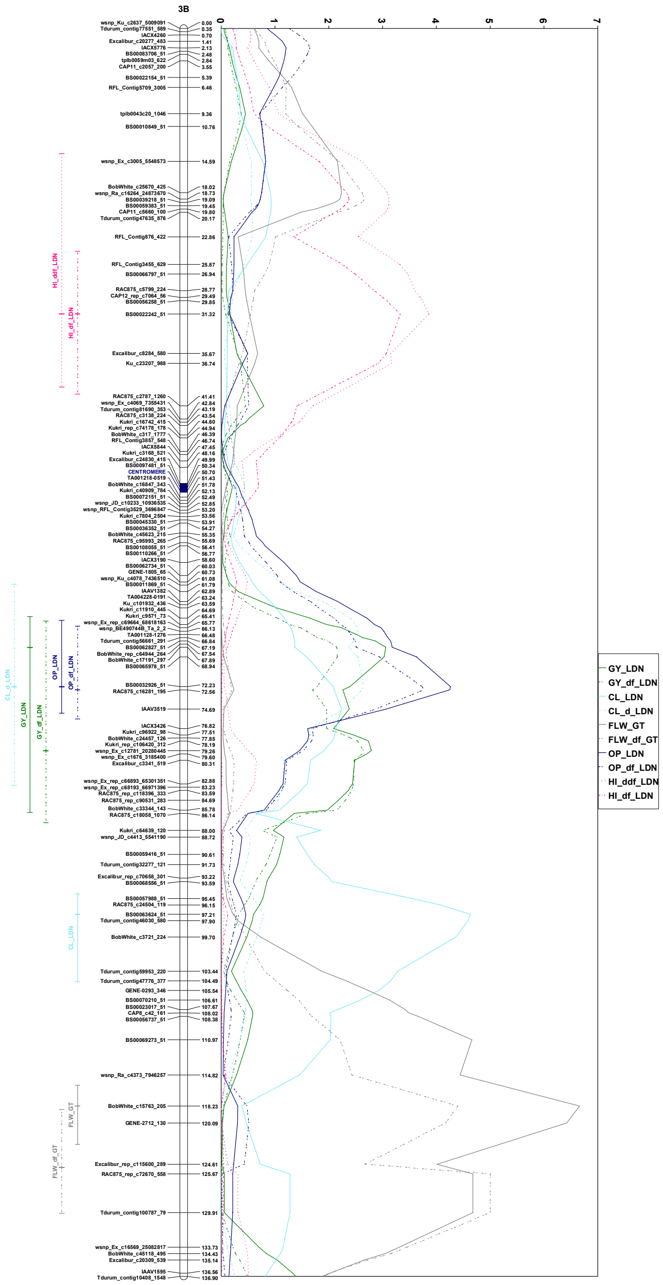

Figure S11 Genetic map, putative genetic positions of target genes, likelihood plots and 1.5 LOD support intervals of QTL effects for chromosome 3B.

Genetic map based on skeletal markers, putative genetic positions of target genes were marked in red. Likelihood plots and 1.5 LOD support intervals were named according to corresponding traits, where first part is name of trait, where name of trait without suffix is name of observed trait (solid line), suffix “df” is marked traits accounting to heading (dash line), suffix “d” is marked plasticity traits trait without accounting to heading (dash line with dots) and suffix “ddf” is marked plasticity traits trait with accounting to heading (dotted line). Last part of name includes association with increase trait value allele: G18-16 allele is named with suffix “GT” and Langdon allele is named with suffix “LDN”.

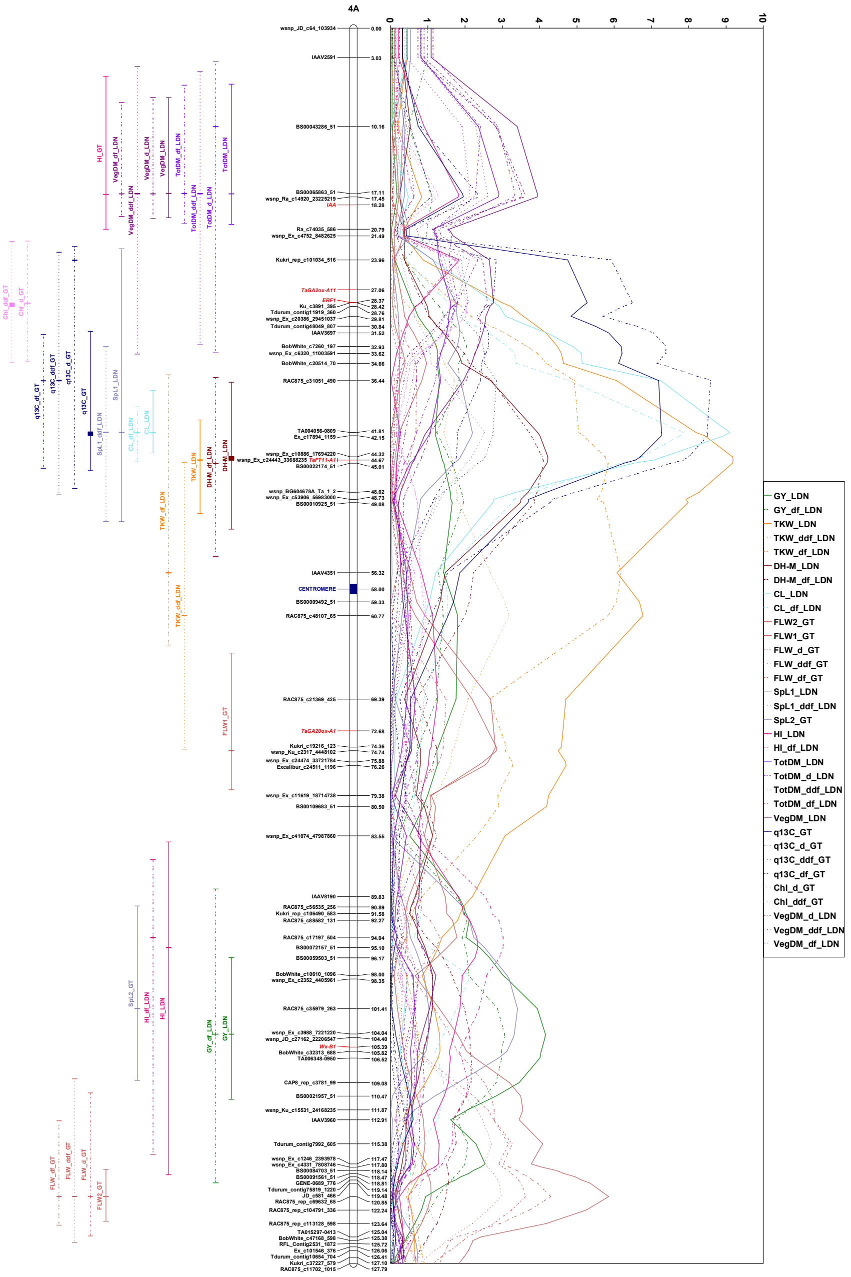

Figure S12 Genetic map, putative genetic positions of target genes, likelihood plots and 1.5 LOD support intervals of QTL effects for chromosome 4A.

Genetic map based on skeletal markers, putative genetic positions of target genes were marked in red. Likelihood plots and 1.5 LOD support intervals were named according to corresponding traits, where first part is name of trait, where name of trait without suffix is name of observed trait (solid line), suffix “df” is marked traits accounting to heading (dash line), suffix “d” is marked plasticity traits trait without accounting to heading (dash line with dots) and suffix “ddf” is marked plasticity traits trait with accounting to heading (dotted line). Last part of name includes association with increase trait value allele: G18-16 allele is named with suffix “GT” and Langdon allele is named with suffix “LDN”.

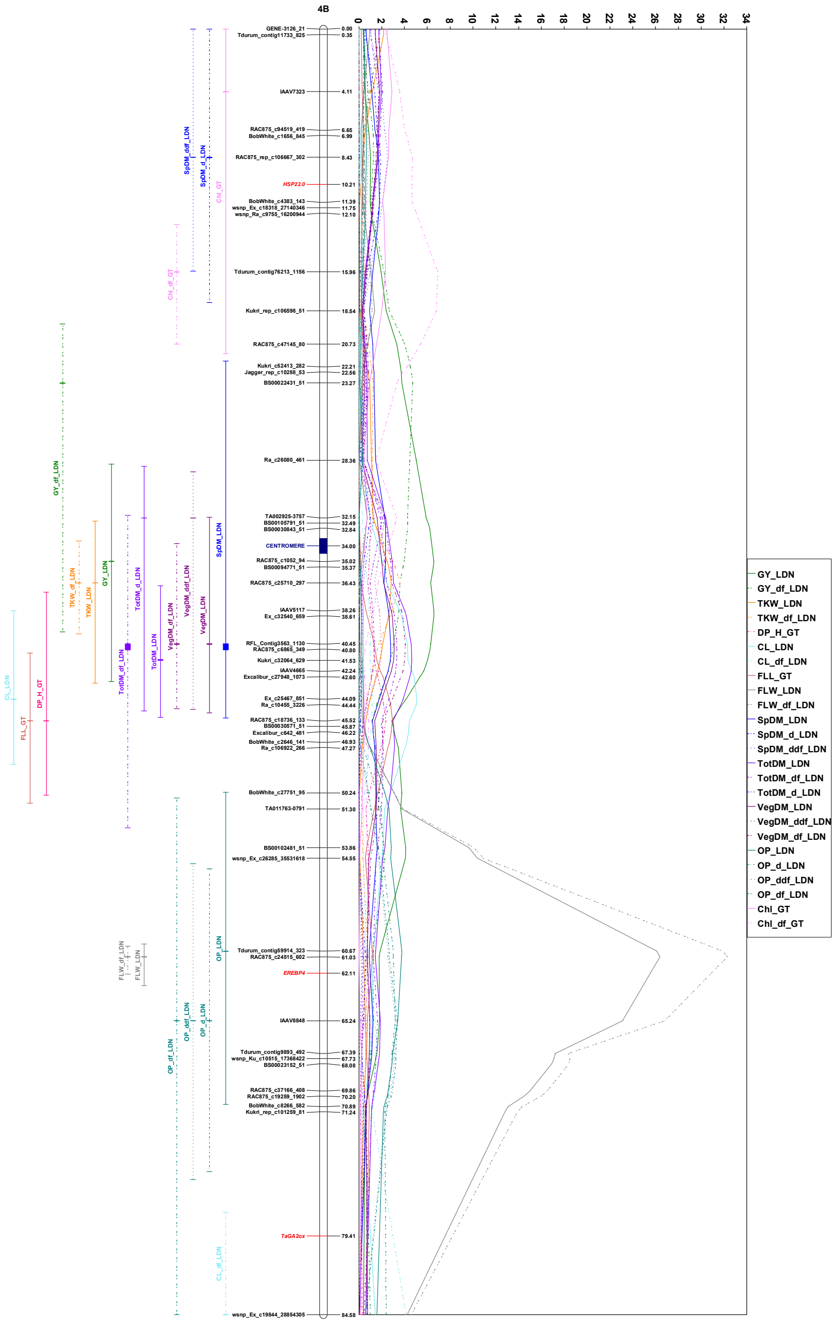

Figure S13 Genetic map, putative genetic positions of target genes, likelihood plots and 1.5 LOD support intervals of QTL effects for chromosome 4B.

Genetic map based on skeletal markers, putative genetic positions of target genes were marked in red. Likelihood plots and 1.5 LOD support intervals were named according to corresponding traits, where first part is name of trait, where name of trait without suffix is name of observed trait (solid line), suffix “df” is marked traits accounting to heading (dash line), suffix “d” is marked plasticity traits trait without accounting to heading (dash line with dots) and suffix “ddf” is marked plasticity traits trait with accounting to heading (dotted line). Last part of name includes association with increase trait value allele: G18-16 allele is named with suffix “GT” and Langdon allele is named with suffix “LDN”.

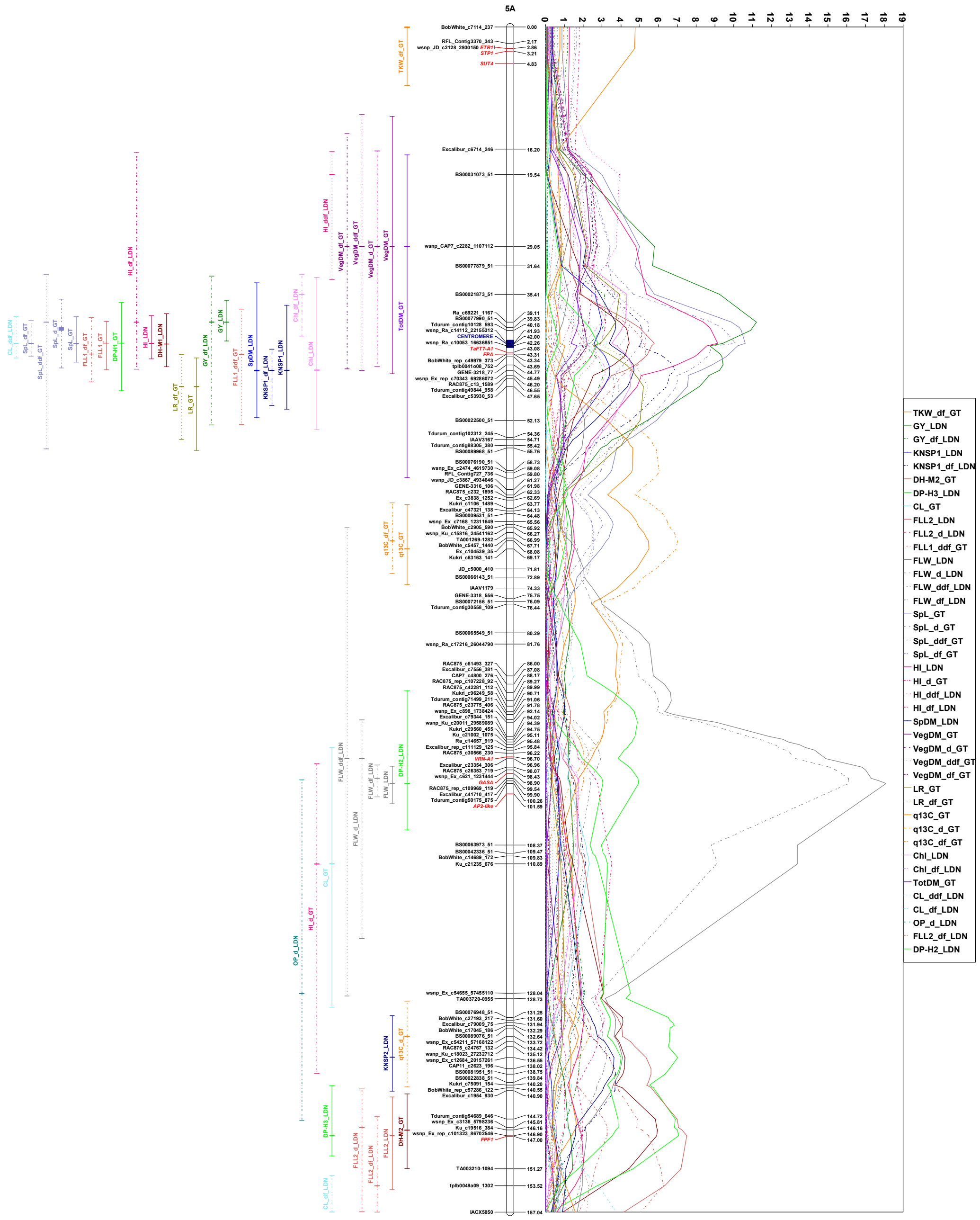

Figure S14 Genetic map, putative genetic positions of target genes, likelihood plots and 1.5 LOD support intervals of QTL effects for chromosome 5A.

Genetic map based on skeletal markers, putative genetic positions of target genes were marked in red. Likelihood plots and 1.5 LOD support intervals were named according to corresponding traits, where first part is name of trait, where name of trait without suffix is name of observed trait (solid line), suffix “df” is marked traits accounting to heading (dash line), suffix “d” is marked plasticity traits trait without accounting to heading (dash line with dots) and suffix “ddf” is marked plasticity traits trait with accounting to heading (dotted line). Last part of name includes association with increase trait value allele: G18-16 allele is named with suffix “GT” and Langdon allele is named with suffix “LDN”.

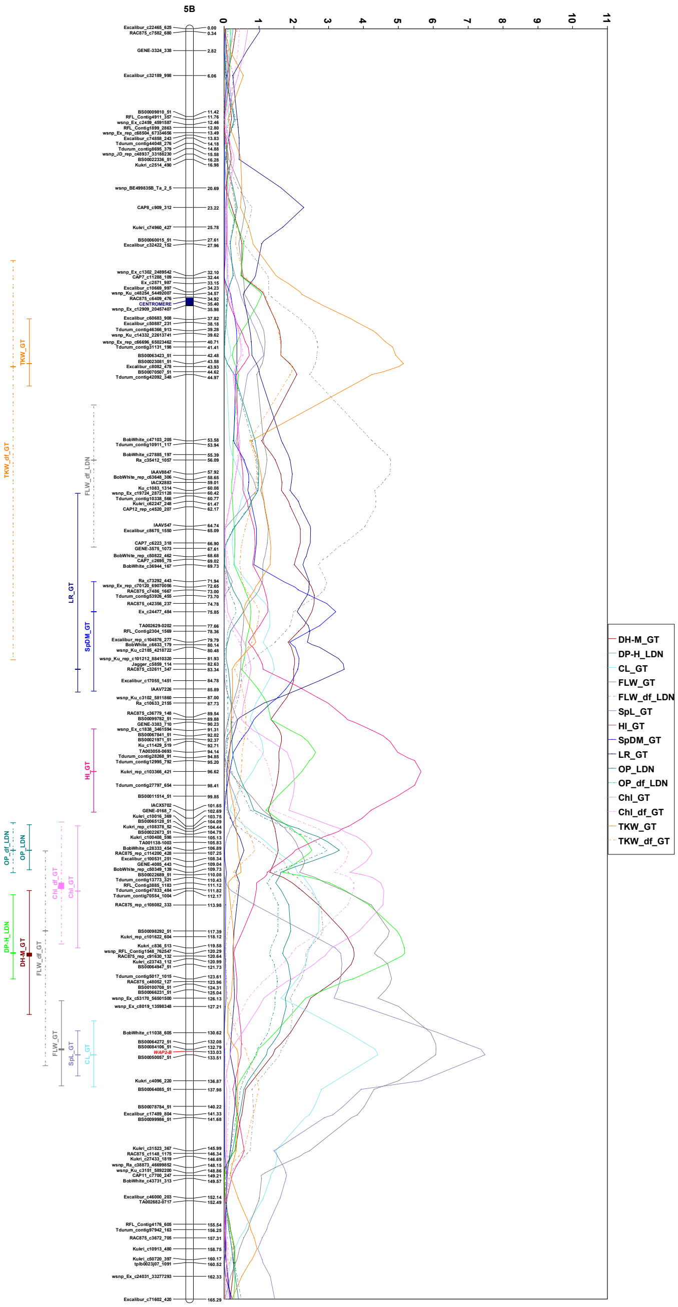

Figure S15 Genetic map, putative genetic positions of target genes, likelihood plots and 1.5 LOD support intervals of QTL effects for chromosome 5B.

Genetic map based on skeletal markers, putative genetic positions of target genes were marked in red. Likelihood plots and 1.5 LOD support intervals were named according to corresponding traits, where first part is name of trait, where name of trait without suffix is name of observed trait (solid line), suffix "df" is marked traits accounting to heading (dash line), suffix "d" is marked plasticity traits trait without accounting to heading (dash line with dots) and suffix "ddf" is marked plasticity traits trait with accounting to heading (dotted line). Last part of name includes association with increase trait value allele: G18-16 allele is named with suffix "GT" and Langdon allele is named with suffix "LDN".

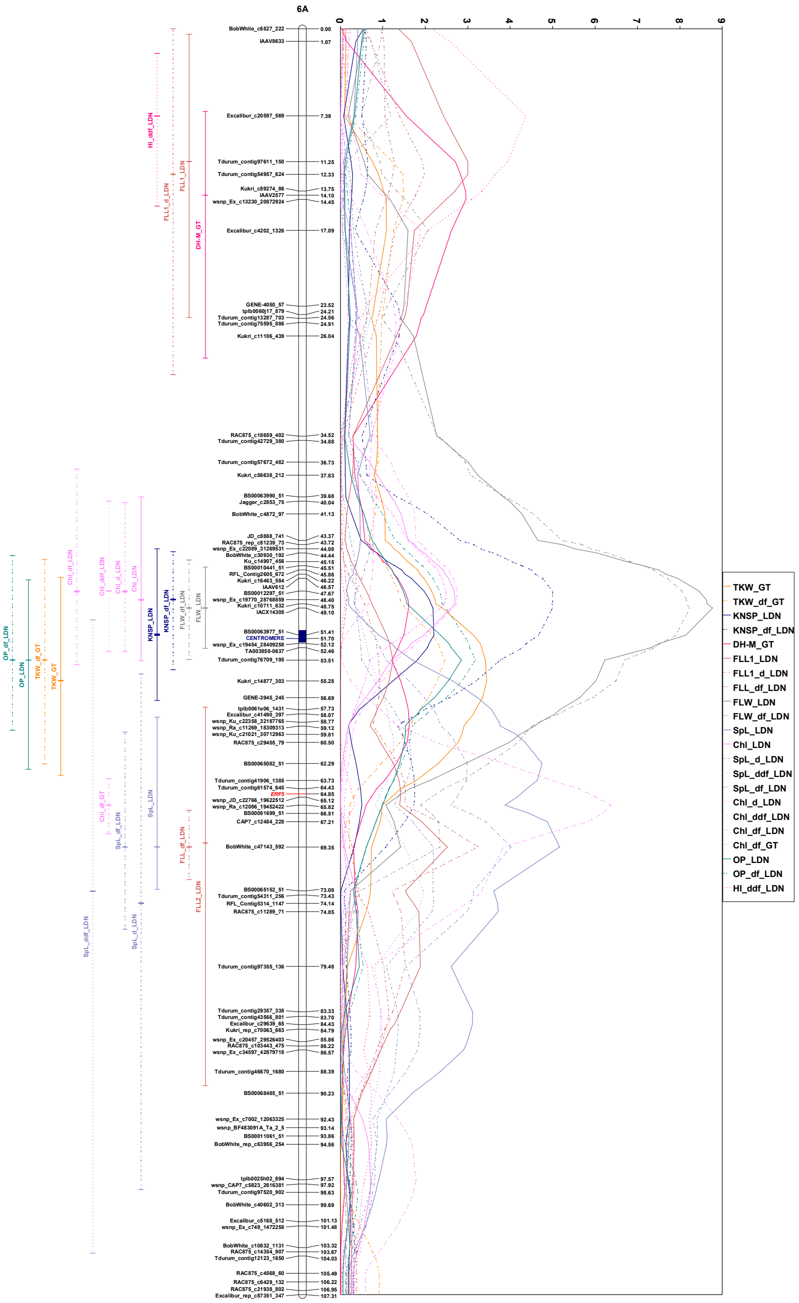

Figure S16 Genetic map, putative genetic positions of target genes, likelihood plots and 1.5 LOD support intervals of QTL effects for chromosome 6A.

Genetic map based on skeletal markers, putative genetic positions of target genes were marked in red. Likelihood plots and 1.5 LOD support intervals were named according to corresponding traits, where first part is name of trait, where name of trait without suffix is name of observed trait (solid line), suffix "df" is marked traits accounting to heading (dash line), suffix "d" is marked plasticity traits trait without accounting to heading (dash line with dots) and suffix "ddf" is marked plasticity traits trait with accounting to heading (dotted line). Last part of name includes association with increase trait value allele: G18-16 allele is named with suffix "GT" and Langdon allele is named with suffix "LDN".

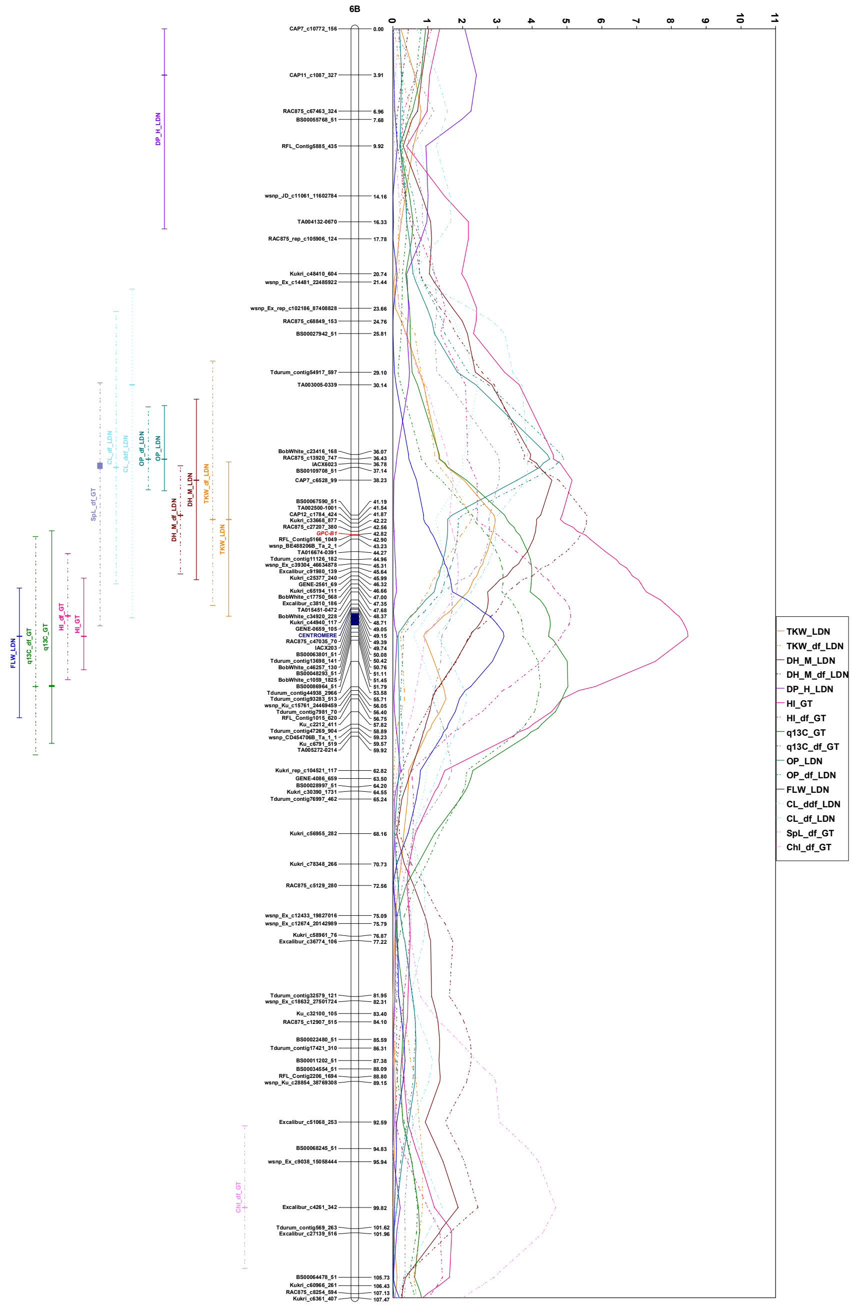

Figure S17 Genetic map, putative genetic positions of target genes, likelihood plots and 1.5 LOD support intervals of QTL effects for chromosome 6B.

Genetic map based on skeletal markers, putative genetic positions of target genes were marked in red. Likelihood plots and 1.5 LOD support intervals were named according to corresponding traits, where first part is name of trait, where name of trait without suffix is name of observed trait (solid line), suffix “df” is marked traits accounting to heading (dash line), suffix “d” is marked plasticity traits trait without accounting to heading (dash line with dots) and suffix “ddf” is marked plasticity traits trait with accounting to heading (dotted line). Last part of name includes association with increase trait value allele: G18-16 allele is named with suffix “GT” and Langdon allele is named with suffix “LDN”.

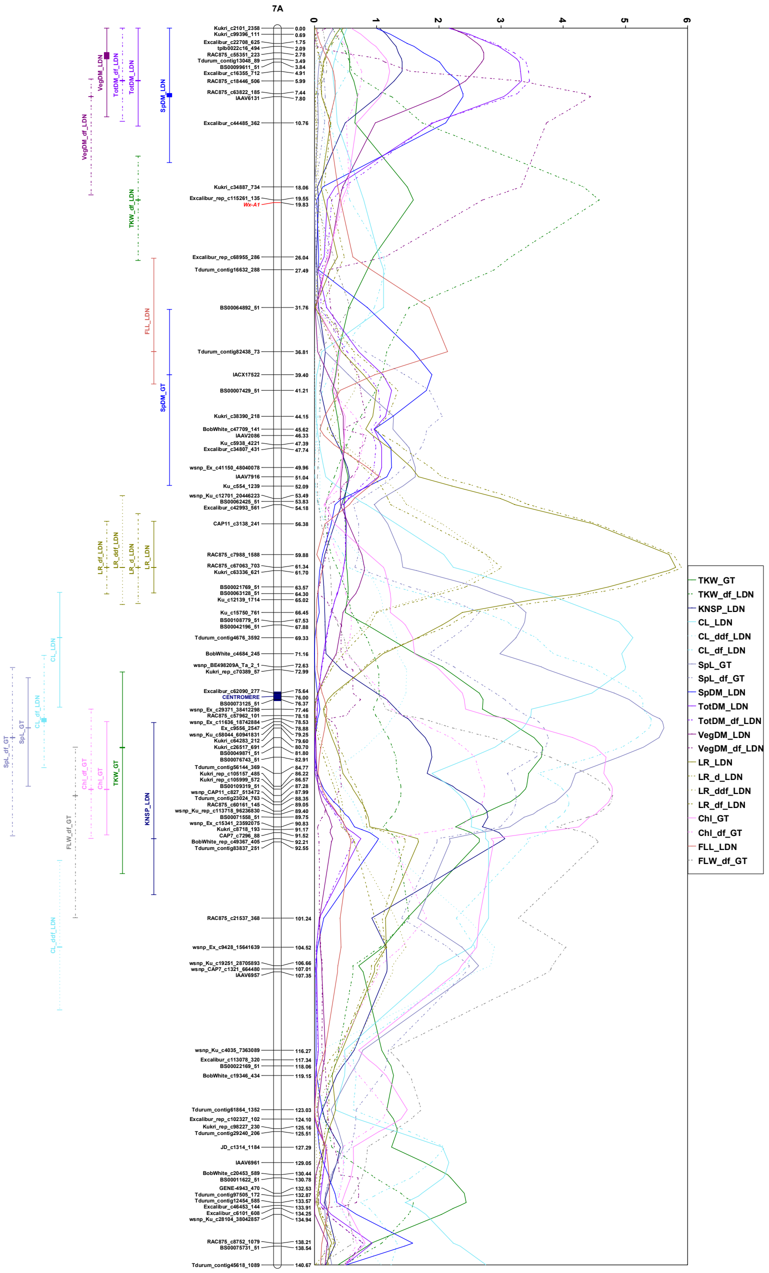

Figure S18 Genetic map, putative genetic positions of target genes, likelihood plots and 1.5 LOD support intervals of QTL effects for chromosome 7A.

Genetic map based on skeletal markers, putative genetic positions of target genes were marked in red. Likelihood plots and 1.5 LOD support intervals were named according to corresponding traits, where first part is name of trait, where name of trait without suffix is name of observed trait (solid line), suffix "df" is marked traits accounting to heading (dash line), suffix "d" is marked plasticity traits trait without accounting to heading (dash line with dots) and suffix "ddf" is marked plasticity traits trait with accounting to heading (dotted line). Last part of name includes association with increase trait value allele: G18-16 allele is named with suffix "GT" and Langdon allele is named with suffix "LDN".

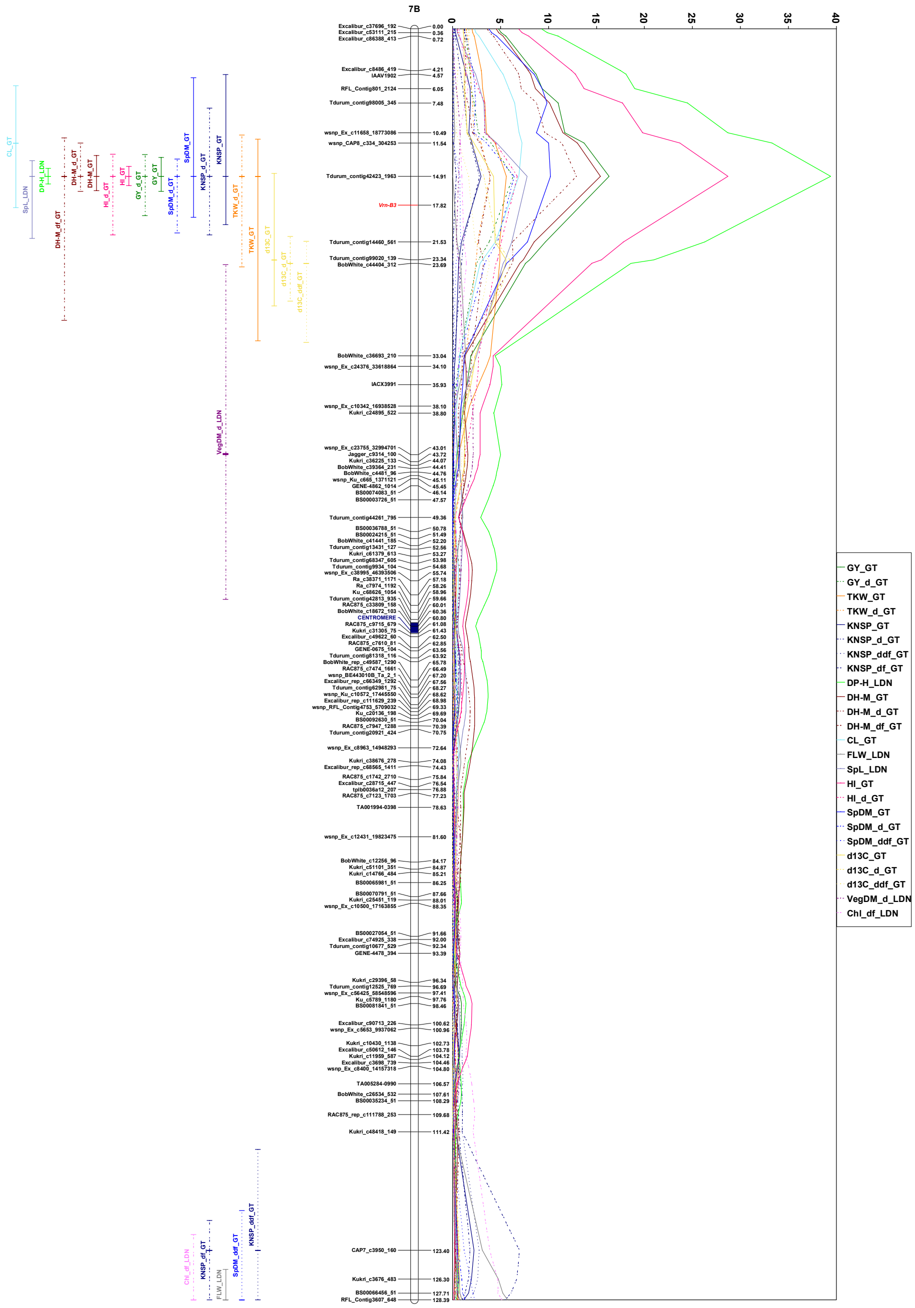

Figure S19 Genetic map, putative genetic positions of target genes, likelihood plots and 1.5 LOD support intervals of QTL effects for chromosome 7B.

Genetic map based on skeletal markers, putative genetic positions of target genes were marked in red. Likelihood plots and 1.5 LOD support intervals were named according to corresponding traits, where first part is name of trait, where name of trait without suffix is name of observed trait (solid line), suffix “df” is marked traits accounting to heading (dash line), suffix “d” is marked plasticity traits trait without accounting to heading (dash line with dots) and suffix “ddf” is marked plasticity traits trait with accounting to heading (dotted line). Last part of name includes association with increase trait value allele: G18-16 allele is named with suffix “GT” and Langdon allele is named with suffix “LDN”.
